# IL-4/IL-13 Axis in Allergic Rhinitis: Elevated Serum Cytokines Levels and Inverse Association With Tight Junction Molecules Expression

**DOI:** 10.3389/fmolb.2022.819772

**Published:** 2022-03-17

**Authors:** Siti Muhamad Nur Husna, Norasnieda Md Shukri, Sharifah Emilia Tuan Sharif, Hern Tze Tina Tan, Noor Suryani Mohd Ashari, Kah Keng Wong

**Affiliations:** ^1^ Department of Immunology, School of Medical Sciences, Universiti Sains Malaysia, Kubang Kerian, Malaysia; ^2^ Department of Otorhinolaryngology, Head and Neck Surgery, School of Medical Sciences, Universiti Sains Malaysia, Kubang Kerian, Malaysia; ^3^ Hospital Universiti Sains Malaysia, Kubang Kerian, Malaysia; ^4^ Department of Pathology, School of Medical Sciences, Universiti Sains Malaysia, Kubang Kerian, Malaysia

**Keywords:** allergic rhinitis, IL-4, IL4R, IL-13, IL13RA1, tight junction

## Abstract

The IL-4/IL-13 axis is involved in the pathogenesis of allergic rhinitis (AR). In this study, we investigated the serum cytokines levels of IL-4, IL-5, IL-6, and IL-13 in AR patients, and the transcript expression levels of their receptors (*i.e. IL4R*, *IL5RA*, *IL6R,* and *IL13RA1*) in nasal epithelial cells of AR patients *versus* non-allergic controls. Nasal epithelial cells and blood samples of non-allergic controls (*n* = 30) and AR patients (*n* = 30) were collected to examine mRNA expression and serum cytokines levels, respectively. Bioinformatics analyses of IL-4/IL-13 receptor heterodimer association with tight junction (TJ) and JAK/STAT signaling genes were conducted in a gene expression profiling (GEP) dataset (GSE44037) of AR patients (*n* = 12) and healthy controls (*n* = 6). Serum IL-4, IL-5, IL-6 or IL-13 levels, and *IL13RA1* transcript expression were significantly higher in AR patients compared with non-allergic controls. IL-4 and IL-13 serum levels were positively correlated with *IL13RA1* expression in AR patients but not in non-allergic controls. In the GEP dataset (GSE44037), six TJ (*CLDN4*, *CLDN7*, *CLDN12*, *CLDN15*, *TJP1*, and *TJP2*) genes’ expressions were negatively correlated, respectively, with IL-4Rα/IL-13Rα1 heterodimeric receptor expression in AR patients and not in control samples. These six TJ genes contributed to the significant enrichment of tight junction Gene Ontology (GO ID: 0070160). Lastly, STATs DNA binding motif analysis showed that each of these TJ genes contains STATs binding consensus sequence within intronic and intergenic regions. Our results suggest that increased IL-4/IL-13 serum cytokines levels may contribute to decreased TJs expression via IL-4Rα/IL-13Rα1 heterodimeric receptor in nasal epithelium of AR patients.

## Introduction

Allergic rhinitis (AR) is a common chronic inflammatory disease with high prevalence across different regions of the globe (Greiner et al., 2011; Pawankar, 2014; Sani et al., 2019; Tan et al., 2020). AR is characterized by aberrantly high Th2 cytokines levels such as interleukin-4 (IL-4), IL-5, IL-6, and IL-13 (Nur Husna et al., 2021a). IL-4 and IL-13 are key pathogenic Th2 cytokines in AR where they activate B cells to synthesize IgE, induce goblet cell hyperplasia, trigger airway hyperresponsiveness, and mucus hypersecretion (Wills-Karp and Finkelman, 2008; Sahoo et al., 2016; Bezerra Barros et al., 2020). IL-5 exerts pleiotropic effects on eosinophils by promoting their maturation, activation, survival, migration from bloodstream, and recruitment to airways in allergic disease (Abo et al., 2019; Boberg et al., 2020). IL-6 is a growth and differentiation factor for T and B cells, and it promotes the production of IgE (Gubernatorova et al., 2018).

Th2 cytokines modulate their signal through binding with their specific receptors that activate downstream signaling pathways. IL-4 and IL-13 are structurally similar, multifunctional peptides, and share a functional signaling receptor chain. IL-4 binds to two receptors *i.e.* the type I (composed of IL-4Rα and common γ-chain) and type II (composed of IL-4Rα and IL-13Rα1) IL-4 receptors. Binding of IL-4 with its type I receptor comprises of IL-4Rα and γ_c_ chain leads to activation of Janus kinase 1 (JAK1) and JAK3, respectively. For type II receptor, binding of IL-13 with IL-13Rα1 subunit activates TYK2/JAK2 (Bieber, 2020; Nguyen et al., 2020) that phosphorylates the tyrosine residues at the cytoplasmic tail of IL-4Rα that in turn serves as docking sites for signal transducer and activator of transcription 6 (STAT6) (Junttila, 2018; Gasparini et al., 2020). This activates IL-4 and IL-13 responsive genes in the subsequent signaling pathway of allergic responses.

Receptor for IL-5 is IL-5Rα where it relies on the β subunit to mediate the biological activities of IL-5. IL-5 binds to a ligand-specific α subunit associated with the common βc receptor subunit (common β subunit). Subsequent signaling pathways are activated including JAK/STAT, MAPK, PI3K, and NF-κB important in regulating the activities of eosinophils (Pelaia et al., 2018; Kandikattu et al., 2019). On the other hand, IL-6 binds with its cognate receptor IL-6Rα, and the complex associates with gp130 initiating their dimerization and consequent activation of JAK kinases. These mediate the phosphorylation of specific tyrosine residues on the gp130 cytoplasmic tail, which in turn acts as docking sites for STAT3 (and STAT1) SH2 domains, leading to JAK-mediated STAT3 phosphorylation and dimerization that further regulates transcription of target genes (Camporeale and Poli, 2012; Ham et al., 2018; Bin Dhuban et al., 2019).

Multiple studies have indicated the disruption of nasal epithelial barrier as the underlying cause of AR (Takano et al., 2005; Steelant et al., 2018; Nur Husna et al., 2021a). It has been proposed that Th2 cytokines signal through their respective receptors on nasal epithelial cells to suppress expression of TJs (Steelant et al., 2018; Nur Husna et al., 2021a). However, there is a lack of literature on the expression of interleukin receptors in nasal epithelial cells of AR patients. Therefore, our study was undertaken to investigate the serum cytokine levels of IL-4, IL-5, IL-6, and IL-13, the transcript expression levels of *IL4R*, *IL5RA*, *IL6R,* and *IL13RA1* in AR patients *versus* non-allergic controls, and the correlation between IL-4Rα/IL-13Rα1 heterodimeric receptor (each subunit encoded by *IL4R* and *IL13RA1* transcripts) with TJs and JAK/STAT signaling genes in AR patients and healthy controls.

## Materials and Methods

### Study Population

We previously recruited 30 AR patients and 30 non-allergic control subjects between March 2019 and July 2019 who attended the Hospital Universiti Sains Malaysia (HUSM) for our TJ genes expression project (Nur Husna et al., 2021b). Briefly, inclusion criteria for AR patients included a diagnosis of moderate/severe AR, positive skin prick test (SPT) to house dust mite (HDM) allergen and ≥18 years old, while non-allergic controls did not have signs or symptoms of allergy, no personal and immediate family history of allergic diseases, negative SPT to HDM allergen and ≥18 years old. The clinico-demographical characteristics of the recruited subjects are presented in Supplementary Table S1. Nasal brushing to collect nasal epithelial cells and venous blood were taken from each participant. All subjects involved in this study provided written and signed informed consent. The protocols were approved by the Human Research Ethics Committee of Universiti Sains Malaysia (JEPeM) (approved ethics code: USM/JEPeM/18060273). The samples were labeled anonymously, and all data were recorded and analyzed anonymously. All procedures were conducted in accordance with our institutional ethical standards and regulations, and with the 1964 Declaration of Helsinki and its later updates.

### Blood Samples Collection

Five ml of peripheral blood samples were collected from AR patients and non-allergic controls into a 6 ml plain blood tube. The blood was stored for 1 hour at room temperature to allow samples to clot. The blood was then centrifuged at 3,500xg for 4 min to obtain the serum after separation from fibrinogen and cells. Serum samples were collected and stored at −80°C until further use.

### Nasal Epithelial Cells Collection, RNA Extraction and Reverse Transcription-PCR (RT-PCR)

Cytology brush (Citotest Labware Co., Ltd., Haimen City, China) was used to collect nasal epithelial cells by fully inserting the brush into the nostrils and rubbed a few times against the medial and superior side of the inferior nasal meatus, using rotatory and linear movements. RNA was extracted from the samples using the RNeasy Mini Kit (Qiagen, Hilden, and Germany) and reverse-transcribed using iScript Reverse Transcription (RT) Supermix for RT-qPCR (Bio-Rad, Philadelphia, PA, United Sates) according to manufacturer’s protocols.

### Quantitative PCR (qPCR)

qPCR was conducted using iTaq Universal SYBR Green Supermix (Bio-Rad, Philadelphia, PA, United Sates) and primers (Integrated DNA Technologies, Singapore) were designed by using the NCBI Primer-BLAST (https://www.ncbi.nlm.nih.gov/tools/primer-blast/) as presented in Supplementary Table S2. For every gene investigated, at least one primer (forward or reverse) was designed to encompass an exon-exon junction to avoid potential genomic DNA amplification, and the BLAST results of each primer was assessed to confirm the absence of alignment with genes other than the gene of interest. The Mx3005P qPCR thermal cycler (Agilent Technologies, CA, United Sates) was used in the qPCR reaction. Each qPCR reaction mixture constituted the following: 1) 10 μl of iTaq Universal SYBR Green Supermix (2x) for a final concentration of 1x; 2) 2 μl (400 nM) each for forward and reverse primer; 3) cDNA template at a final concentration of 50 ng in 20 μl; 4) Nuclease-free water added into a final volume of 20 μl qPCR reaction was subsequently conducted for 40 cycles with the following thermal profile: 1) Polymerase activation step at 95°C (25 s); 2) Denaturation step at 95°C (5 s); 3) Annealing and extension steps at 60°C (20 s). The relative transcript levels of every target gene in each sample was calculated using the 2^−ΔΔCt^ formula in which ΔΔCt = [(Ct sample–Ct control)—ΔCt1].

### Measurement of Cytokines Levels With Magnetic Luminex® Assay

The levels of Th2 cytokines (IL-4, IL-5, IL-6, and IL-13) were measured using Magnetic Luminex^®^ Assay (R&D System, Minneapolis, United Sates). Prior to measurement of Th2 cytokines, the serum sample was thawed and centrifuged at 16,000xg for 4 min. The serum was diluted to a 2-fold dilution (*i.e.* 75 μl of sample plus 75 μl of calibrator diluent RD6-52) and was mixed thoroughly. Standards, controls and samples were added per well. The diluted microparticle cocktail was resuspended by inversion and vortexing. A total of 50 μl of the microparticle cocktail was added into each well of the microplate. The microplate was securely covered with a foil plate sealer. The microplate was incubated for 2 h at room temperature on a horizontal orbital microplate shaker (0.12” orbit) set at 800 ± 50 rpm. The microplate was then washed using magnetic bead washer (BioTek Instruments Inc., Winooski, United Sates). The magnet was applied to the bottom of the microplate for 1 min before removing the liquid. Each well was filled with 100 μl washing buffer for 1 min before discarding. The washing steps were repeated three times.

A total of 50 μl of diluted biotin-antibody cocktail was then added into all wells. The microplate was securely covered again with a foil plate sealer and incubated for 1 h at room temperature on the shaker (800 ± 50 rpm). The washing steps were repeated before 50 μl of diluted streptavidin-PE was added, the microplate securely covered with a foil plate sealer and incubated for 30 min at room temperature on the shaker set at 800 ± 50 rpm. The washing steps were repeated before microparticles were resuspended in 100 μl of washing buffer and incubated for 2 min on the shaker (800 ± 50 rpm). The microplate was subsequently read within 90 min using the Luminex® 200™ analyzer (Luminex, Austin, United Sates).

### Correlation Analysis of Th2 Cytokine Receptors With TJ and JAK/STAT Signaling Pathway Genes

A microarray gene expression profiling (GEP) dataset (GSE44037) of nasal epithelial cells derived from 18 adults (>18 years old) comprising of 12 AR and six healthy controls (Wagener et al., 2013) were obtained from the Gene Expression Omnibus database. The similarities and differences between our AR cohort of patients with those of GSE44037 are as follows: 1) Similarities: Above 18 years old; Moderate/severe AR; Allergic status was assessed by SPT; AR patients were sensitized to at least one allergen; AR patients did not use any anti-allergy medications for at least 1 month before samples were collected; 2) Differences: Our cohort was sensitized to HDM allergens while GSE44037 dataset was sensitized to common allergens; AR patients with SPT wheal size of ≥4 mm in our cohort was considered as positive SPT instead of SPT wheal size of >3 mm.

In this GEP dataset, Pearson correlation values were calculated according to the expression of IL-4 and IL-13 receptor heterodimer [*i.e. IL4R* (probe ID: 203233_PM_at) and *IL13RA1* (201887_PM_at) subunits], IL-5 receptor heterodimer [*i.e. IL5RA* (211516_PM_at) and *CSF2RB* (205159_PM_at) subunits], or IL-6 receptor heterodimer [*i.e. IL6R* (205945_PM_at) and *IL6ST* (204863_PM_s_at) subunits] against the total number of probes (*n* = 41,796) present in the microarray platform. These were conducted in AR patients (*n* = 12) or healthy controls (*n* = 6) separately. Then, presence of TJ and JAK/STAT signaling genes with significant (*p* < 0.05) Pearson correlation values (*i.e.* r > |0.603| for 12 AR subjects; r > |0.812| for six healthy control subjects) with both receptor subunit in each receptor heterodimer were shortlisted and demonstrated in Pearson correlation scatter plots. The TJ genes or desmosomal genes examined were claudins (*CLDN1*, *CLDN2*, *CLDN3*, *CLDN4*, *CLDN5*, *CLDN6*, *CLDN7*, *CLDN8*, *CLDN9*, *CLDN10*, *CLDN11*, *CLDN12*, *CLDN13*, *CLDN14*, *CLDN15*, *CLDN16*, *CLDN17*, *CLDN18*, *CLDN19*, *CLDN20*, *CLDN21*, *CLDN22*, and *CLDN23*), junctional adhesion molecules (JAMs; *JAM1*, *JAM2*, and *JAM3*), desmogleins (*DSG1*, *DSG2*, *DSG3*, and *DSG4*), desmocollins (*DSC1*, *DSC2*, and *DSC3*), cadherins (*CDH1*, *CDH2*, *CDH3*, and *CDH12*), and zonula occludens (ZO; *TJP1*, *TJP2*, and *TJP3*). The genes involved in JAK/STAT signaling examined were *JAK1*, *JAK2*, *JAK3*, *STAT1*, *STAT2*, *STAT3*, *STAT6*, and *LTK* (TYK1 gene name) and *TYK2*. Gene Ontology (GO) enrichment analysis of genes negatively correlated with *IL4R* and *IL13RA1* expression was conducted using the ToppGene database as described previously (Chen et al., 2009; Brown et al., 2016).

### Statistical Analysis

Data were analyzed using Student’s t-test (for normally distributed data) and Mann-Whitney U test (for not normally distributed data) to determine the difference of gene expression between AR and non-allergic control groups. Shapiro-Wilk test was conducted to assess the normality of the data. The relationship between two variables with continuous data was examined with Pearson correlation. All analyses were conducted using GraphPad Prism v6.07 (GraphPad Software Inc., CA, United Sates). All *p*-values were two-tailed and values < 0.05 were considered statistically significant.

## Results

### Sensitization of the AR Patients to HDM Allergens

**FIGURE 1:**
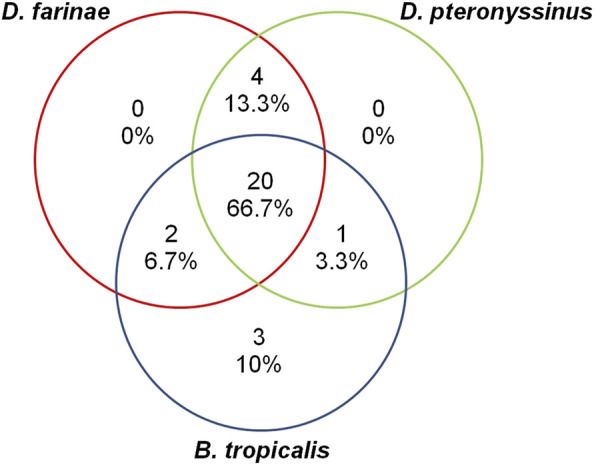
The proportion of AR patients (total *n* = 30) sensitized to HDM allergens *D. farinae*, *D. pteronyssinus*, and *B. tropicalis*.

In this study, we tested the sensitization to HDM allergens of *Dermatophagoides pteronyssinus* (*D. pteronyssinus*)*, Dermatophagoides farinae* (*D. farinae*), and *Blomia tropicalis* (*B. tropicalis*). Only 10% (*n* = 3) of the AR patients were monosensitized to *B. tropicalis* and none of the patients were monosensitized to the other two HDMs. The rest of the 27 AR patients were either sensitized to two (*n* = 7/30; 23.3%) or all three (n = 20/30; 66.7%) of the HDM allergens (Figure 1).

### Serum IL-4, IL-5, IL-6, and IL-13 Levels in AR Patients and Non-allergic Controls

**FIGURE 2:**
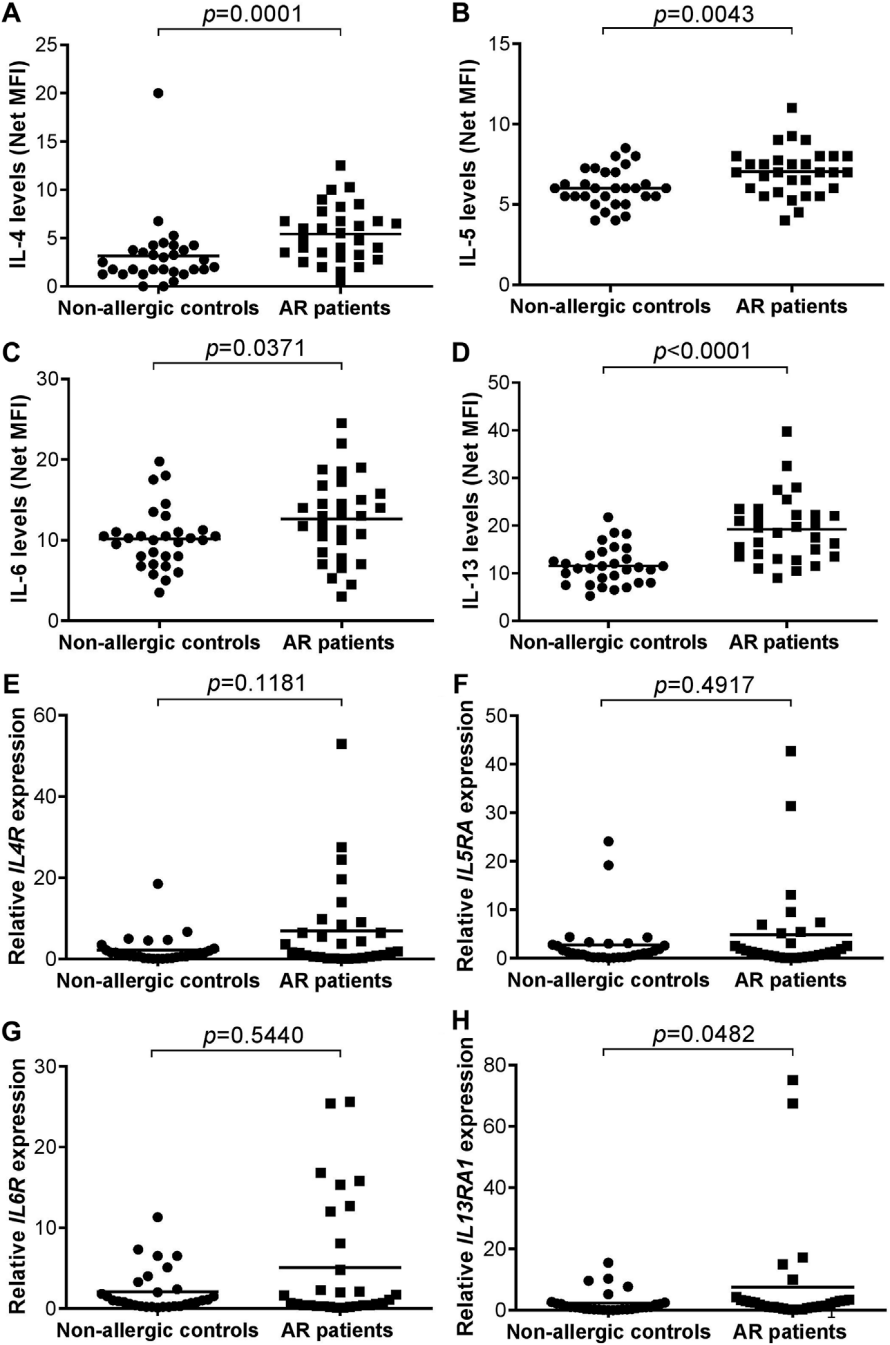
**(A–D)** Serum levels of IL-4 **(A)**, IL-5 **(B)**, IL-6 **(C)**, and IL-13 **(D)** in non-allergic controls (*n* = 30) *versus* AR patients (*n* = 30). Bar represents mean **(E–H)** Relative *IL4R* **(E)**, *IL5RA* **(F)**, *IL6R* **(G)**, and *IL13RA1* **(H)** expression in non-allergic controls (*n* = 30) *versus* AR patients (*n* = 30). Bar represents mean.

The median age of the non-allergic control and AR groups were 25.50 (range: 20–54) and 28.00 (24–54) years old, respectively. The proportion of female subjects was 73.3% (*n* = 22) and 66.7% (*n* = 20) in non-allergic control and AR group, respectively. For the complete demographic and clinical characteristics of both groups of subjects, readers are directed to our recent publication (Nur Husna et al., 2021b). The serum levels of IL-4 (*p* = 0.0001), IL-5 (*p* = 0.0043), IL-6 (*p* = 0.0371) or IL-13 (*p* < 0.0001) were significantly higher in AR patients compared with non-allergic controls (Figures 2A–D).

### Expression of *IL4R*, *IL5RA*, *IL6R,* and *IL13RA1* Transcripts in AR Patients and Non-allergic Controls

No significant difference was observed in the expression of *IL4R* (*p* = 0.118), *IL5RA* (*p* = 0.492) or *IL6R* (*p* = 0.544) transcript in AR patients compared with non-allergic controls (Figures 2E–G). The expression of *IL13RA1* (*p* = 0.048) transcript was borderline significantly higher in nasal epithelial cells of AR patients compared with non-allergic controls due to two outlier AR cases (Figure 2H).

### Correlation of Serum Th2 Cytokine Levels With Their Receptor’s Expression Levels in AR Patients and Non-allergic Controls

The serum levels of IL-4 or IL-13 were positively and significantly associated with the expression of *IL13RA1* transcripts in AR patients (r = 0.4296, *p* = 0.0178 and r = 0.4200, *p* = 0.0208, respectively) (Supplementary Figure S1). Such relationship was not observed in non-allergic controls (r = −0.1969, *p* = 0.2970 and r = −0.3071, and *p* = 0.0988, respectively) (Supplementary Figure S2). The rest of the comparisons including serum IL-4 with *IL4R*, serum IL-5 with *IL5RA*, serum IL-6 with *IL6R*, or serum IL-13 with *IL4R* did not yield a significant relationship in AR patients (Supplementary Figure S1) or non-allergic controls (Supplementary Figure S2).

### Association of *IL4R* and *IL13RA1* Expression With TJ and JAK/STAT Signaling Pathway Genes in AR Patients and Healthy Controls

**FIGURE 3:**
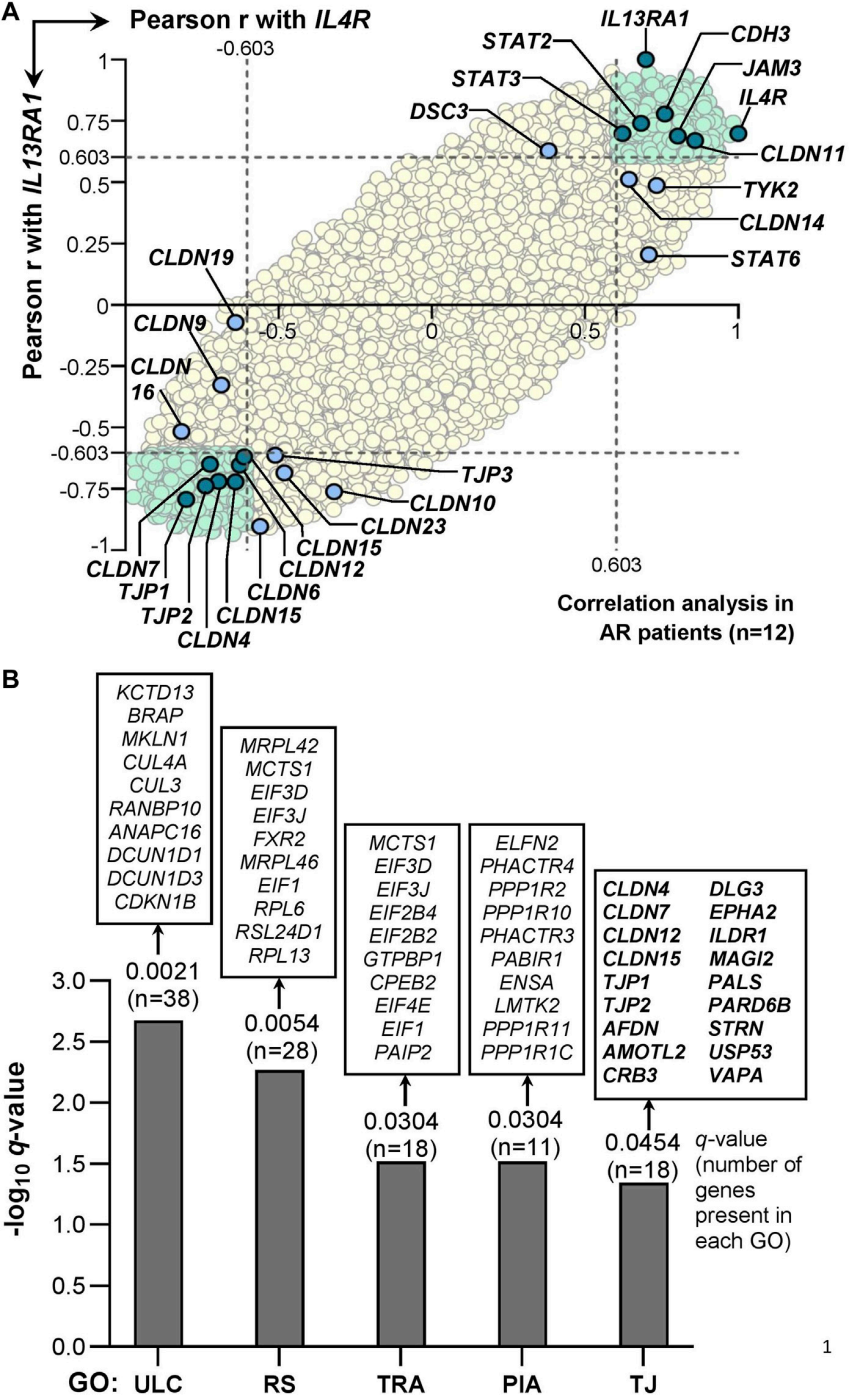
Correlation of IL-4 receptor heterodimer subunits expression (*i.e., IL4R* or *IL13RA1*) with 20,541 annotated genes in AR patients derived from GSE44037 dataset. **(A)** Pearson correlation of the genes expression levels with *IL4R* or *IL13RA1* expression levels in AR patients (*n* = 12). The Pearson r value ± 0.603 is used as the cut-off (i.e. the dotted lines) to define positive (r > 0.603) or negative (r < −0.603) correlation with *IL4R* or *IL13RA1* as this cut-off represents significant (i.e. *p* < 0.05) Pearson correlation for a sample size of 12. All genes significantly correlated with both *IL4R* and *IL13RA1* expression levels are highlighted in light green. TJ, desmosomal or JAK/STAT signaling genes with significant correlation with both *IL4R* and *IL13RA1* expression levels are highlighted in teal. TJ, desmosomal or JAK/STAT signaling genes with significant correlation with either *IL4R* or *IL13RA1* expression levels only are highlighted in light blue. The rest of the background genes are highlighted in yellow. **(B)** Gene Ontology (GO) enrichment analysis of genes inversely associated with *IL4R* and *IL13RA1* expression (GSE44037 dataset). Ten representative genes are displayed on top of each bar, and all 18 genes contributed to the enrichment of TJ ontology (GO ID: 0070160) are shown. ULC: Ubiquitin ligase complex; RS: Ribosomal subunit; TRA: Translation regulator activity; PIA: Phosphatase inhibitor activity; TJ: Tight junction.

Correlation analysis of IL-4/IL-13 receptor heterodimer with TJs or JAK/STAT signaling genes in AR patients (*n* = 12) derived from the GSE44037 GEP dataset showed that a total of 1,382 probes representing 1,088 annotated genes were positively correlated (r > 0.603, *p* < 0.05) with both *IL4R* and *IL13RA1* expression (Supplementary Table S3). These 1,088 genes consisted of the JAK/STAT signaling genes *STAT2* and *STAT3*, as well as certain TJ genes including *CLDN11*, *JAM3*, and *CDH3*. The expression of other JAK/STAT signaling genes (*i.e,. STAT6* and *TYK2*) was also positively correlated with *IL4R* but not with *IL13RA1* expression levels (Figure 3A). On the other hand, a total of 1,646 probes representing 1,325 annotated genes were negatively correlated (r < −0.603, *p* < 0.05) with both *IL4R* and *IL13RA1* expression (Supplementary Table S3). In these 1,325 genes, several TJ genes were implicated including *CLDN4*, *CLDN7*, *CLDN12*, *CLDN15*, *TJP1,* and *TJP2*, while no JAK/STAT genes were involved. Other TJ genes also showed significant negative correlation with either *IL4R* (*CLDN9*, *CLDN16* and *CLDN19*) or *IL13RA1* (*CLDN6*, *CLDN10*, *CLDN23*, and *TJP3*) (Figure 3A).

In healthy controls (n = 6) derived from the same GEP dataset, none of the genes present in the GEP platform demonstrated significant association with both *IL4R* and *IL13RA1* expression levels (Supplementary Figure S3). In terms of desmosomal genes, only *DSG4* was negatively correlated with *IL13RA1* expression and none of TJ and JAK/STAT genes examined showed significant correlation with either *IL4R* or *IL13RA1* expression (Supplementary Figure S3). Interestingly, we observed that none of the TJ or JAK/STAT signaling genes presented with significant correlation with IL-5 receptor heterodimer (*i.e. IL5RA* and *CSF2RB*) or IL-6 receptor heterodimer (*i.e. IL6R* and *IL6ST*) expression in AR or healthy control subjects (Supplementary Figures S3, S5).

### Gene Ontology Enrichment and STATs Consensus Sequence Analysis

To validate whether TJ ontology was enriched, we conducted GO enrichment analysis of genes negatively correlated with both *IL4R* and *IL13RA1* expression (r < −0.603, *p* < 0.05). Six collective groups of GOs were enriched consisting of protein complexes, ribosome components, transcription and translation processes, phosphatases, TJ and other cellular components GOs (Supplementary Table S4). A representative GO from each of the collective group (excluding other cellular components group) was presented in Figure 3B. The TJ ontology (GO ID: 0070160) was enriched (*p* = 0.0010, *q* = 0.0454) where 18 of 134 genes annotated in the ontology were present including *CLDN4*, *CLDN7*, *CLDN12*, *CLDN15*, *TJP1,* and *TJP2* (Figure 3B).

Activation of IL-4Rα/IL-13Rα1 heterodimeric receptor by their cytokines (IL-4 and IL-13) activates the JAK/STAT signaling pathway that leads to the activation of the transcription factor STAT6. IL-4-activated STAT6 has been shown to function as transcriptional repressor (Czimmerer et al., 2018), while IL-13-activated STAT6 enhanced permeability of epithelial cells by altering the expression of several TJ genes (Lin et al., 2019). STAT6 is capable of directly repressing transcription of genes via binding to intronic and intergenic regions of target genes (Takaki et al., 2008; Czimmerer et al., 2018). Taken together, we were interested to assess whether these six TJ genes contain STAT6 (and STATs collectively) consensus binding sequence within their genomic sequences as this may imply that STAT6 is capable of binding and regulating or suppressing their expression. The genomic sequences investigated were retrieved from UCSC Genome Browser (GRCh38/hg38) database.

STAT proteins target and bind the palindromic consensus sequence 5′-TTC(N)_2-4_GAA-3′ where N_2-4_ denotes spacer nucleotides comprising of two (N2), three (N3) or four (N4) nucleotides (Kraus et al., 2003; Goenka and Kaplan, 2011; Li et al., 2016). STAT6 differs from other STAT proteins whereby its binding element comprising of either N3 or N4 site (*i.e,.* 5′-TTC(N)_3/4_GAA-3′), and it preferentially binds N4 over N3 site (*i.e.* 5′-TTC(N)_4_GAA-3′), as well as preferential binding the regions within the first two introns over either upstream of transcription start site or downstream of transcription end site (Elo et al., 2010; Li et al., 2016). Thus, we examined for the presence of 5′-TTC(N)_2–4_GAA-3′ motifs within the following DNA regions of the six TJ genes: 5 kb upstream of the first exon, the first two introns and 5 kb downstream of the last exon. We observed that each gene contains at least five and two 5′-TTC(N)_2-4_GAA-3′ and 5′-TTC(N)_3/4_GAA-3′ motifs, respectively, within these regions (Table 1). In terms of STAT6 preferential motif and regions *i.e,.* 5′-TTC(N)_4_GAA-3′ within the first two introns, four TJ genes contain at least one of such motif in their first two introns *i.e., CLDN7* (*n* = 1), *CLDN12* (*n* = 2), *TJP1* (*n* = 7), and *TJP2* (*n* = 11) (Table 1). For reference, the complete list of the DNA sequences examined and the presence of STATs binding motifs for the six TJ genes are presented in Supplementary Table S5.

**TABLE 1:** Presence of STATs consensus sequence 5′-TTC(N)_2-4_GAA-3′ in DNA regions of TJ genes. Motif within intron 1 is in bold.

Gene (accession number)	5 kb upstream of the first exon			First two introns			5 kb downstream of the last exon		
	N2 spacer	N3 spacer	N4 spacer	N2 spacer	N3 spacer	N4 spacer	N2 spacer	N3 spacer	N4 spacer
*CLDN4* (ENST00000435050.1)	TTCAGGAA	TTCCCAGAA	TTCCTTAGAA	—	—	—	TTCTGGAA	—	—
	TTCCAGAA								
*CLDN7* (ENST00000397317.8)	TTCATGAA	—	—	—	TTCACCGAA	**TTCGAGGGAA**	TTCTGGAA	—	—
	TTCAAGAA								
*CLDN12* (ENST00000400011.6)	TTCTGGGAA	TTCACAGAA	TTCAGAGGAA	TTCTAAGAA	TTCAGAGAA	TTCTTAAGAA	TTCCAGAA	TTCAAGGAA	TTCTATAGAA
	TTCAAGAA			TTCTAGAA	TTCCCTGAA	TTCTAAAGAA			
				TTCAGGAA	TTCAATGAA				
					TTCTTTGAA				
*CLDN15* (ENST00000287916.8)	TTCCTGAA	TTCTAGGAA	—	—	—	—	TTCCAGAA	TTCCCAGAA	—
		TTCGTTGAA					TTCAGGAA		
		TTCGGGGAA					TTCTAGAA		
*TJP1* (ENST00000401528.5)	—	TTCGAGGAA	TTCCCGGGAA	**TTCCTGAA**	TTCTTGGAA	**TTCCTACGAA**	—	TTCTGAGAA	TTCCAAGGAA
				TTCAGGAA	TTCTAAGAA	**TTCAGCCGAA**			
				TTCCTGAA	TTCTTGGAA	TTCTCCTGAA			
				TTCATGAA	TTCAGAGAA	TTCGGGAGAA			
				TTCTTGAA	TTCCAAGAA	TTCAGAGGAA			TTCCTTGGAA
					TTCATTGAA	TTCATTGGAA			
					TTCACTGAA	TTCATTGGAA			
					TTCCTTGAA				
*TJP2* (ENST00000377245.9)	TTCATGAATTCCTGAA	TTCCACGAA	TTCTTGAGAATTCCATTGAA	**TTCTTGAA**	**TTCCCTGAA**	**TTCCAGAGAA**	TTCTTGAATTCTTGAATTCCAGAA	TTCTCAGAATTCCTAGAATTCTGGGAA	TTCCTCAGAA
				**TTCATGAA**	**TTCTTAGAA**	**TTCGTAGGAA**			
				**TTCAAGAA**	**TTCCATGAA**	**TTCTTGAGAA**			
				**TTCCAGAA**	**TTCCATGAA**	**TTCTTTAGAA**			
				**TTCTGGAA**	**TTCGTTGAA**	**TTCCCAGGAA**			
				TTCCCGAA	**TTCTTTGAA**	**TTCAGTGGAA**			
				TTCCTGAA	**TTCTCAGAA**	**TTCATGGGAA**			
					**TTCTCAGAA**	**TTCAAAGGAA**			
					**TTCAGGGAA**	**TTCTATGGAA**			
					**TTCATAGAA**	**TTCACCTGAA**			
					**TTCATTGAA**	TTCCTTGGAA			
					TTCTATGAA				

**FIGURE 4:**
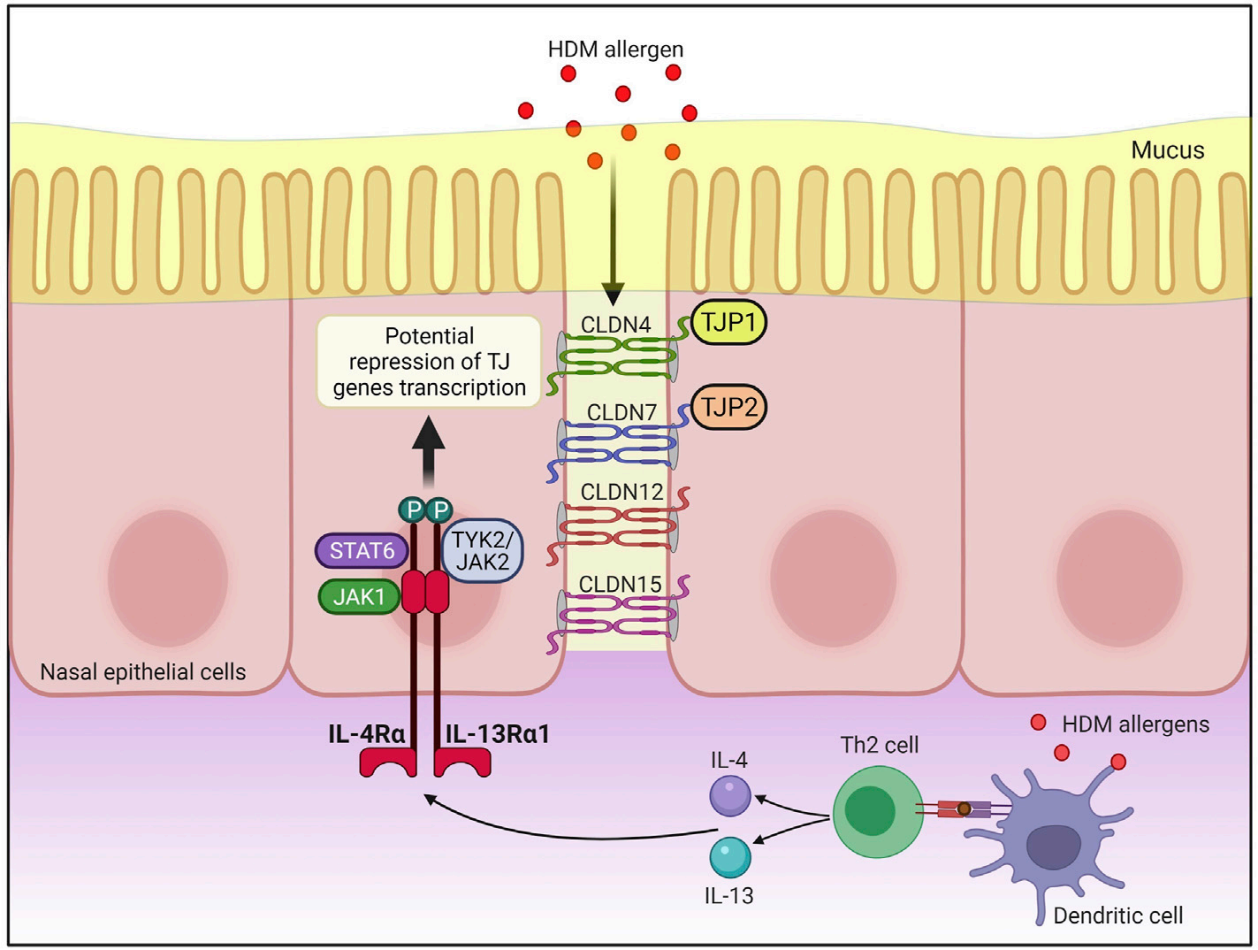
Antigen presenting cells (APCs) such as dendritic cells uptake, process and present peptides from allergens on the major histocompatibility complex (MHC) class II molecule. The antigen complex and the MHC class II molecule serve as a ligand for T cell receptors on naïve CD4^+^ T cells, resulting in differentiation of naïve CD4^+^ T cells into allergen-specific Th2 cell. Subsequently, IL-4 and IL-13 are produced by activated Th2 cells, allowing IL-4 and IL-13 to bind to IL-4Rα/IL-13Rα1 heterodimeric receptor. This activates Janus kinase 1 (JAK1) on IL-4Rα and TYK2/JAK2 on IL-13Rα1 which phosphorylates the tyrosine residues at the cytoplasmic tail of IL-4Rα that in turn serves as docking sites for signal transducer and activator of transcription 6 (STAT6). Activated STAT6 may then repress the transcription of *CLDN4*, *CLDN7*, *CLDN12*, *CLDN15*, *TJP1*, and *TJP2* expression in the nasal epithelial cells of AR patients. CLDN, claudin; HDM, house dust mite; JAK, Janus kinase; STAT6, signal transducer, and activator of transcription 6; Th2, T helper 2; TJP, tight junction protein; TJs, tight junctions; TYK, tyrosine kinase. Created with BioRender.com.

A graphical representation of the IL-4/IL-13 axis cascade and the potential pathways involving STAT6 and the TJ genes implicated in this study is presented in Figure 4. Finally, the protein-protein interaction network between IL-4/IL-13 axis and STAT6, or between TJ proteins is presented in Supplementary Figures 6A, 6B, respectively.

## Discussion

Th2 cytokines enable a continuous inflammation in the nasal mucosa and infiltrate within the sinonasal microenvironment that alter the composition of epithelial tight junctions (TJs) (Capaldo and Nusrat, 2009; London et al., 2016). All AR patients in this study presented with systemic atopy as defined by positive SPT to at least one of the examined aeroallergens (Rondón et al., 2014). Hence, analysis of serum Th2 cytokines levels, instead of local (nasal) levels of Th2 cytokines, was investigated to corroborate with the systemic manifestation. Significantly higher serum IL-4 and IL-5 levels were observed in AR patients compared to control group (*p* < 0.01) (Bi et al., 2018). In a nasal allergen challenge (NAC) study of AR patients sensitized with grass pollen, the frequency of IL-4^+^CD4^+^ T cells were significantly increased at sixth hour after NAC when compared to the control day (Shamji et al., 2015). IL-5 expression was decreased in AR patients with reduced nasal symptoms and infiltration of eosinophils into the nasal mucosa (Sato et al., 2016). AR mice models also demonstrated a significantly higher serum levels of IL-4, IL-5, and IL-13 than in the control group (Cheng et al., 2021). Furthermore, IL-5 was secreted from resident cells in response to *ex vivo* allergen challenge in the AR group but not in the non-allergic group, indicating that local presentation of antigen to resident allergen-specific Th2 cells represents early events of AR pathogenesis (Skrindo et al., 2015). In terms of IL-6, small nucleotide polymorphism (SNP) affecting *IL-6* (rs1800795) was linked with an increased risk of AR (Zhao et al., 2016) and positively associated with the severity of AR (Zhao et al., 2018). These findings are comparable with our results where we demonstrated significantly higher levels of IL-4, IL-5, IL-6, and IL-13 in serum of AR patients compared with non-allergic controls.

We also examined the expression of *IL4R*, *IL5RA*, *IL6R,* and *IL13RA1* in nasal epithelial cells of AR patients and non-allergic controls. However, only *IL13RA1* showed a significant increase in nasal epithelial cells of AR patients compared with non-allergic controls. In our previous study, we demonstrated a significant decrease in the mRNA expression of TJ proteins such as occludin, *CLDN3* and *CLDN7* in nasal epithelial cells of AR patients compared with non-allergic controls (Nur Husna et al., 2021b), and the samples that we used were the same as the current study. These suggest that *IL13RA1* might be involved in the disruption of TJs as IL-13Rα1 is a high-affinity heterodimer receptor that binds IL-4 and IL-13, and both of these cytokines are important factors for decreased nasal epithelial barrier integrity (Wise et al., 2014; Steelant et al., 2016; Wawrzyniak et al., 2017).

Our correlation analysis of GEP dataset demonstrated that the expression profiles of *IL4R* and *IL13RA1* were positively correlated with JAK/STAT signaling genes, and inversely correlated with several claudins and ZO (*i.e. TJP1*, *TJP2*, and *TJP3* transcripts) expression levels in AR patients but not in healthy controls. The TJ GO was also enriched in the group of genes negatively associated with both *IL4R* and *IL13RA1* expression. This includes *CLDN7* and *TJP1* which we and other groups had previously shown that their expression was significantly downregulated in AR patients compared with non-allergic controls (Lee et al., 2016; Nur Husna et al., 2021b). Nasal epithelial barrier disruption induced by the activation of IL-4 or IL-13 receptors has been reported to occur in AR. Firstly, pertaining to IL-4, pre-treatment of Calu-3 (human airway epithelial cells) with anti-IL-4Rα monoclonal antibody (mAb) suppressed the effects of IL-4 and prevented IL-4-induced disruption of epithelial barrier. This was shown to occur via decreased ZO-1 expression, and pretreatment of HDM-challenged mice with anti-IL-4 mAb prevented the loss of ZO-1 expression (Steelant et al., 2018). In terms of IL-13, overexpression of *miR-143* in IL-13-stimulated nasal epithelial cells (NECs) from AR patients suppressed the production of IL-13-induced inflammatory cytokines, and *miR-143* rendered these effects by directly targeting and repressing *IL13RA1* transcript expression (Teng et al., 2015). Treatment of human NECs with the cytokine suppressed the expression of TJ molecules including ZO-1 and CLDN3 (Huang et al., 2020). Moreover, correlation analysis of our cohort of AR and non-allergic control groups showed that serum IL-4 and IL-13 levels were positively associated with *IL13RA1* expresion.

Six of the seven TJ genes (*CLDN4*, *CLDN6*, *CLDN7*, *CLDN12*, *TJP1*, and *TJP2*), which demonstrated negative association with both *IL4R* and *IL13RA1* expression levels, have been implicated in AR such as significantly decreased expression in nasal epithelium of AR patients compared with non-allergic controls (Brandner, 2016; Siti Sarah et al., 2020; Nur Husna et al., 2021a). Moreover, our correlation analysis observation corroborates with the increased expression of *Tjp2*, *Cldn7,* and *Cldn15* in epithelial cells of *Stat6* ^−/−^ mice (Lin et al., 2019). Our literature search did not yield publications implicating CLDN11, JAM3 or CDH3 in AR. Nevertheless, *JAM3* mRNA expression was downregulated in chronic rhinosinusitis patients with nasal polyps *versus* healthy controls (Cornet et al., 2019). The positive association of the three TJ genes, particularly *CLDN11* and *JAM3*, with both *IL4R* and *IL13RA1* expression levels in AR patients remains unexplained and it represents fertile grounds for future investigations and validation.

Binding of IL-4 or IL-13 to their receptor complex, IL-4Rα and IL-13Rα1, induces the JAK/STAT signaling pathway where phosphorylation of transcription factors particularly STAT6 occurs and its subsequent translocation to nucleus to regulate genes expression. Direct transcriptional repression by STAT6 has been reported and this is achieved through STAT6 binding to intronic and intergenic regions (Takaki et al., 2008; Czimmerer et al., 2018). Our STAT6 DNA binding motif analysis showed that *CLDN7*, *CLDN12*, *TJP1,* and *TJP2* contain STAT6 binding consensus sequence (5′-TTC(N)_4_GAA-3′) within its preferential binding regions *i.e.,* the first two introns (Elo et al., 2010; Li et al., 2016). It remains to be elucidated mechanistically whether IL-4 and IL-13 signaling pathway through their receptors, IL-4Rα and IL-13Rα1, may induce epithelial barrier disruption by suppressing TJs expression via STAT6 transcriptional repression activities in AR patients.

We acknowledge the limitations of the study as follows: 1) The AR patients included in this study were sensitized to HDMs only. As the AR patients’ recruitment period in this study was conducted during the classical pollen season, we were unable to rule out the contribution of pollens sensitization to the observations in our study. However, HDM-induced AR is the most common factor causing allergic sensitization among AR patients in Malaysia (Ho et al., 1995; Liam et al., 2002). In particular, over 90% of Malaysian AR patients were positive to *D. pteronyssinus* sensitization while less than 10% of the AR patients were positive to either pollen species (*i.e.*, Bermuda grass or *Acacia sp.*) (Liam et al., 2002); 2) The mRNA expression levels of the interleukin receptors investigated in this study, particularly *IL13RA1*, require confirmation at the protein levels in AR patients *versus* non-allergic controls; 3) Our study is dependent on GEP dataset to draw presumptions between IL-4 and IL-13 with their cognate receptors (IL-4Rα/IL-13Rα1 heterodimeric receptor) and TJ or desmosomal genes, and GEP does not necessarily correlate with functional proteins. Nonetheless, the GEP dataset was derived from human samples and our correlation observations in AR patient samples were compared with those of healthy controls; 4) Our study focused on serum cytokine levels to better mirror systemic atopy as presented by our cohort of AR patients, however this was at the expense of not measuring nasal cytokine levels. Hence, it remains unknown if the effects on the nasal epithelial integrity in our AR patients were also reflected by changes in the nasal Th2 cytokine levels.

Treatment with anti-IL-4Rα mAb is a novel strategy for AR patients. Dupilumab is a fully human anti-IL-4Rα mAb that blocks both IL-4 and IL-13 signaling (Rabe et al., 2018). In a recent randomized, double-blind and placebo-controlled phase IIb clinical trial of dupilumab in perennial AR patients, improved responses were observed in these patients in terms of nasal symptoms (Weinstein et al., 2018), highlighting that the IL-4/IL-13 axis as a novel therapeutic target in the disease.

In summary, we have demonstrated significant elevation of IL-4, IL-5, IL-6, and IL-13 serum levels in AR patients, and increased expression of *IL13RA1* transcripts in nasal epithelial cells of AR patients compared with non-allergic controls. Together with the correlation analyses of TJs and JAK/STAT signaling genes expression with *IL4R* and *IL13RA1*, our findings suggest that IL-4/IL-13 axis may deregulate nasal epithelial barrier integrity by suppressing TJs expression in AR patients, and this warrants future investigation and validation.

## Data Availability

The original contributions presented in the study are included in the article's Supplementary Material, further inquiries can be directed to the corresponding author.
