# Supplementary material for: IL-4/IL-13 Axis in Allergic Rhinitis: Elevated Serum Cytokines Levels and Inverse Association With Tight Junction Molecules Expression
Source: Front Mol Biosci. 2022 Mar 17;9:819772. doi: 10.3389/fmolb.2022.819772 (PMC8969661; doi:10.3389/fmolb.2022.819772)

Supplementary Figure S1

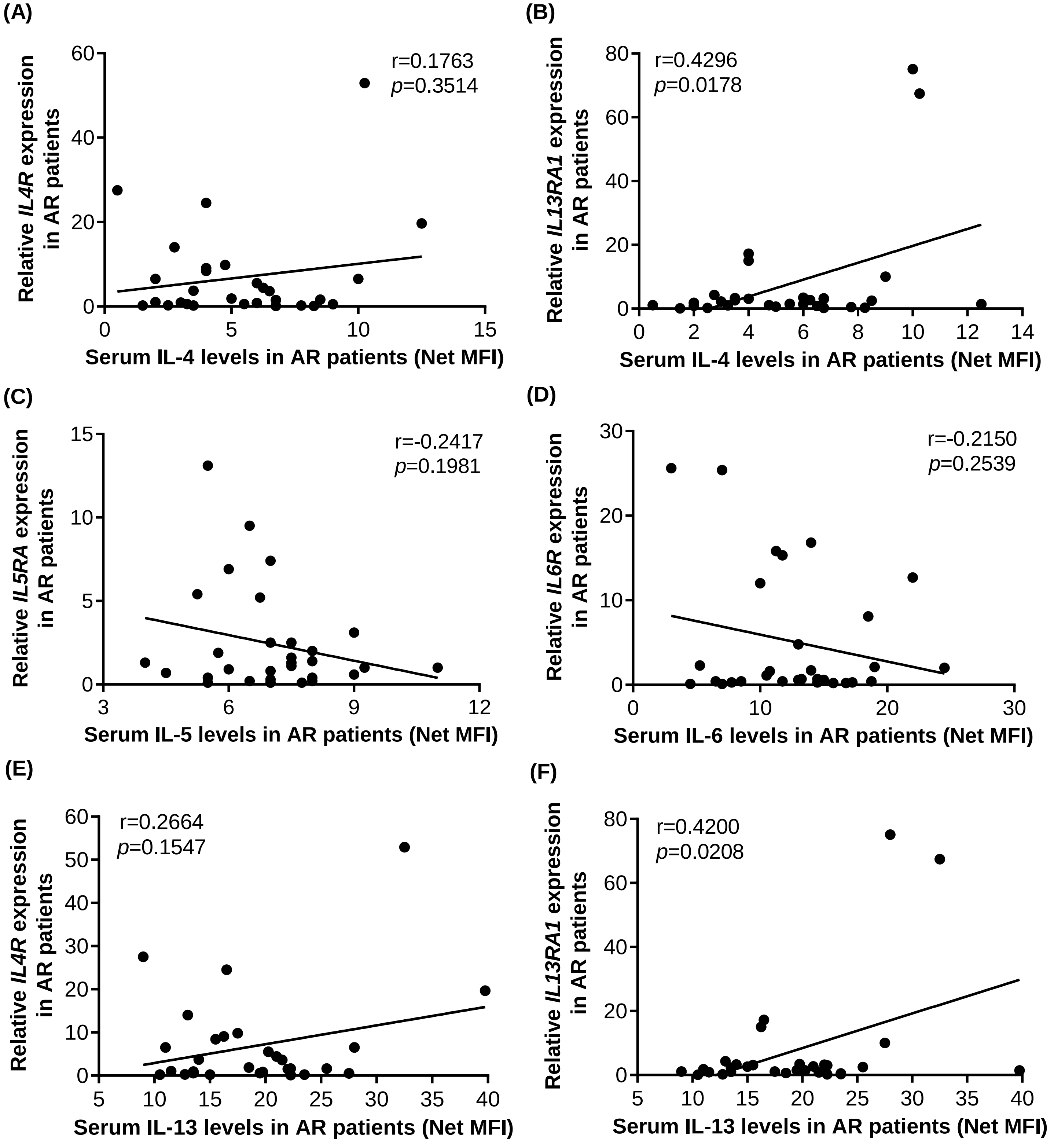

Supplementary Figure S2

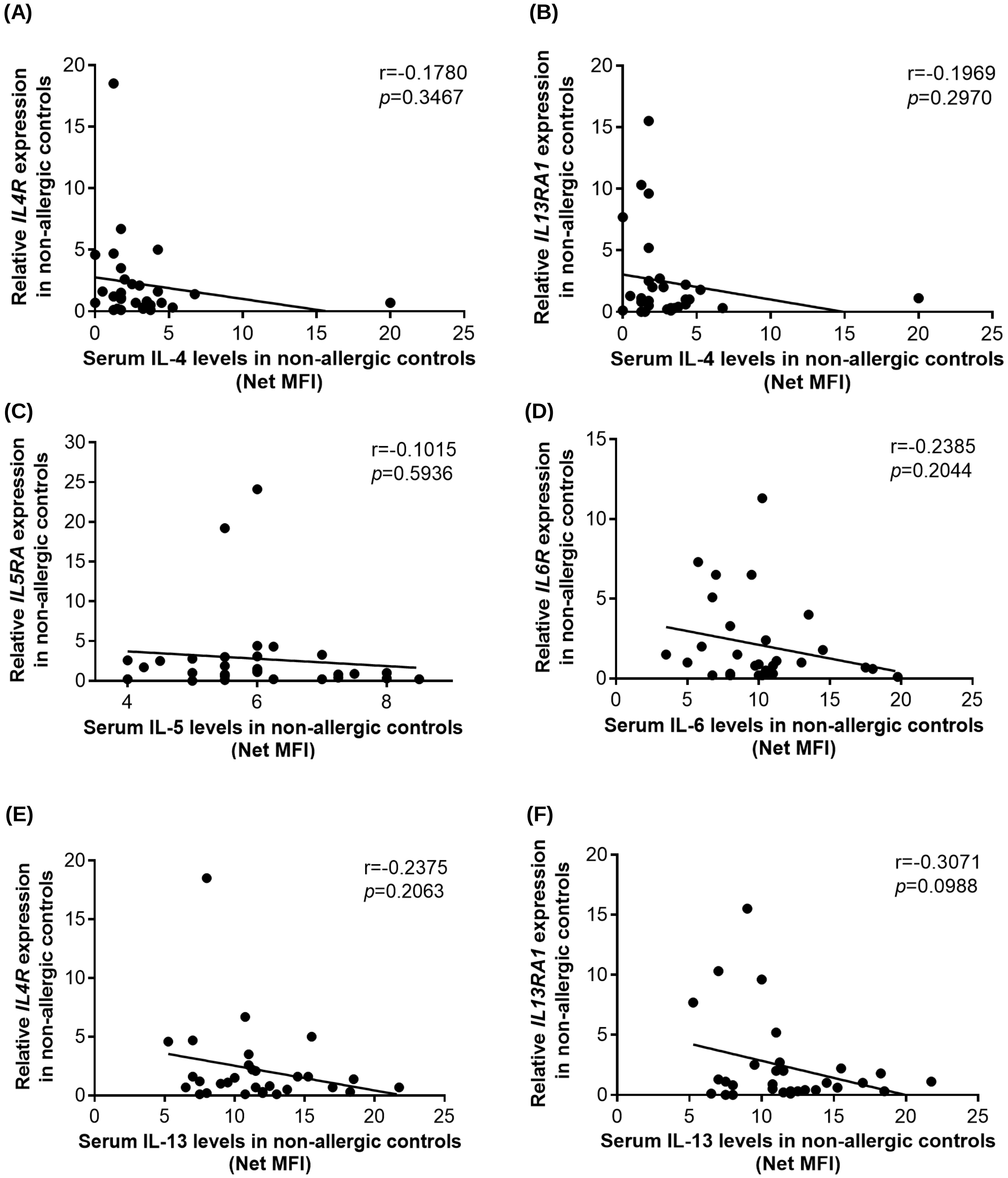

Supplementary Figure S3

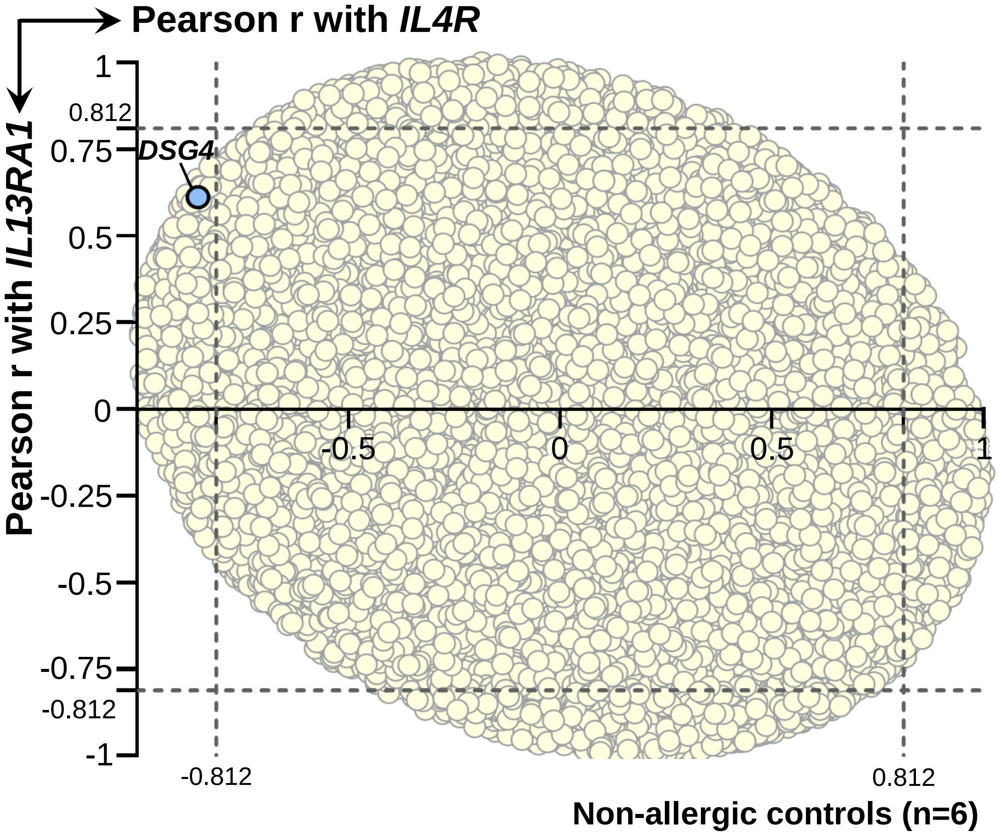

Supplementary Figure S4

(A)

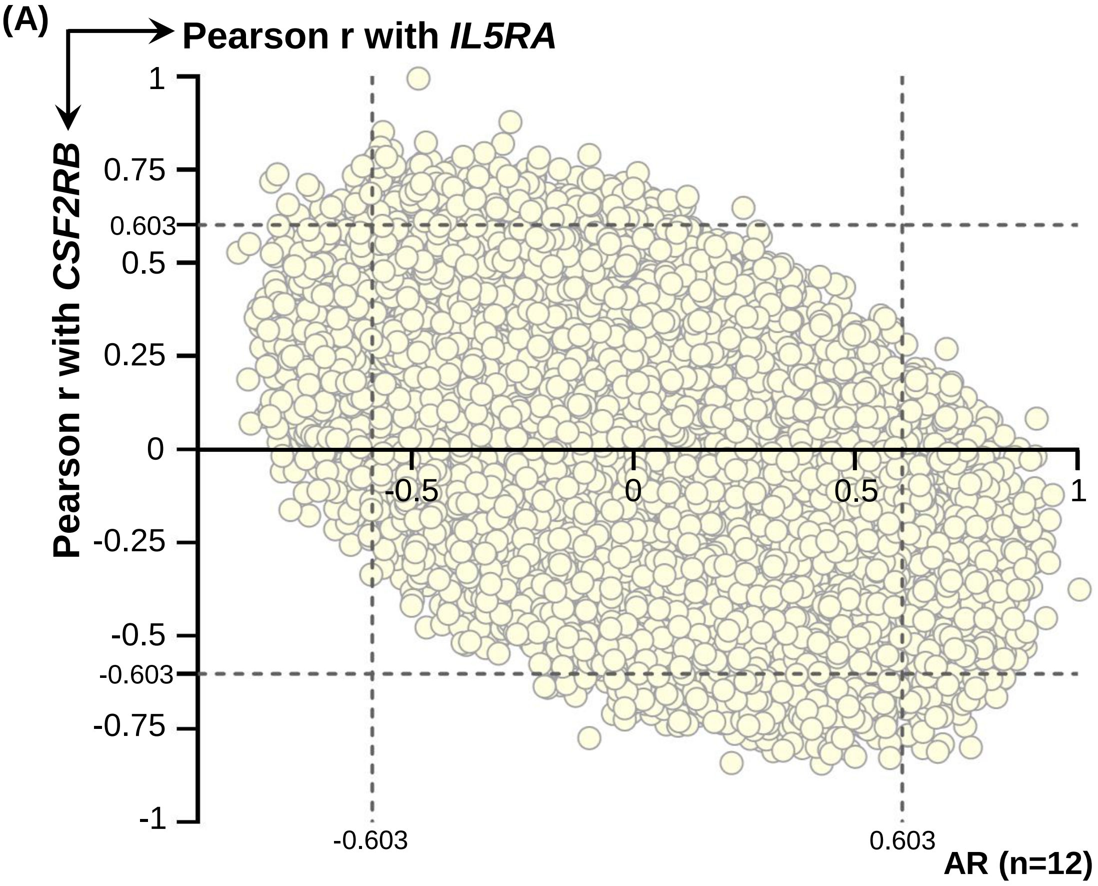

(B)

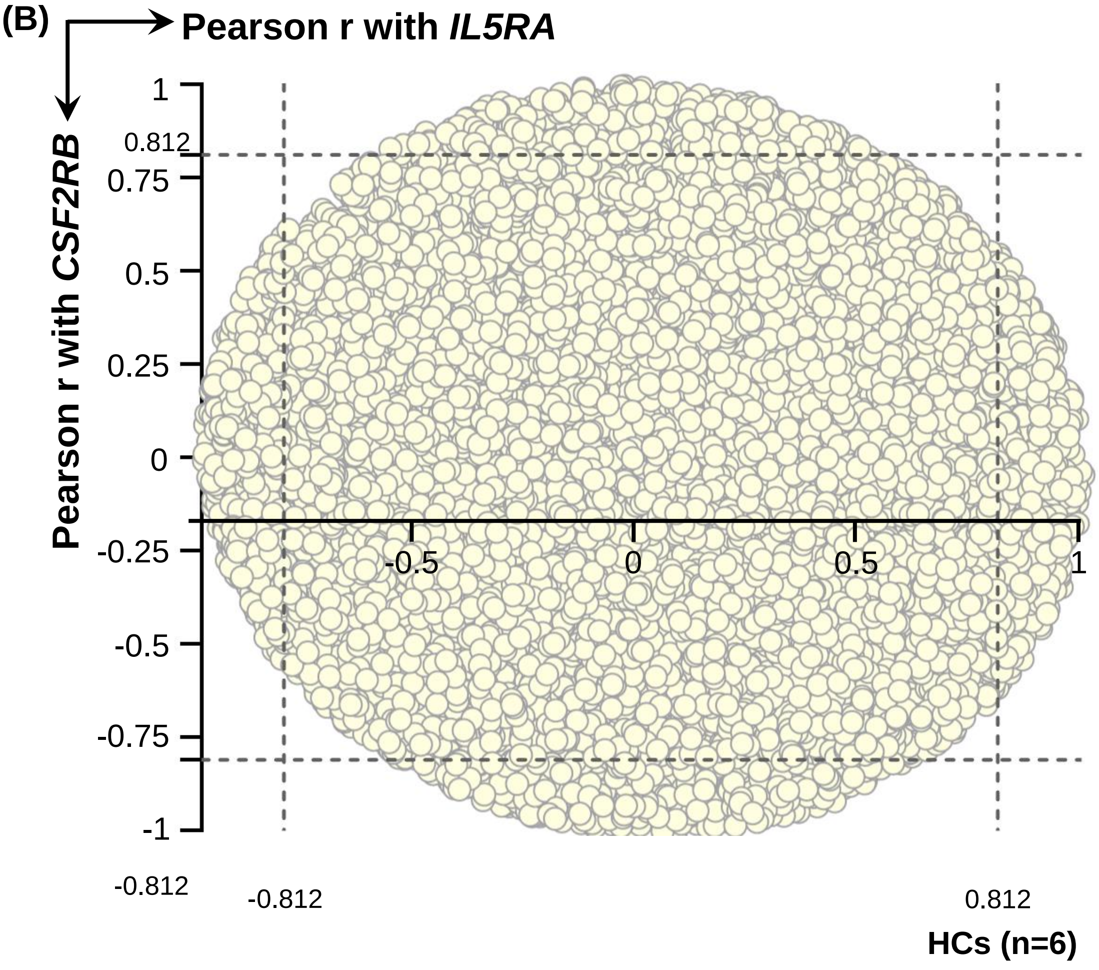

Supplementary Figure S5

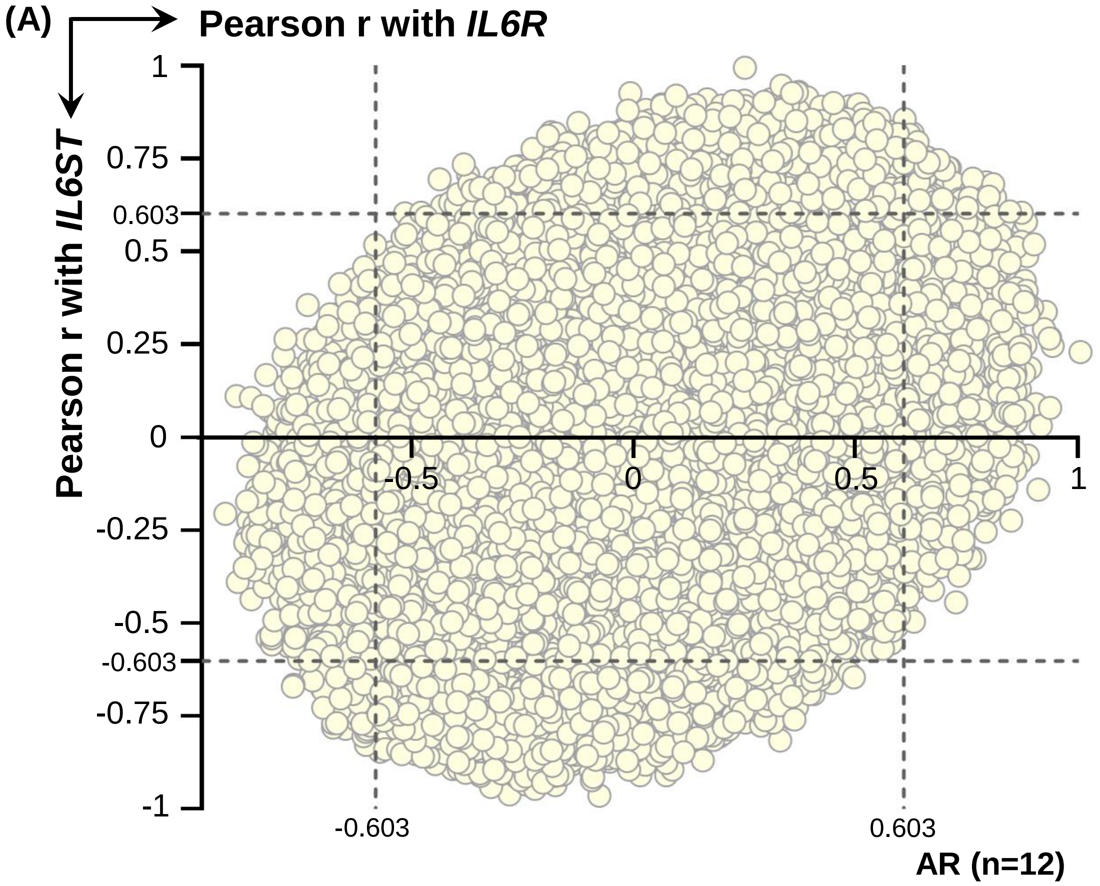

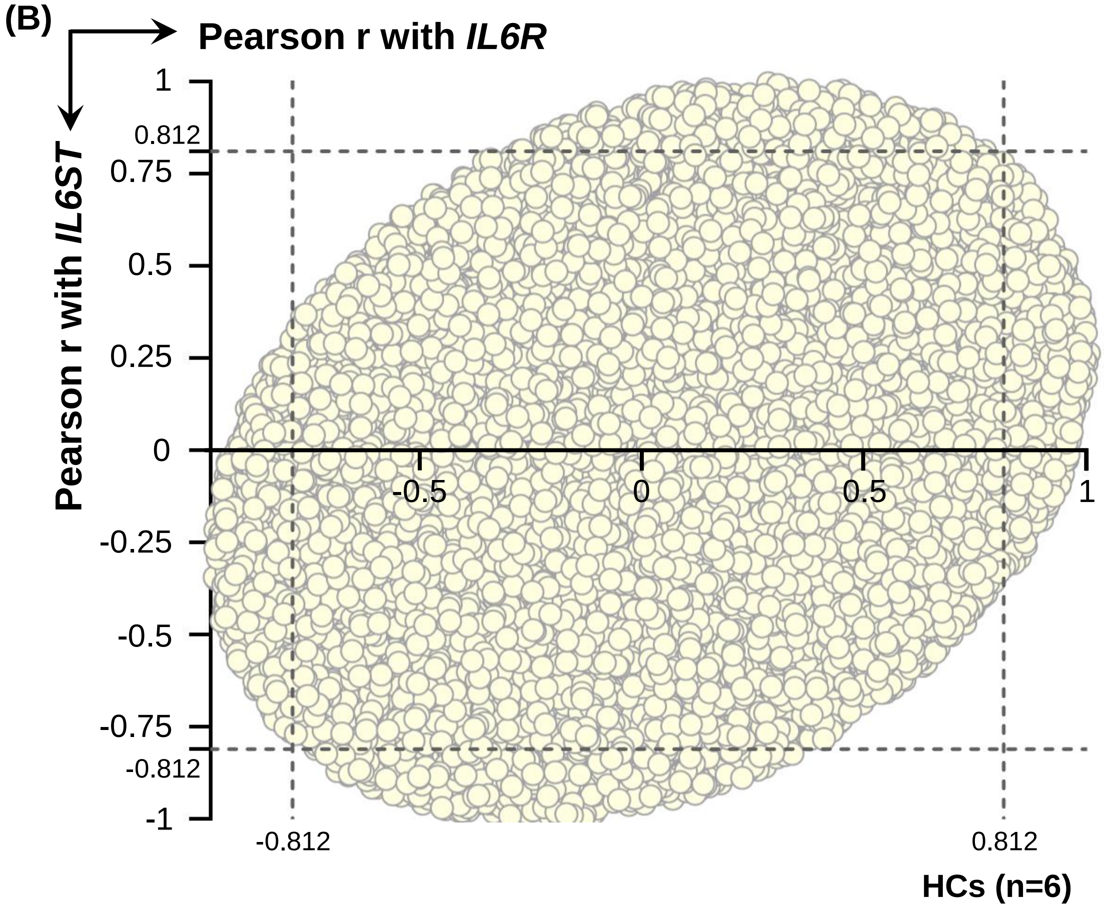

Supplementary Figure S6

(A)

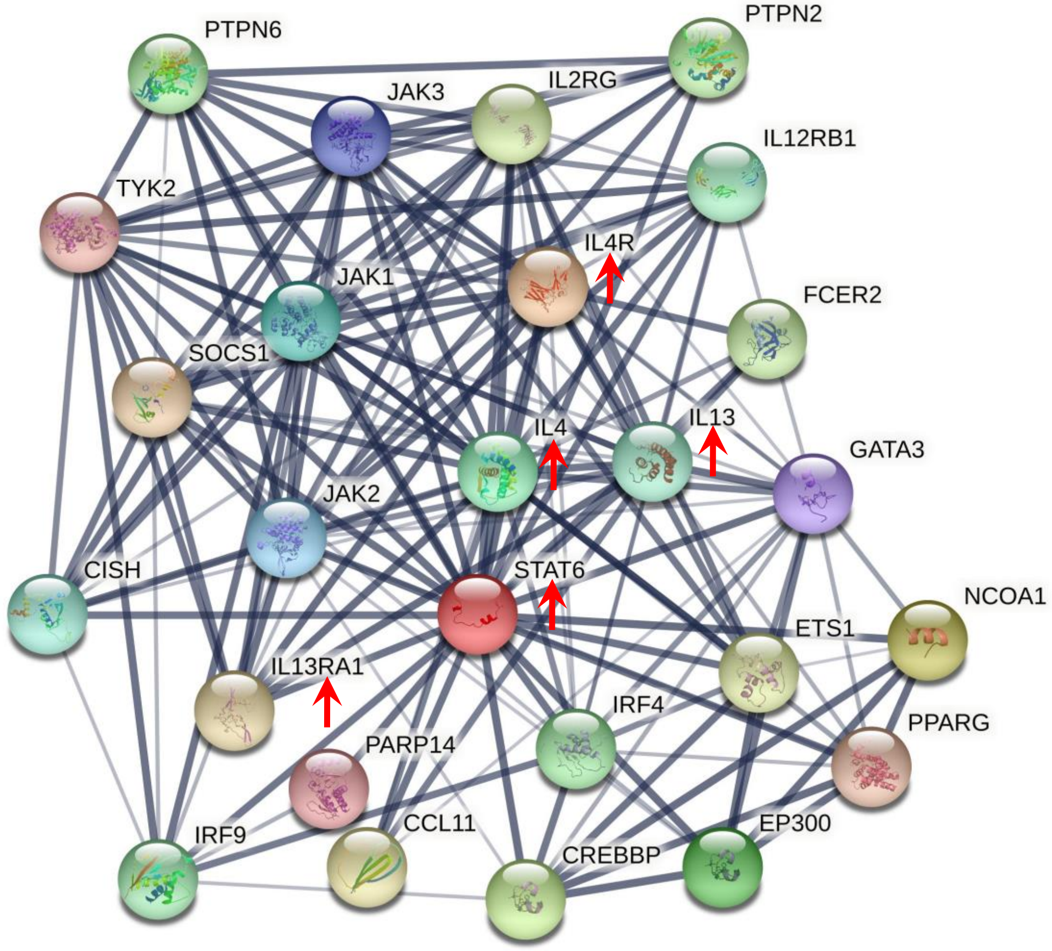

(B)

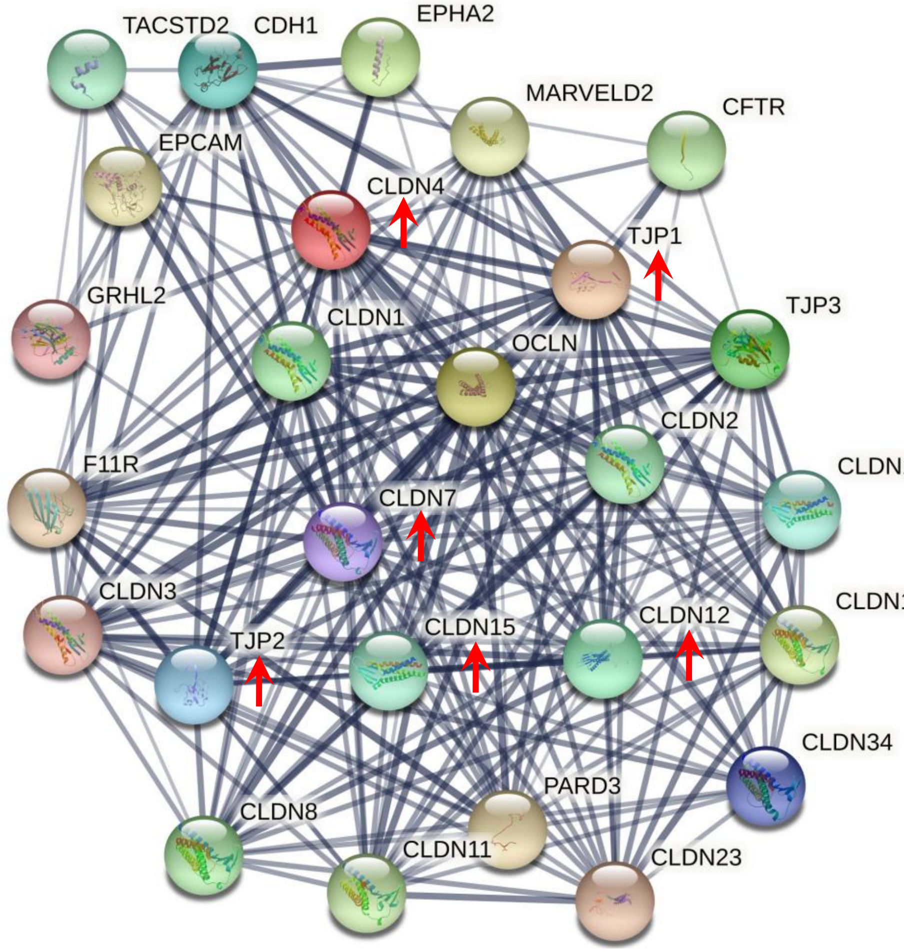

Supplement: Supplementary file 1 [file DataSheet3.pdf]
