# Supplementary material for: IL-4/IL-13 Axis in Allergic Rhinitis: Elevated Serum Cytokines Levels and Inverse Association With Tight Junction Molecules Expression
Source: Front Mol Biosci. 2022 Mar 17;9:819772. doi: 10.3389/fmolb.2022.819772 (PMC8969661; doi:10.3389/fmolb.2022.819772)
Supplement: Supplementary file 2 [file DataSheet1.docx]

**Supplementary Table S1.** Clinico-demographical characteristics of non-allergic controls and AR patients

| **Characteristics** | **Non-allergic controls (n=30)** | **AR patients**  **(n=30)** | ***p*-value** |
| --- | --- | --- | --- |
| Median age (years; IQR)  Gender  Male (%)  Female (%)  Median BMI (kg/m^2^; IQR)  Family history of allergic diseases  Yes (%)  No (%) | 25.50 (26.00-32.50)  8 (26.7)  22 (73.3)  22.86 (20.56-26.81)  0 (0.0)  30 (100.0) | 28.00 (22.00-35.00)  10 (33.3)  20 (66.7)  26.20 (23.47-28.51)  25 (83.3)  5 (16.7) | NS  NS  NS  - |

Abbreviations: AR, allergic rhinitis; BMI, body mass index; IQR, interquartile range; NS, not significant.

**Supplementary Table S2.** List of primers designed for qPCR.

| **Target gene** | **Accession number** | **Forward primer (5’ - 3’)** | **Reverse primer (5’ - 3’)** | **Amplicon size (bp)** | **Primer spans exon junction** |
| --- | --- | --- | --- | --- | --- |
| ***IL4R*** | NM_001257407.2 | AACGACCCGGCAGATTTCAG | GGCTCCCTGTAGGAGTTGTG | 185 | Yes (forward primer) |
| ***IL5RA*** | NM_175726.4 | TGGCAACCATCTGCTTCATCT | CACTGGACCCAGCTTTCTCA | 150 | Yes (forward primer) |
| ***IL6R*** | NM_000565.4 | GGGTTGTGGAATCTTGCAGC | GGAGGTCCTTGACCATCCAT | 194 | Yes (forward primer) |
| ***IL13RA1*** | NM_001560.3 | TTGTTCCAGTCATCGTCGCA | CGTACTTCTTCCAGTGCAGAGT | 156 | Yes (forward primer) |
| ***GAPDH*** | NM_002046.7 | TCGGAGTCAACGGATTTGGT | TTCCCGTTCTCAGCCTTGAC | 181 | Yes (forward primer) |

**Supplementary Table S4.** Gene Ontology (GO) enrichment analysis of genes negatively associated with both *IL4R* and *IL13RA1* expression in AR patients (n=12; GSE44037 dataset).

| **Category** | **ID** | **Functional  group** | **Name** | ***p*-value** | ***q*-value  FDR B&H** | **Hit Count in  Query List** | **Hit Count in  Genome** | **Ratio of hit count in query vs hit count in genome** |
| --- | --- | --- | --- | --- | --- | --- | --- | --- |
| GO: Cellular Component | GO:0140535 | **Protein  complexes** | intracellular protein-containing complex | 6.19 x 10^-11^ | 6.04 x 10^-8^ | 93 | 772 | 12.05 |
| GO: Cellular Component | GO:1990234 |  | transferase complex | 2.04 x 10^-7^ | 9.97 x 10^-5^ | 85 | 806 | 10.55 |
| GO: Cellular Component | GO:1902494 |  | catalytic complex | 3.85 x 10^-7^ | 1.25 x 10^-4^ | 143 | 1592 | 8.98 |
| GO: Cellular Component | GO:0000151 |  | ubiquitin ligase complex | 1.30 x 10^-5^ | 2.11 x 10^-3^ | 38 | 304 | 12.50 |
| GO: Cellular Component | GO:1902493 |  | acetyltransferase complex | 1.30 x 10^-4^ | 9.74 x 10^-3^ | 16 | 94 | 17.02 |
| GO: Cellular Component | GO:0031248 |  | protein acetyltransferase complex | 1.30 x 10^-4^ | 9.74 x 10^-3^ | 16 | 94 | 17.02 |
| GO: Molecular Function | GO:0034212 |  | peptide N-acetyltransferase activity | 3.17 x 10^-4^ | 0.042 | 12 | 61 | 19.67 |
| GO: Molecular Function | GO:0008080 |  | N-acetyltransferase activity | 4.25 x 10^-4^ | 0.046 | 14 | 81 | 17.28 |
| GO: Cellular Component | GO:0005840 | **Ribosome components** | ribosome | 4.72 x 10^-6^ | 1.15 x 10^-3^ | 36 | 269 | 13.38 |
| GO: Cellular Component | GO:1990904 |  | ribonucleoprotein complex | 3.08 x 10^-5^ | 4.28 x 10^-3^ | 74 | 768 | 9.64 |
| GO: Cellular Component | GO:0044391 |  | ribosomal subunit | 5.55 x 10^-5^ | 5.41 x 10^-3^ | 28 | 210 | 13.33 |
| GO: Cellular Component | GO:0022626 |  | cytosolic ribosome | 5.39 x 10^-4^ | 0.029 | 18 | 127 | 14.17 |
| GO: Cellular Component | GO:0005844 |  | polysome | 2.49 x 10^-4^ | 0.015 | 14 | 80 | 17.50 |
| GO: Molecular Function | GO:0000900 | **Transcription &  translation processes** | translation repressor activity, mRNA regulatory element binding | 3.90 x 10^-5^ | 0.017 | 7 | 17 | 41.18 |
| GO: Molecular Function | GO:0030371 |  | translation repressor activity | 4.28 x 10^-5^ | 0.017 | 9 | 29 | 31.03 |
| GO: Molecular Function | GO:0045182 |  | translation regulator activity | 4.17 x 10^-5^ | 0.017 | 23 | 148 | 15.54 |
| GO: Molecular Function | GO:0003712 |  | transcription coregulator activity | 3.63 x 10^-5^ | 0.017 | 57 | 530 | 10.75 |
| GO: Molecular Function | GO:0008135 |  | translation factor activity, RNA binding | 2.12 x 10^-4^ | 0.030 | 15 | 85 | 17.65 |
| GO: Molecular Function | GO:0090079 |  | translation regulator activity, nucleic acid binding | 1.79 x 10^-4^ | 0.030 | 18 | 112 | 16.07 |
| GO: Molecular Function | GO:0003713 |  | transcription coactivator activity | 1.97 x 10^-4^ | 0.030 | 34 | 285 | 11.93 |
| GO: Molecular Function | GO:0003723 |  | RNA binding | 1.88 x 10^-4^ | 0.030 | 161 | 1982 | 8.12 |
| GO: Cellular Component | GO:0016591 |  | RNA polymerase II, holoenzyme | 9.62 x 10^-4^ | 0.045 | 13 | 81 | 16.05 |
| GO: Molecular Function | GO:0140110 |  | transcription regulator activity | 4.93 x 10^-4^ | 0.049 | 158 | 1980 | 7.98 |
| GO: Cellular Component | GO:1903293 | **Phosphatases** | phosphatase complex | 2.04 x 10^-4^ | 0.013 | 11 | 52 | 21.15 |
| GO: Cellular Component | GO:0008287 |  | protein serine/threonine phosphatase complex | 2.04 x 10^-4^ | 0.013 | 11 | 52 | 21.15 |
| GO: Molecular Function | GO:0004864 |  | protein phosphatase inhibitor activity | 8.95 x 10^-5^ | 0.028 | 11 | 46 | 23.91 |
| GO: Molecular Function | GO:0019212 |  | phosphatase inhibitor activity | 1.99 x 10^-4^ | 0.030 | 11 | 50 | 22.00 |
| GO: Molecular Function | GO:0019888 |  | protein phosphatase regulator activity | 1.38 x 10^-4^ | 0.030 | 16 | 91 | 17.58 |
| GO: Molecular Function | GO:0106307 |  | protein threonine phosphatase activity | 4.37 x 10^-4^ | 0.046 | 13 | 72 | 18.06 |
| GO: Molecular Function | GO:0106306 |  | protein serine phosphatase activity | 4.37 x 10^-4^ | 0.046 | 13 | 72 | 18.06 |
| GO: Cellular Component | GO:0070160 | **Tight junction** | tight junction | 1.03 x 10^-3^ | 0.045 | 18 | 134 | 13.43 |
| GO: Cellular Component | GO:0016607 | **Other cellular components** | nuclear speck | 1.27 x 10^-5^ | 0.021 | 48 | 421 | 11.40 |
| GO: Cellular Component | GO:0032587 |  | ruffle membrane | 3.51 x 10^-5^ | 0.043 | 18 | 103 | 17.48 |
| GO: Cellular Component | GO:0016604 |  | nuclear body | 1.22 x 10^-4^ | 9.74 x 10^-3^ | 78 | 856 | 9.11 |
| GO: Cellular Component | GO:0000407 |  | phagophore assembly site | 4.55 x 10^-4^ | 0.026 | 8 | 32 | 25.00 |

**Supplementary Table S5.** Presence of STATs consensus sequence 5’-TTC(N)_2-4_GAA-3’ in the nucleotide sequences of *CLDN4*, *CLDN7*, *CLDN12*, *CLDN15*, *TJP1* and *TJP2* within each gene’s specific DNA regions (5 kb upstream of the first exon, the first two introns and 5 kb downstream of the last exon). Each matching DNA motif is highlighted in green.

| ***CLDN4* (ENST00000435050.1)** |
| --- |
| ***CLDN4*_5’UTR (5 kb upstream of the first exon)** tggtgctgatgatcccggaTTCcttggttttgtccctggctctgtcactgctgactcatgggctgccgacctaggagtctcctggtcagcgtgcgggtTTCtctcagcctcttggtgtgtcacaGAAgcagacagcTTCtctgtaaaccgtcatcctcaggggtgtgcccggcTTCtgggTTCtgtgTTCtggcatcctccgataTTCcaagagGAAgcaggataggacgatcccagctccttgctctgcctactggtgacaagacctgtcctggcctgggaccaggggggcTTCtctgggagtcctggttggtcctgagacagggacaccctcccagtGAAgccctacctcctctgtcctccatcacccagcccacagcaaggccaccctcctgactctccctccaattaacccaagctcctgggttgcgcccttgaggcccacaccgatgctcccaacttactTTCcagccctaTTCcctcacctgtctggcTTCcgactcctcaagttgagttTTCcacagatacTTCctccccacacacacacccgtttttgtccctgctactctccttttgcgagagctctctcctcTTCataggtctctaagcttttactcacccTTCaggtctcctctccacctgccacctcctccagGAAtcctgcctggtgtgccccaggtagagttgatttTTCactctccagtgctcacacagagcttgttTTCcctgcgtgggagctgtttatttaaaTTCaggctggcctgattacaccctgatgagctccatgccagccctgggcctggcccagtgcgctcagcatgtccTTCcaGAAaGAAtGAAgGAAtaaaaaaagacGAAgagtaataaataggagagtaggggtagaggatgagccccaGAAtcagacattaggcagggtgtggataccagtcttgccattaTTCtggtggccctgGAAtgtcacatcagTTCccagagccttagttTTCtcatctctaagatggGAAtgggctaggcatggtcacacctgcaatcccagcactttgGAAggctgaggcaggaggatcacTTCttgaggccaggagTTCgagaccagcctgggcatcatcgggaGAAcctgtctctacaaaaaaataaGAAgttagctgggtgtggtggtgcacacctgtagtcccagctacaccagaggctGAAggtggaggttTTCttgagcccaggagtttgaggcTTCagtgagctacgactgtgccactgcactgtagcctgggtgacagagcgagacTTCatgtcttaaaaacaaaaacaagaGAAcaacaatgatgcccctgcactgttttgcagtgagataaaatgacTTCctgttaTTCaatgttatccGAAggcatttaggttagtgcctggccccGAAgcaggtgctcaaaacatggtttactggctggcttgctccctgagaggccagggcagaggtgcctggcaggaggaggctgccctGAAtggggagagggaggggtggggctggagaggattgactcctccatcagattagagggtcTTCagGAATTCcaccgcccttaggtcctcTTCctccctcgcctctggGAAattggagtaaaatccagtagagacaggactgccctGAAgtgaggaggcgggagccggagggagtcggggaggtgggtggGAAggagggtttatggacctccctgtGAAgtggggGAAtggcctgtGAAgggctggacagctgggtaggatggtggGAAtgctGAAggtcacggtaaatacgggcttgctaattatttatttattagagatgaggtcttgctctgtcacccaggttggagtgcagtggcatgatcatggctcgctgtagcctccaactcctgggtttaagtgatcTTCctgcctcagcctcccGAAtagctgggatttacaggtgtatgcgaccatgcctggctaattaaaaaaaaaaatttgtttttGAAaaagggtctcactatgttgcccaggtaggtcttGAATTCctggcctcaagtaatcctcctgcctcagcctcccaaagtgctgggattacaggcatgagccaccatgcctggccaggtgttttGAAactgacagacataaactGAAatcgatgcaccgatttggggtaaagtaatttaacaacggtcctctaagtcctgtcccctgtgtaagcctcctccccTTCacacagtcatctcTTCtgagtctctctaactaagctctctcaccgagtcccctccctttgctcagcccctctccccatccttgccaccaGAAgtgccagaccctgggactccagccaggaggcatcagtatggtctTTCTTCtcTTCcttaGAAGAAaccccagaggtggccaccgcccccacccacggcccacgtggtgcccaacctgggggcagcacccgctcagccagtctcgccagactggtcagccaggcctgccatcacccagcaccaggccgagggggtggatggtccgctggtgtgggcggtcaggctgagggccagggactctcctgTTCagcctggtgccaggactccaatctgagctcaaggacTTCaGAAaGAAataggatcttgttgcctaaaGAAaGAAaaaaaTTCcatcctttactattttttattttttgagttggagtctcactctgcctcccaggctggagtgcagtggcgtgatctcggctcactgcaagctccgcctcccaggTTCacgctgTTCtcctgcctcagcctcccaagtagctgggactacaggtgcccaccaccatgcccgactaattttttgtattttgagtagagatggggtTTCaccatgttagccaggatggtctcaatctcctgacctcgtgatccgcccacctccgcctcccaaagtgctgggattacaggcgtgagccaccgtgcccggcctactgttttttattttttaagagacaggatcttggtctgtcgctcaggctggagtgcagtggtgtgatctcagctcactgcaacctctgcctcctgggTTCaagcgaTTCtcccacctcagtctcccgagcagctcccgaccacaggcacacgccaccatgcccagctaatttttagtatagacggggtTTCaccatgttggccaggctggtctaGAActcttggcccaagtgatccTTCcacctcagcTTCctaaagtgctgggattacaggtgtgagccactgtgtccgccctagGAAtcaatttaatatacactggcTTCcctggctttagtcTTCctgTTCccaGAAgacgtggagtacgggctctgggagctagactgctcagctcacagagatctttttggctgcaggacagtggtccatcactggccctggggctcagatgccagcgggagcttggggttaGAAgggagactttgtttttgcccctttgctagggactcccagggagctGAActcagggcaagctgcatgtgtgtgtgttacaactggggacttggggccaggcggggtagctcacgcctgcaatcccagcattttgggaggctgaggagggcagatcacttgaggctaGAAgTTCgggaccagcctggccaacatgctGAAaccctgtctctactaaaactacaaaaattagctgggcgtagtggcgagcacctgtagTTCcagctactcgggaggctgaggcaggaGAAtcgcttGAAcctgggaggcagaggttgcagtGAAccgagattgcaccactgcactatagcctgggtgacagcgacaTTCcgtctcaaaaaaagaGAAcaaaacaaaacaaaacaaaacaactggggacttggccaagcccctgGAActctggggacaggggtctGAAaagtcctagctcccacctgttatgatcaggatccGAActgcgcagcagctgctttgcgtacgGAActttgctccctcctcttgttattaaactttttggctcacaaagcatttttacTTCcTTCtctcatTTCatgtgtatctcagactggggagtgggccccagtgTTCtcattgtacaaaggagGAAgttggggctcagagatgGAAttggcTTCtccaaggtcacagggctggtaggatggcttatggatacacagcTTCccagcttttgtcctgagctttttggctcatGAAgcattTTCacatccccatctcatTTCacgtctgtttttgtttttgatcctggagggggtgcaacatcctcatttttacaaaggagGAAgctgtggctcagagatggTTCctgggctgGAAagatggtttatggggtgggcagagatgggtgagctgatctggtggtttttatccttgataGAAaccagcaggtgtggccaggcgcagtggctcacgcctgcaatcccagcactttgggaggccgaggcgggcagatcatctgaggctgGAAgtttgagaccagcctggccaacatgctGAAaccctgtctctactaaaactacaaaaattagctgggcgtagtggtgtgcgcctgtactcccagctactcgggaggttaagccatgaGAAttgcttGAAcccggGAAgtggagactgcagtgagcagagatcgtgttgtctcaaaacaaaaaacaaacaacaaaaacaacaaaaaacaacaacaaaaaacaaaaaaaagcacgtgatccaaTTCtgtcagcacaaaatcacacagtgcatgttataaaGAAGAAaaaGAAaataaatgagtgccaaccctgtactctctgaccccaaaaccaaggtgatGAAtggcagatgtcttataaggtgTTCctgtaaaagatcTTCgggctttggGAAgagggccagatgcaggcctagaggtaaatTTCcttgttTTCctctgccttagggctatgggagctggagcggagaggagaGAAgcaccttgcctttgctggtgtttTTCccgcagcaggtgggtgggactgagccaacggtctcttggcatcaacaaagcatcaacaaatctctctccagctc  ***CLDN4*_Intron 1** gtgagctctgtgcTTCTTCccctgccctgtgtctgctctatgcctggagcctctgtacctgccctctcctcaacccccaacccctccctgctctcagctcccgatacgcgtggctatgcctgggaggGAAggacagcTTCttagggccacaggtggactgttgtagggcttgcatgctcccctcccccatgcctgcTTCtccaagtggcacttggctgtgtcccagGAAagcaagggcagGAAgtggcagggcgggggccgggggccgggggggcaggaggcgggcggggggttggggggggcaggTTCtggccatctggccaagtcacgtgtctactcaGAAagcttgttaaaggctacctggctgggaccacaggtaagaggcacctgggGAAggaggcacccatgctggagtgcccacccgtcctgggcaaggccctggccTTCtctgggcTTCagtgtactcctttgagccaaggcaataaaagtccctgccctgctgactggcaggaggctgtgatgaggtcatctccagGAAagcacttatcgccaatcatgatgttacttatctctggtgttaTTCagcccataggctctgtgtgtagagggagccttgtaaaGAAagccccagggaggcgtggtctgggcaGAAaccatctctgtttgctttggtgtgtgtgcctatgatgggggtcaaggtataggagtgtgatttGAAactggaGAAtgcTTCgTTCtTTCcatcatcaaacatgtctttttttTTCtttttttttag  ***CLDN4*_3’ UTR (5 kb downstream of the last exon)**  agGAAattttattgtctctgatTTCaactgtggaggtgggtgggGAAgggctggaggcctgggtgcgggggtggggggggtcagtcaggggcaggaccactgtgttttggccgggacataaacacttaaaaaaaaaaaaaaaaaacgagatccaggGAAttgtGAAaaattggagctgggccgggtgcggtagtgagcacctgcaatcccagcactttaaaaggtcgaggtgggaggatcgctcgaggcaggagtttgagaccagcctgggGAAcatagtggcaccatctctacaaaaagttaaaaaaattagctgggtgtggtggctcatgcctgtaatccctgtacttggggaggtcgaggtaggcGAAttgcttgagcccaggagtttgaggctgcagtgagctttgatcgcaccactgcactccagcctgggtgacagggcgagaccttatctcttaacGAAaaaaaaaaaaaaatcGAAacgggcctccaaggtatcaccttaaaaaaGAAattaaatgggcccccaaggtatcaccttgtccactgtatatgggttggagggtaaacccatatacaGAAaacagcgtatgcccgggaccccggtGAAgggttggggaccccaggagggacgtccccacgtgggctcccagctccgaggtatgggccacctgccacccccaccccgctgttgttgcagctttgtctgggcagtcaggctggtctgTTCctctttgctgagtcaccctacaggtcccccTTCccagcctttgcacagccggtccctgcTTCcagcctgGAAtgtcccttTTCccTTCtccaccctctggatcctgctcgtcctGAAagcccagctcccagcctcctcccccatGAAgtcTTCccaTTCtcttggatggaGAAattattatcagccctgTTCacatccctgaggtgctgagggtcaccaaatgcctTTCacctggagggcagccTTCaagccacgcccagagctcggcaTTCtgatcccttgatcgatgagGAAaccaagtgtggcccaggcccagacctacagcttgggattttggactccaaatttgggtgggggagctgtccaggacaggacttgggcctcagcaaccacttgggcttggccttggccttgagtctctgccctgctactgttgtgcaaagcatgTTCcaagcttgctcaggcTTCattTTCttgtcagtaagttagaggtgagcggacccagcactctgGAAggagggatccagggaggccccagGAAggggtcTTCtccatcatTTCttgctttttTTCttTTCttTTCtttttttttttgagacagagtcttgctctgttgcccaggctggagtgcagtggtgtgctctgggctcactgcaacctctgcctcccaggctcaagtgaTTCtcctgtctcagcctcccaaatagctggcatgcacccacggcacctggctgatttTTCcaattttttttttttttgagacagagtctcgctctgtcacccagtggcaccatctcagctcactgcaacctccgcctcctgggctcatgcaaTTCtcctgcctcagcctcgcaactagctgggattacaggggtgtgctaccatgcccagctaatttttgtatttttagtagagacagggtTTCgccatgttggccaggctggtctcGAActccagacctcaagtgatcctcccgcctcggcctcccaaagtgttaatttttacaatttTTCtttaagataggggtctcactatgttgcccaggctggttttGAActcgtggtctcaagagatcctcctgccttggccTTCcaaattgttgggattacaggtgtgagccaccacaccctgctacgtctcgaTTCtgGAAcccacagGAAcagatgccactgttTTCtgggagctcatttaataggcagggcatTTCtgaggggcagggttggTTCggaggtcccattttacaggggaggcagaggaggcccagcagatgggcccagctaaggccacatggagtcaggggccagctgggggctgggggctggatctcacTTCctcaacctgggtcgccTTCcactTTCggtacaggtagacaatgccggagatGAAgccatccttggcagagatgccggataccacctccagctcggaggtagccagctcccagcggtccacaggcggtccacaggccaggccaccaggccTTCccacatcccagcctgctccagcctgtccagggtggcctccagagcctccttgtattGAAggttggacgagttggtcctggtggtcagacacaccagcccacctgggggagaggggtaggtgaggtgggggaggggcaggagccacagtcctgggccatctctgctggggatggttgtcccTTCaaggcaaGAAaTTCgacttaaaagattggggacgctctctccctctccctctccctctccctctccctcctctccctcctctccctcctctccctcctctccctcctctccctctccctctccctctccctctccctctccccacggtctccctctccctctctTTCcacggtctccctctcatgctgagccGAAgctggactgtactgctgccatctcggctcactgcaacctccctgcctgactctcctgactcagcctgccgagtgcctgtgattgcagactcgcgccgccacgcctgactggttttggtggagacggggtTTCgctgtgttggccaggccggtctccagcccctaaccgcacgtgatccaccagcctcggcctcctgaggtgccgggattgcagatggagtctcgTTCactcagtgctcaatggtgcccaggctggagtgcagtagcgtgatctcggctcgctacaacctccacctcccagccgcctgccttggcctcccaaagtgccgagattgcagcctctgcccggccgccaccccgtctggGAAgtgaggagcatctctgcctggccgcccatcatctgggatgtgaggagcccctctgcctggctgcccagtctgGAAagtgaggagcgtctccgcccggccgccatccgacctagGAAgtgaggagcacctctgcccggccgccatcacatctagGAAgtgaggagcgtctctgcccggccgcccattgtctgagatgtggggagcgcctctgccccgccgccccgtctgggatgtgaggagcacctctgcccggccgcaaccccgtctgggaggtgaggagcatctctgcccggccgccccgtctgaGAAgtgaggagcccctccgcctggcagccgccccgtctggGAAgtgaggagcccctccgcccggcagccgccccgtctggGAAgtgaggagcgtctccgcccggcagccaccccatccgggagggaggtgggggggtcagccccccgccccaccagccgccccatccgggagggaggtgggggggtcagccccctgcccggccagccgcccatccgggagggaggtaggggggtcagccccccgccaggccagccgccccgtccgggagggaggtgggggcatcagccccccgcccggccagccgccccgtccaggagggaggtgggggggtcagccccccgcccggccagctgccccgtccagtaggtgaggggcgcctctgcccggccgcccctactggGAAgtgaggagcccctctgcccggccagccgcccagtccgggagggaggtgggggggtcagccccccgcccggccagccgccccgtccgggaggtgaggggcgcctctgcccggccacccctactggGAAgtgaggagcccctctgcccggccagccgccccatccgggagggaggtgggggggtcagccccctgcccggccagcccccccatccggGAAgtgaggggcgcctctgcccggccgcccctactgggaggtggggagcccctctgcctggccagccgccccgtccgggagggaggtggggggggtcagccccccgcccggccagcccccccatccggGAAgtgaggggcgcctctgcccggccgcccctactggGAActgaggagcccctctgcccggccaccaccccgtctgggaggtgtgcccaacagctcattgaGAAcgagccaggatgacaatggcggctttgtgGAAtaGAAaggcagGAAaggtgggGAAaagattgaGAAatcggatggttgccgtgtctgtgtaGAAaGAAgtagacatgggagacttTTCattttgTTCtgtactaaGAAaaaTTCTTCtgccttgggatcctgttgatctatgaccttacccccaaccctgtgctctctGAAacatgtgctgtgtccactcagggttGAAtggattaagggcagtgcaagatgtgctttgttaaacagatgcttGAAggcagcatgctcgttaagagtcatcaccactccctaatctcaagtacccagggacacaaacactgcgGAAggccgcagggtcctctgcctagGAAaaccagagacctttgTTCacttgtttagctgctgaccTTCcctccactattgtcctatgaccctgccaaatccccctctgtgaGAAacacccaaGAAttatcaataaaaaataaataaataaataaataaatttaaaaaaaataaaaaaaaataaaaagtcaatcaattaaaaataaataaataaataaataaataaataaataaataaaagattggggacaaataaacactgtaacctaatcccatacatgttactgtgGAAacaaaaagtGAAaataaaacctaattaatgTTCcaataaaaaaaaaaaaaaaaaaGAAaaaGAAaTTCTTCcagcTTCcTTCccagggcagtctTTCtgtgtcttttacattggGAATTCctttTTCtTTCtTTCtttttttttgagaccctgtcttgctctgtcacccaggctggagtgcaatggcatgctcttggctcacTTCagcctccgcctcctgggTTCaagtgaTTCtcctgtctcagcc |
| ***CLDN7*_ENST00000397317.8** |
| ***CLDN7*_5’UTR (5 kb upstream of the first exon)** tcagtctccagagttgctgggactacaggcgcgtgccaccacgcccggctaattttttgtacctttggtagagacggggtTTCaccatgttggccaggctggtctcaaactcctgacctcgtgatccccccgcctcagcctcccaaagtgctgggattacaggcatgagccaacatgcttggtctcccccccttttttttttttgagacagagtcttgctctgttgcccaggctGAAgtacagtgatgcagtctcggctcactgcaacctctgcctcccaggttaaagcgaTTCtcctgcTTCagcctcccgagtagctgggattacaggcccctgccaccacacccagctaattttttgtatTTCtagtagagatggggtTTCaccatgttggctagactggtctcGAActcctgacctcgtgatcctcctgcctctgcctcccaaagggctgcgattacaggcataagccaccacacctggccgcaccattttttttttttaagagacaggatctccctctgttgcccagactggagtgcagtggcatgatcatagctcactgcagcctcGAActtatgggcttaagtgatcTTCtcacctcagcTTCctgagtagctgggactacagacataaccactgtgcctggccatgtctggcacattatagacacttaGAAaatatttgttgagggctgggtgtggtggctcgcacctgtaatcctaGAAttttgggaggccaaggtgggcggatcacctgaggtcaggagTTCgagactagcctggccaacatggtGAAaccccatctctaccaaaaatatttaaaaaattagctgggcatggtggtccgtgcctgtaaccctagctacttggGAAgctgaggcacaaGAAtcgcttGAAcctgagaggcagaggttgtgatgagccaagatcgtgccactgccTTCcagcctgggtgacagagcaagactctgtctcaaaaaaaaaaaaGAAaaaaGAAaaaaGAAaatatttgttGAAcagtGAAtGAAaatattgtttatttgcaatttaTTCaTTCaTTCctcaaatatttattggtgataaaatccagacacatctctaccctcttggagcatacagtatagtggggatattgacattGAAtaaataagtacataaagtaaatacagtattTTCaaactgtgataagagctttGAAagcaaagtactggtagccgtgagagcactatgcagatgatctgactcagtttggggtggggtggtcaggccaggctTTCtaaaaGAAgtgctatttgagggatagctGAAggattagttggttagctggatagagGAAggtgggtgggagtggatggaGAAgagcTTCcagacccagaGAAcagactgtacaaaggcaaTTCagtgggcagGAAcagaGAAggccagtgtggctgaggcagtcaggtcagccatggtgtgacacttactggGAAGAAcacgagtgtcactGAAagattttattttattttatTTCttttttttttttttgagacagtgtcttgctctgtcgcccaggctggagtgcagtggcgcgatctcagctcactgcaagctccgcctcccaggTTCacgccaTTCtcctgcctcagcctcacgaGAAgctgggactacaggtgcccaccaccacacccggctaattttttttttttttttttgtatttttagtagagacggggtTTCaccatggtctcgatctcctgacctcgtgatccgcccgcctcagcctcccaaagtgctgggattacaggcgtgagccaccgcacccggccaactGAAagattttaaacagGAAatgacaaagctctgagtgagtaaGAAgtggtcactccagaGAAtagagtttaaaatgaccagtgtgGAAgggagattggtttggaggcagttgctgtgtccagtcttgggctggtgtggcgtggcggcagtgagtttggagaGAAgctggatatagttaagtgatgacaaatattTTCttttagagctaaaagGAAaattTTCaaGAAaatctcagctggtctGAAggtagtGAAttatTTCaattgattgTTCacagtcagttacagacgGAAcTTCtttTTCcactctcactgctTTCtcactactgcatctgactagtcttttaacataataaaaaaaaaggccgggggtggtggctcaggcctgtaatcccagcactttgggaggccgaggcgggcggatcccgatgtcaggagtttgagaccagtctgaccaacatggtGAAaccccatctctactaaaaatacaaaaattagctgggtgtggtggtgcgcgcctgtaatcccagctatttaagagaccgaggcaggaGAAttgcttGAAcccgggaggcggaggttgcagtgagccgagatcgcaccactgcactccagcctaggcaatagagcaagattacgtctcaaaataaaataaaattaaaaaGAAaaaaaGAAaatctcaccatcgGAAaaaaaaaaaaaaaaaaaaaggtcggggccaggcgcagtggctcacaccggtaatcccagcactttgggaggccGAAgcaggtggatcacctgaggtcaggagTTCgagaccgacctgaccaacatggtGAAaccccatctcaactaaatacaaaaagttagccgggtgtcgtggtacatgcctgtaatcccagctacttgggaggttgaggtaggaGAAttgcttGAActtgggaggcagaggttgcagtgagctgagattgtgccattgcactccagcctgggcgcagtggctcacgcctgtaatctcagcactttgGAAggccaaggcgggcggatcacaaggtcaggagatcgagaccatcctggctaacacggtGAAaccccgcctctactaaaaaaatacaaaaaaattagccgggcgtggtggcgggtgcctgtagtctcagctactcaggaggctgaggcaggaGAAtggcctGAAcccgggaggcggaggttgcagtgagctgagatcgtgccattgcactccagcctgggcgacagaGAAagactccgtctcaaaaaaaaaaaaaaaaaaaaaaaaaaaaaaaaaaaagGAAagtcagttgcggtagctcactcctgtaatcccaccactttgggaggctgaggcagggagatcacttgaggtcagGAAtttgagcccagcctggccaacatggtGAAacctcgtTTCtactaaaaatacaaaaaattagctgggcgtggtggtgcgcgcttataatcccagctactcgggagggtgaggcaggaGAAttgcttGAAcccgggaggcagaggttgcagtgagccgagatcgtgccactgcactccagcttgggtgacagagcgagactctgtTTCaaaaaacaaacaaagccaGAAcaatgatgacacgtggctgtaattgcagctatttgttggggggtgggggggcatgctttgagcccaggagTTCaaggctgcagtgagctataattgtaccactgcactctagcctgggcgacagagtgagacccggcctctaagagtacattaatccaGAAaagtaccattaatccaaaagtacaacaataaaaacGAAtaacaaacagTTCatggctgggagcagtgactcacgcctgtaatcccagcactttgggaggtcgaggtgggttgggatcacctgaggtcagGAATTCgagatctgcctggctaacacggtGAAaccccgtctctactaaaaatacaaaaattagctgggcgtggtggtgggcacctatactcccagctacttgggaggctgaggcaggaGAAtcacttGAAcccaggaggtggaggttgtagtgagccgagatcatgccattgcactccagtctgggcgacaagagcaaaaactctgtTTCaaaaaaaaaaaaaaagagTTCatGAAagtcTTCattttatacaaaagcaggtcaattagcttagcataagggggactttaatatgtttagcGAAtaaacGAAgGAAagacagaGAAatgtttgagagggcaaaacaaagataaaaaacgtgagagagttgaggtaaaaaatggagagagGAAaatGAAatgagagagagccaGAAtaagcagggtatgtgaGAAtctgaGAAgtatgaggaggttggagggtagtagGAAgattgggagGAAactgggcagggtgggtgggGAAagtgcagtgtgcacctaaGAAgagGAAaGAAagattaattgctgatgctgggTTCcaaggagactggagacaaggcgcaaaggtaaacGAAaagacaaggagtgggctgggtgcggtggctcacgcctgtaatcctgcactttaggaggtgggggcagggggattgcTTCagTTCaagtgTTCaagaccagcctgagcaacatggtGAAaacccTTCtctaccaaaaatacaaaaactttagctgggcgtggtggtgtgcacccgcggtcccagctactggggacgctgaggcggggtgatcgcttgagcccaggaggcgaggctgcagtgagccgagattgcacctctgcactccagcctgggtaacacagacTTCctcctctcaaacaacaacaaaaaaaagacaaggagtGAAacagGAAgctccaaagcagGAAaggcagGAAggggagatgaccaaagatGAAggagGAAggagcaaaaggctgGAAggggtgccgggagGAAggagggagcaatgctttggggagagatagcaggactgcagggtgagggaccatggacgaccaccttagagagccagattatagggaggacgGAAcagtgaggcgtgacagagtgcacagcaattggcagtccaagcccaggcccgggaggGAAagaGAAacgggcgagtgtgggggaggggtggGAAaggtggaggataggtctGAAaagactctaagggagggGAAcgatgggtgtggccaagactctgacctagccctcaccctgctccccaccctcggccggcttt  ***CLDN7*_Intron 1** gtaggtggggaggttgagtgcaaagggTTCaggctgtaagtcatgttgggttgGAAtgggggcacagGAAggtggggctgttggggagccacgctaagccgggtgtctgtagcagagccagaGAAccgggacactGAAgagggtgctGAAgggggcgactctcagggatcgagccagggcccccGAAggtgggatcgaccagggtaggagacagGAAaaaaaaggagagcagcgggtgggggcGAAagcagggccgaggagagagcactttggacaGAAcccggcgggGAAagggcggcgccgaggcttgtcaggggcgccccgcagcgtcccaggcgcacctgttggGAAGAAagGAAggggcTTCccggtgTTCgaggGAAatccagtccggaggggctgactcggagcttgggactcctggggagccaccgcctcctccccagcggcggtcaaaaccgggcaagcGAAggggcgtgaccctggtgctcaggtTTCTTCctcctcacctgggcaaggaggggtgggggccacgacTTCcggTTCaggtgagtgtcccTTCggtgacgtcaggtcatcctcggccgcccctccggtcccgcctccccctcccgcgctcccggggcgcgcgggccgcgcccccgacgccctacatatactcaggtgcgccccacctgtccgcccgcacctgctggctcacctccgagccacctctgctgcgcaccgcagcctcggacctacagcccaggatactttgggacttgccggcgctcaGAAacgcgcccagacggcccctccaccttttgtttgcctag  ***CLDN7*_Intron 2** gtaaggccgcgggaccgacggggcggacgcgcccttgaccgaggtggcggggtggcgcgcgtgggagagccgaggccccacggctgggggccctgggttgccggggagctgcagggcgagGAAgtggtcggctgggggcggcggggctgggcgggagcgggccggggTTCcggcggctgcacgtgggcgcgcacctcggtcgcccacgTTCctcTTCctcccccaacccagcctcgtccccggctcggagcagcggtgcttaggccggggccatcccctcctccaTTCccgggcaggccatggcctccgtcatctcccgtccttTTCtcagacaGAAacggccccggggcgcggggcacggggcgcgccgagggccGAAgggcggtgcggctggtcagagtgggccgccgcctgctgcctctaatcttatcggccgattagccGAAgccacaaacccggtggcctcgatagcgggctcagagcagggacgccggctggcatgtgcgccgcgcggggcTTCggaccaccgtctcggcTTCtcTTCgctgcaccctTTCacccTTCcccctatccagggcagtTTCaccGAAaccccggcgccccgggcggatccttgagcggagagggcggggcgggagcagGAAggccgcgctgtcgggaggggcggggacctgcccgcttggcccaggtcttggacacctgggcgcccctggccctcggcaGAAcacggcgccggacaGAAgaggtccagcggttagtgggcgcggccggcggcacGAAggctggagccgcctgcatTTCcagcaatgacggcgccctTTCccctcgcccgcag  ***CLDN7*_3’ UTR (5 kb downstream of the last exon)**  aggcttttTTCccTTCagggcctctgctTTCctcccgtccagatccttgcagggagcttgGAAccttagtgcacctacTTCagTTCaGAAcacttagcaccccactgactccactgacaattgactaaaagatgcaggtgctcgtatctcgacaTTCaTTCccacccccctcttatttaaatagctaccaaagtacTTCttttttaataaaaaaataaagatttttattaggtacgggggtgTTCtactctctcacattaccccatgtcTTCatcctaccTTCtgtcctgatgagacgctgcTTCctggctcacaaggcggggctccatagacaagggGAAccaaggtggggcagggacagggtaaggctaacacaaacaataGAAagatggTTCtgggcccaaagtcTTCccgcgccccctccaattacaatctaatcaaatctggccagtcaaatatccaggtcgtcatctgggtcTTCttggtccaggtcatcataagcatctggctcataGAAgatgtggctggtagcctgccctggcctaggccgcaggagagcctgctgtctgaGAAggGAAgatttggtgctggtgagggtagctaacaggttgggtctggatactTTCTTCcaggtgactgtgactctcaTTCaggcaacctgttaggcctccatgtcattttattggtccaagtccctTTCtctgacctccatgttagagctGAAgcaccatttggtactgggatagatacccttgtactgctctcctaataaaaccgtaagTTCctagcagcttgagggcaaggatcttgtagtcttgcgtcatccgcccacatactgggcacaGAAcagcaagcgtgtactaagcccttgttaaccagcctctgccacgcccatatagccTTCtgtcctcctagcaccactgcctagggatagggccacctcccaagggtctggtctggctcagcccaggcccagggtgctctggggacaactggagctgtgacccaagggtataagggagaGAAaagagacataaaggtcctctgccattataggggGAAtgggtggatcaaGAActtggacttgtctctggaggtggacTTCagtccctataaagtcaggttaatcaataacctgttgccccaacctctccatggcccagatgctgggatctactcagggatccacgtacccaggtcccaaccttacttTTCaGAActGAActgGAAgggcaggatcaggctatctctggcTTCtctctctTTCttggacaggtGAAggttaaaggtcaaatgagttgtgggatccacctgtaggtacaaaggatgctgccactctgccccatccctaGAAtccctgaccccacccccaacctctgcagcttttattTTCtctcccgtgaccagatactaaactacatTTCcttgTTCtggccttggcaTTCttagataccgggggtatatgaggatcggagtagggctgggactctacagagggcccctcttggagatccaggctGAAgtccgGAAggatggaGAAccactgagtctggagaGAAaaaaGAAaagagtttttTTCatcttactGAAGAAactagacagactccaggacctcacatcctcagcccccaatcacctatttaactgactgctTTCagctcctgccctcaacttttttttttttttttgagacagggtctggctttgttgcccaggctggagtacagtggcgtgatcttagctcactgcaacctccgcctcccaggTTCaagtgaTTCtcctgcctcagcctcccgagtagctgggattacaggcacgtgctaccacacccagctaatttttgtctttttagtagagatggggttttgctatattggccaggctggtctcaaaccactaacctcaggtgatctgcccacTTCggcctcccaaaatgctgggattacaggtgtaagccactgcgcccagcctctattttattTTCaaaagtGAAagcaagttgattaagGAAgtaaagGAAtaaaaggatggcgactccttaggcagagcagcccacttactccttttttgagacggagtctcactctgttgcccaggctggagtgtagtggcatgatctcggctcacctcaacctccgcctcccaggTTCaagtgaTTCtcccacctcagcctcccGAAtagctgggactacaggcacccaccactatgcccagctaatttttgtatttttagtagagatgagatTTCaccatgttagccaggccggtctcGAActcctgacctcacaatctgcccacctcggccccccaaagtgctgggattatgggcctgagccacctcgcctgggccccacttacactttataaccttggtaagtgacttaaatttaaacctccttgggccgggtgcagtggatcacacctgtaatcccagcactttgggagaccaaggccagaggatggcttgagcctagttaTTCaagaccagcctgggcaacagggtgagatctcatctgtataaaaaactaaaaagctggctggatgtggtgctacatgcctgtgctcccacctccagctacaccagaggctgaggcaggaggatcacttGAAcatgggaggtcaaagctgccgtaagttatgattgcaccacagcactccagcctgggcaacagtgagaccttgtccaataaaaaaaaaaaaaaaaactccaaggGAAtaaaaccctccctggactggctTTCcagcctGAAaaatggtgataataaaacaatctaccttaaccatagctgagaggattaaatGAAgtgagtgataGAAaacagaGAAatacctaaatcagctttttttaaaaaaaggtctcactgagggctgggcacagtggctcacgtctgtaatcctagcactttgggaggctgaggtgGAAggatcacttgagcccaGAAgtttgagaccaccctgaccaacacagtGAAaccccatctctaaaaaagtttgtttttaaagtctccttGAAacatcagcattTTCattgTTCTTCccatcgtgccacccctTTCtctgtcccccgtTTCcagtccTTCtctgttggTTCTTCtgacctggtcagttgggcgctgtcggggcctccgacacaggatgtgggccgaggcctggcccatggtaccgcccagggtcacctcagtctgagcaaggctgctgagagctcccacagggcctggtccatGAAgctcTTCatgtagcaagcccagcacactcactTTCcccactgaggagctgtcacctagaggaggacaaGAAatgtaggatagtagggagcaTTCaggttggtggcctgggagctaacagtGAAGAAgagagagcctcacaaTTCcatcacagtgctgccaaaatcctgacgtcccttggccaatctcactaccgtgctctgagcctcctccagcccacaccttaatctcctctatcatcccaatgtaaagctgatcaaggcaccgctctaacTTCtaTTCTTCagcagctcctgccTTCaggatgGAAcGAAcgcTTCcagcaggtggtgtccacttactTTCcagcctcatctcttacacacgGAAgtacttgtagtTTCtgGAAcTTCccaagatctcagtcagtgccTTCaaTTCtctgcctccctttttgggagcatttagagagtgtcaggtactatgctaaacgctttaacacaatgtttGAAtccttacaaaggtcagcgtttttaggtcaaactgtatGAAatcactgacatttaacaatttttttTTCttTTCtttTTCgagatggagtcttgctctgtcgcccaggctagagtgcagtggcgcaatcttggcttactgcaacccctgctacgggctcaacaatactgcctcagtctcctgagtagctgggactacaggcgcgtgccaccacgcctggctaatttttgtatttggtagagacggggtTTCagcatcttggccaggctggtcttGAActcctgacctcgtgatctacctgtcttggcctcccaaaatgctgggattacaggcatgagcctctgcgcccggactgtttttatttttttgagatgacgtctTTCtctgtcacccaggctgcagtacggtggcgtgatctcagctcactgcaacctctgcctcctgggTTCaagtgaTTCtcctgcctcagcctcccgagtagctgggattacaggcgtgcaccaccacacccagctaattttttgtatttttagtagagacagggtTTCaccatgttggccaggctggtctccaactccaggtgatccgcccgccttggcctccccaagtgctgggattacaggcatgagccactgcgcccagacaatatttaacaacttttaacctaaGAAatggcaatatagtggTTCatcctaatacTTCccttgtaccTTCccccatatacagggGAAagtGAAgcaatgagagatcaagcaccttaatacaaagctgggcgtctgactcctaagtccaTTCgagtacccacagtgcacTTCaaggctagtctcagtgcccctgcccggccaaatttgcctggatgacctcttgctcTTCaagagagtgcctttttttttttttgagatggagtcttgctctgtcgcccaggctggagtgcagtggcccatctcagctcactgcaagctccacctcccgggTTCacgccaTTCtcctgcctcagcctcccgggtagctgggactagaggtgcccgccaccaagcccggctaatttttttttgtatttttagtagagacggggtTTCaccatgttagccaggatggtctcgatctcctgaccttgtgatctacccacctcggcctcccaaagtgctaggattacaggagtgagccaccgcgcccggccaagagtgcccTTCtttaaggtgccctgtgcatgcctgtatcatcatggcctttatcagaccatcttgtaactgcctgtttacttatctccatcttttatgggacTTC |
| ***CLDN12* (ENST00000287916.8)** |
| ***CLDN12*_5’ UTR (5 kb upstream of the first exon)** GAATTCtactcaacatTTCaaGAActaacaccaaTTCtactaaaactcTTCaaaaagattGAAgacgagaGAAaacatccaaacccattttatGAAgccaGAAttaccatgataccaaaaccagacaaggacaGAAtGAAacaaGAAaactataggccgatatcactaatGAAcatacatgtaaaaaTTCtcaacaaattttgagatactagcaagccaaaTTCaacaatatgttaaagactgtgtgcagtggctcacgcctgtaatcccagcactttgggaggctgaggcagGAAgaccgcttgaggtcaggagTTCaagactagcctggccaacatggcGAAaacccatctctactaaaatacaaaaatcagatgtgcatggtggcgcatgcctgtaatcccagctactcaggaggctaaggcaggaGAAccgcttGAAccagggaggtggaggttgcaatgagccaagatcatgccactgcactccagcctggatgacagagcaagactctTTCtcaaacaaaacattataaagatcaTTCaccataatcaagtgagaTTCattacagggatgcaaggatggTTCaacatatgcaaatcaataaatgtgatatatcacattaacaGAAccaaGAAcaaaaacaatatgattatttaaatagatgctaaacaagcacttgataaaatccacatcgctttttgataaaaaaaaaaccctcatcaaattgggtataGAAgGAAcatacctcaaaataataaaggccatctatgacaaacccatagctaacattatactGAAcaggGAAatactGAAggccttTTCtctaagatctaGAAcatgacaaggaTTCccactTTCaccacttttaTTCaatatagtactgGAAgtcctagccagagcaattacgtaaaaGAAaGAAagagcatctaaattgGAAagGAAGAAgtcGAAttagccttgtttacagatgacatgatgttttacttaGAAaaatccaccaaaaaactattaGAActgataaacaaatttagtaaagatgcaagatacaaaattaacatacaaaaatcagtagcatgtatatatgccaaaagcaaacaattGAAaaataaatcaaGAAtGAAattttatttataatagctccaaaGAAtataaaacacctagcaattaatttaaccaaagatgtGAAagatccacacaagGAAgactataaaacactgatGAAaGAAattGAAgaggacacacaaaaaatgGAAagataTTCcatgTTCatggattGAAaaattaatattgtgagcatgacactaaccttggctattgacagaTTCagtgcaatccctTTCaaaatactaatgacatttTTCacaGAAattttaaaaaaTTCtaaaatatatatGAAaccacGAAagaccctaaatagccaaagcaatcctgagcaaaaagaccgcagctggagatatcacactacctgacTTCaaaatttactataaaggcatagtaatcaacacagcatggtactggcttaaaaaacagacacagaggccaatgGAAcaGAAtaaaGAAtgcagatataaacccatgcatttacagccagTTCatctttgacaaaggcaccaaGAAcatacaatgggGAAaggacagtctcTTCaataaatgatgccaGAAaattggatatccatatgttGAAGAAtGAAactagacctctatctctcacaatacacaaaaatcaaatataaatggattaaagacttaaacctaaaacctGAAactatGAAactactaGAAGAAaacattggaGAAaccctccaggacattgtctgggcaaggattttttgtgtaagaccttaaaagcacaagcaatGAAagcaaaaataaataggattatatcaagctaaaaagctctgcacagGAAaaGAAacaatcaacaaagtGAAgagacaacacacaGAAtggGAAaaaatatttgtgatctacctatctgacaagggattaataacgaGAAcatataaggagctcaaacaatagcaaacacacatacacacacacaaataacctgattaaaatatgggcaaaagatctGAAtagacatTTCtcaaaaGAAgacatgcaaatggccaacaggtatatGAAaaaatgctcaacatcactactcattagaGAAatgcaaatcaaaactacaatatccctccccagttaaaatgacttttatctaaaaggcaataatagatgctggcaaggatgaggcaaaaggGAAactcttgtacactgttgatggggatgcaaattagtacagccattgtggaGAAtagtatagaggtTTCcaaaaaacctaaaaatagtactaccatataatccagcagtTTCactactgggtgtatatccaaaaGAAagGAAatcaatatattgGAAaaacaatacacttatgtatattgtagcactaTTCacaatggtcaaaatatGAAatcaacctaaatgcccatcagtggatGAAtggataaaGAAaatgtgctatatttacacgatgGAAcattaTTCaactattacaaaaGAAtGAAatcctgtcttttgcagcaacatggatgGAActaGAAgtcattatgttacgtGAAataagccaagcacaGAAagacaaataacacatgttTTCactcatgtgggacataaaaaagtGAAtctcatGAAaatagagagtaggctggtggtcatcagagaccggGAAgggtagggtgGAAgaggggtttGAAaaGAAgttgattgatgggtacaaatatatggtttgataGAAGAActgaGAActaatgttatattagtatggtgactatagtttacagtaatgtgttgtatatctcaaaatagctaGAAgaGAATTCaaatggttttagcataaagcaaagacaaatatttaaggtgatGAAtatcccaagtacactgatttgatctttacaaattatatGAAtgtattatatgatcatatctacTTCtaaacactatGAAcatctatcacacattagtaaaaaagggGAAaaacccctgctgacatcctgattttagcacagtgagacctgtgtcagatttttgacctccaGAAttgtaagataataaatttattgtgttttaagcctttGAAtttgtggtaatttgtcacagcagcaataGAAatctaacacagtgaccaaatggGAAtctctgtaGAAttttggtggtGAAtggtacactgcagtGAAtttaagatttgactTTCtccttGAAGAAtctctaatgaGAAaaagagGAAagacaggattgttaattgcagatctaaaTTCttatctaaagtatttttgaGAAttataTTCcaattatctaactattGAActccaacagggtatttaagtaTTCtgTTCcatctttgctatgccagaggcaggcattttagaccccagcttGAAcagtaagtctagGAAtagggttgtctTTCcTTCctttttgtgctgtgattttttaaaattattgtaatcaattgcaagtacctcccagctaaagtcctctaatcaggcagtaTTCatcagtgattggGAAataagttataagatgattttgtacatagttatttgaTTCaaTTCagctttatagatgagaGAActGAAgTTCagagGAAataagtgattatcgtttgtcacattgctattatccatctgctattgccagagctggcctggagcccaGAAatcttTTCaccacccaatgctatctTTCTTCctcattattacatTTCccagtctggctttgggatcggattgcTTCtggccttagataaccttatccatcctctaaagcagtatcacacataaGAAggaggtagcagtggactgatagtagcctctctaccctccctttttaaaaagtctctgacaaatagtctcattgttactagagcaacgcaaaatgGAAaatgctaatagcaaacatatagtccttattatgcgtcagatactgttgtaactgtctctgtagtaaTTCatttaatcctcatcacaattTTCtgatgtagatatactgttacagatgagaccttgaggcacaaaggggttaccttacttgccaaggttatacttacttgggatgggtgccatccttttgtataaactatgatactttTTCTTCtttttattttGAAaaatTTCtaagatacagaGAAtttGAAaGAAtagtacaagagacacccacaaacataTTCTTCatGAAtccaacaattatacactttgccacactttatatccatttgtgtgtgtgtatgtgtgtgtgtatgtgtgatcatTTCGAAgtagtaggcatcacaacacTTCagcatcctttattttttaaaaaatcagatcattgctatttaggactttgGAAtcttttagtttaaTTCagataTTCaTTCactaatgcaggacTTCacattggagaGAAgccaacagaGAAagggacaggacagtaGAAattgtgtgggagagtaaaattagTTCaaatatctaatagctagctatactgtactttagctatattaggtccctcagttTTCaagacaacagtatgatgtgggtgatcttatccccatttTTCcagataacctccagatgtagagataactTTCccagggccacccaatcaatacacagcaaagccccatacaaatctgggctgtttggTTCtagactttgagctctttTTCtactttttgcctagataaacactttgagggGAAaaaaaatccagatttaaGAAtttttgctaaccTTCctacgggcaagtcaccTTCaagtcacctgggtacttaggtatccaactcaaaactacttttagttttgtTTCgttttgccttttttttTTCtttaaccgataacccaaTTCtagacgGAAcataaggtgcaagaGAAatccggggaggtggaggcagaGAAaggGAAgggcaggaggtggtcgcaggatgttgcctgcggctggcggcccagtggaTTCtggGAAttgtagtccca  ***CLDN12*_Intron 1** gtaggtgtggtcaagatgTTCcgatTTCtgggtcttttgcataagtaactgaggattttatgtaaaatggctaaatccaggaGAAtcaggagcgttgGAAaacgctgaccacatctgtggatgtGAAaattgagtaggtatttggtaaggctttaaaaaattattttgtacacaggagtatgGAAatgtcgaggtgtgttgccggagccgccaaggactgggagctttgctggcgcgggtttgtgggggagcccaagctgctatttttGAAtaaccagctgatgctggttgcgtaatgatgacctgcttgcggTTCaaacccactcagtgttgcagggatgggctagctctgagagcgttaacagtcactgttgcggtctctctccattggctctaactagtagggcgttTTCTTCcTTCtgccaggtctgggtctaccacacacagggctcTTCGAAaagagggaggaggagaggaggcggaggggggaggaggggggcggagggggatgcggattgctctgGAAgcttggtttgctTTCctgtaagTTCtgtgcacccggccttgtaaggggcccatagccacgttaggGAAaggtcagacttggtgacctcacttaaaatgattgctcaaggaTTCtattgtgcgttattaattgtaaaacgatTTCctctgactttggagtgtgcaattatgattgataccaacccaaGAAacacatttggggcgggggaggggttgggggggacttgactGAAatctccgagcagccagttgcagagagcaTTCtacataatgatcgtaatagggctagtgatGAAcgcctcaatccagGAAactctcaggcgcacataataatgagtTTCcaTTCtcatcctacctgtttgagataaatggtatttGAAgagGAAgtggctaatgtttaaagtagatatataGAAgcatctttaagtatcaggttgagccattggtgGAActattttttttttaagcaTTCtggttgcaaatTTCaggatgcaGAATTCagatcaactttgtctggtcttgttgtcagtgtccttttttaaaaatgtaaaaGAAaatTTCttttacTTCcaagacaactgcacagtctTTCaccaccTTCcttatcctTTCaccgtagtcatGAAaatgatctaaaacaTTCattaaatatcaatcatataTTCccaagacagcaaagtgtttTTCatcctcatTTCtcctataTTCtcaagtcacaagatgagttaatttttTTCTTCtatTTCtacgtatttTTCtataTTCtatatatTTCtcatatataatgcatGAAttaagGAAtaaacagcctttttatttttttttttttattatttgagacagatcattgctatgtcatccaggctggagtgcagtggcatgatcatgactcactgtgtcctcaaactcctgggctcaagtgatccTTCcatgtcagcctcccaaGAAactgggacaacacgtgtgcaccatcacacctggctaattattattattattattttttgagccaGAAtctctctctgtcaccaacactggagtgcagtggtgcaatctcagctcacggtaacctccacctcccaggTTCaagcggTTCttgtgccttaacctcccacatggctgggactataggcatgagccaccatgcccagctaatttttgtatttttaggagagatggggttttgccatgttggccaggctggtcttGAActcgggctcaagtgatcatcccgcctcagcTTCccaaagtgttgggattacaggtgtaagccactgtgcccctgctaaactgcctttttaaaaaacttactgtctgccctttttagtataacatTTCattggttttaggtgattactaatcatTTCTTCtaatccatggGAATTCaagcagggtttttagaGAAgtactatgttaatttggagtatttTTCctatTTCtttag  ***CLDN12*_Intron 2**  gtaagtacgtcctttaattatatcttatTTCcctccctcctgctaccacatctgccaacaatgactctttttttTTCtcttgTTCaacaaaatagaggtataggacagacctctcttgtgagtacaatgactgatgacttacagtTTCtaaGAAaacccaaattataggtaGAAgGAActctcaatgttaccatcaaGAAttaagttagggctgggcatggtggctcagccctgtaaccctagcgctttgagaggctgaggtgGAAggatcccttgagcccaggagtttgagaccatcctgggcaacatagcgagatcctgactgtatgaGAAaattttaaaaaattagccagttgtggtggtacatgcctatagtcccagctactctgGAAgctgaggtgggaggattgcttgagcctcggaggtcgaggctgcagtgagccatgattgcaccactgtgctccagcctgggtgaGAAagcgagaccctgtctcaaaaaaataGAAgctagctgctattagacatatgtactctcgtaactcttgtaactataactcaGAAttgtgcctgGAAaaaatagtacagggtctattggatacatgacTTCctaaagctcttagccactgTTCctgctccagccTTCtttaaaTTCtaTTCaaaaccacaGAAaatatataatttGAAaatatttttaTTCgtaattatgtctctgataacatttaattgtgtatcaaattttatctacttTTCtgagtcacggtgGAAcagagagtcaaatgacaaatgtGAAgtggttatttGAAatagagtctctatttgatttggtcagtatctccaaagaGAAtgTTCtgtccTTCttgttatatgactataaccatctgtagtcatttgctgagggtccaGAAGAAaagtatgtTTCtagctGAAaaaagtaaataagcctgctaataatcattactgacttacatTTCtgaccataTTCtggtttacaaaggactttgctaTTCatgattttactactttgtgggacactttacagatGAAaaattggaggTTCagaggacataagtgacttgtccagtttgtaaatgataggttatGAAtgttaTTCaggtctttggtctcctggacaacaataccaagcagcctcctggatgGAAacaacaaaccacttatgtgtatgtatgacctagctcaaagctctcatccagctagtactcatgactgcttgacagatttgttaaagtgTTCttagccttgcacgttTTCcacagtccctaattagggattTTCacatggcaaaaagatgtttttttTTCtccattaaaaaaactatatattgtttagctttaataaatattaatttgatatattaaccactcttaagaGAAgtatgtctaaTTCatgacatacctgttaagttGAAaacaGAAaaatcaataccgGAAcagtggactttttgtttttGAAatggagtctcggggtTTCaccatattggccaggctagtctcaaactcctgacctcgtgatccacccacctcggcctcccaaagtgctgggattacaggcatgagccaccacgccgcgccagGAAtaattgactTTCaaaccatttgttGAAgttagacagtgttgctaaacctagatatatatTTCtaGAAtcatagtctgcatgtaggtacctaaggatggccttgtctttgctaatcacaggtTTCtggggtaaGAAaatGAAtgttgttttgtaaaatgggagtcttttggccaaaaaggaGAAagcaggggcacagtctaGAAacagtctggatgtatcaGAAcacagggtggtgtGAATTCtctcaaatGAAgtGAAtgGAActgagactaaatTTCtgtctgtaTTCtgaccagagttatacaGAAcctccatttgtttgTTCaTTCaTTCagcaaataatcgagtgccttttatgtgccagtataggGAAcagtGAAcaaaacaaagggatctctgtccctgGAATTCacagtctgatggGAAaGAAaactacactGAAcaaggagttataaGAAtgatcactgctacaGAAGAAaaagtgcaaaagtgctaccacagcatataaccagggacagggggaggtttaggacaagcctccctggacactaGAAGAAtgagtaaatattagccaagcaaaGAAtaggttgGAAttaaGAAagTTCtaggaGAAagaccatgaggGAAggcccttaaccaaatcaGAAagtgGAAaatttggGAAgtcaaaaGAAatgtggtttggctgcatagactgtagtgtgtggggtgggagagtggcacatacGAAggttgaGAAtttggtagGAAtcaggtggtgtaagctatggggaggagttGAAactttagagattttaTTCtgtggGAAatggGAActtattGAAtggtaaGAAatgGAAtggcggatgaggtttgcatttGAAaaaaattattgtggattaaaactaattgacacagcacaagagtagaggtctttttacccttgcaagtatGAAcaaatcctaatcggGAAgtcagcattatctttgttttaaaacatcaaacataTTCtTTCcccaaactatcctataaaattttacTTCaggcatggtggcttacttttgtaatcccagccctttGAAaggtggaggtggggggattgcttgagcccaagagTTCaagaccagcctgggcaacacagcaagaccccatctctaggGAAaacttaaaaattagtctggcatggtggcatgcacctgttatcccagctactcaggaggctgaggcaggaggattacttgagcccaggatctcagctcactGAAatctagtgagctgtgatcatgccctgcactccagcttgggtgatagagtaagaccctgtcttaaaaGAAattactttatctctctggtgggatatatgacttatcaaatcataggcatttGAAattaaGAAcagggcatacaaggtGAAgtagccaaaaGAAgctagacattaaTTCcagtatcaggccaaaatttagcttaaGAAttaaggttaatagtacTTCccTTCtGAAaaagtgccgtggtatccttgttgactctgtatagataggcctctgtccatccctgagatctgagctaaGAAtgcTTCatcaaaggcaatTTCagaGAAccaatataatatttgtattgatttgatgcaGAAtatagttaaaagcacttactactGAAatatatattgtcgtacctctcTTCattaatgtcatttgatagtctataggctaaGAAttatagGAAtttatttatttTTCttaataattaatttTTCttttttttgtagagatggggtctcgctatgttgcccaatctggtctcaaactcctgggctcaagctatcctcccaccgcagcctccccaagtgctgGAAttacaggcatgagccacagcacctggcctgGAAtttattaataatggagttatacatcctgatcttgtTTCtcactccatttacatTTCagGAAtcagtgttacagggcagtttgattggaGAAatctcaattacattGAAaTTCcaattatTTCtatactacttttgttttttgTTCctgtttgtttgtttgtttgtttgttttgagacagagtctcgtgctctgtcacccagactggagtgcagtggtgtgatctggctcactgcaacctccacctcctgggTTCaagcgaTTCtcctgcctcagcctcttgagtagcagggattacaggcacgtgccaccatgcctggctagtttttgtatttttagtagagatggggtTTCaccatgttgaccaggctggtctGAAactcctgacctcaagtgatctaccagccttggcctcccaaagtgctgggatTTCaggcatgagccaaTTCgcccagccagtatattacTTCtGAAagcaaGAAgGAAttgtgattgcattaggactttgagtgggttatctGAAgacTTCtGAAgcttGAAaGAAacgaggGAAcctttTTCctatgtcTTCacaactttaatcaaatccttaTTCtccTTCatTTCtcagtctttaactcctgcctgataaatttttatGAAcaggtaccttaTTCactttgcttaacacctaacaataatcacttgatacgtagtaattatacagttgaTTCaccaccaacactttgagtttacataagacagccgtTTCtTTCcacaagtctctataaacacattgggagcaaggtccagttatcagtggtcttggtgtctTTCcagggcctaacagagtgttttgcatatgctagacactcagcatgatgtaagtacttgctGAAtGAAtgattgcTTCttagtaatagctacTTCcttgtacagTTCTTCattaccagtggcccacctttgtgagttactggggtctttgtacagagcccaatactcgtttaTTCaaataatcctaGAAtcacttgtgtgccaggcacacaagttgtatgggggagatagacagatGAAtaagcaaagaccaccagagagctgtatcctagagcttggcatTTCtagtagctttTTCcagtgcaaacaTTCtcaaatctgtactagtTTCcattgctgtactgaggtgtttggttgccacaattaagcaagacagctttTTCccaatttgcttGAAaaaaatactgtaattagtctctccctTTCataaaattTTCttaaGAAattgctacTTCtgataatactgacaGAAggtaacataataaatactaaactaatactaatgGAAaaacttaGAActaaccatgtttaacttgagggctttaaaaatggttacttgtgcttaaactctgatttttatattgtacatttatatatgctatttgtgtatatttGAAtatatgtatgtacacataggcatttttttggttagtaaaattaTTCtggctgggcgcagttgctcatgactataatcccagcttgtaatTTCagcacttttggaggccaaggcagcaagattgcttgaggccaggagTTCgagaccagcctggccaacatggtGAAaccatgtctctaaaaGAAaGAAaaGAAGAAaggGAAtaaGAAttgtgggggtgGAAggaggagggctggTTCacacctgtaatctcagtactttgggaggctgacgctggtggattgcttgagcccaggagTTCgagaccagcctaggcaacatgaGAAaaccccgtctctacaaaaaatacaaaaattagctGAAcatagtgacgtgtgcttatggtcccagctactcaggaggctgaggtgggaggatcgcttgagcctgGAAggtcaaggctgcagtgagccatgattgtgtcattgcactctagcctgggcaacagagtaagacttgtTTCaaaaaaaaaaGAAaGAAaaaGAAaaaattaTTCctatattatatgactagattaaatatacaaataacattgttTTCtaaaGAAcattgtgccctcccactgGAATTCatgcaacaatcacaTTCtaagccagcTTCctagcattttggtcataattgtctTTCtgatcctGAAagcttgGAAacacaacTTCaccatcccagcagatgtttgaTTCtgcaaaggttgatgGAActgtaTTCaGAAataaaaattaTTCtcaaccttgccccTTCatcatgctctGAAcaaaaaagtaaaccttactGAAgtagtctgctctattgcctcatagtaggcattataGAAggcagaGAAaatacatttgagtcctgctttgagagtGAAtatcagacccttgagGAAattaaagctGAAtaagatgcaagctttttatagtttgccatatacaGAAaaaGAAcatggccttttaatttgGAAtGAAaggcctggcgactgtttTTCttataatagcattaataGAAataggttgtcgttacttGAAaaatgtttgcaTTCgtcttatatGAAcagtagtagtaataaccaacaagtgctgtagatTTCcctGAAaatctacatcttGAAtcaacaagGAAgtaattgggtcatgttGAAtcaacaagGAAgtaattaagcaGAAtaatTTCagggaccattgTTCtcatcttTTCatttgactgactcattggagatgagctcatttactttttataatTTCctagtgtgcctagtacatagGAAtgagatcagtttgttttTTCttatttaacaTTCaTTCaatGAAtgTTCtttGAActcctgtgatgtgccaagcagtgagctgggtcactGAAtgctaaGAAgtagttaacacttggcatagcatttgttgtgTTCtagacatgttttaagcacttgacattatattatctcattGAAcccataGAAcaaccctgcaaggttggcactgttattttTTCcatTTCacagatGAAGAAaacgaggcacaggGAAgTTCtGAAtctggcccaaggtcagtgagctagtaagtgaggagatggggattaGAATTCaagcaaccttgctaagagttgactctcaaccaTTCccctgtcctgtactgcTTCtTTCcctacacctttgctttttatgagccaccTTCacccctgctgcatgagcatttgaggctGAAtttgtggtgtttgtagctaactgatgattgatgagtgggcccttgctgtgtgagtgcctgacacatacagagtgcttgatgtatatgtgttGAAaGAAcgattattgacaaaaaGAAgattGAAggtgtgattgtaagagactttaacattatcttggttgtttttttaattgtGAAGAAaaataccgatggctaaagattttagttgctctatgacccagtcagtaataaccaaatgtgtgtgcacctgtgctctgaggtcacacacacctgcatgtatatacatttgtactgtacagagagGAAacaagggagagactTTCcctctttgcattgacatTTCatcattagatgtgttgccagttggcaccacccTTCctgctctgTTCtgctattgtcccctcatgatttgtcctcttgtgtgtcaccccctag  ***CLDN12*_3’ UTR (5 kb downstream of the last exon)**  gtTTCtgcTTCtgtttttTTCctcagtgttttaaggtGAAatattttgaGAAatgtTTCtcagatatatgtaaattGAAaaagacagTTCcatttaaagctctaaaacgtgtGAActgGAAggctgggtgcagtggctcacgcctgtaatcccagcactttggggggccaaggcaggcggatcacttgaggtcaggcccttgagatcatcctggccaacgtggtGAAaccctgtctctactaaaaatacaaaaaattagccaggcatggtggtgtgcacctgtaatcccagctactcgagtggctgaggcacgaGAAttgcttGAAcatgggaggtGAAggttgcagtgagctgagatcatgccactgcaTTCcagcctgggtgacagagactttgtctcaGAAaaagGAActaGAAagGAAttgGAAaacatgGAActtttaTTCcacaagtgccaagacgttttatccatTTCtaagagtagtccaagtagtcctgtggtcctaacagTTCactgggcttttttttTTCtccttTTCttttttttttaaacagtctgcaaagagacaagttgtgggccagatttgcctcatcaatggagttTTCtcagcctgtgatcttagagactgggagTTCagTTCtcaaacagttaagtagaggGAActTTCcgtactcTTCaaagctcgaggttaccaggaGAAgtggtcctgacaggcatgtcacctcaacatTTCtGAAacctgtgatGAAtgtatagttttggattacacctgtgttggggcagTTCttacatacacTTCtcactGAAacctgtggctctgataggttagatagctgagccatatgttgcactgGAAatcaGAAgctacTTCaaactgggttacactaacGAAcaacagtccgTTCtggactgttgtgcaggTTCactgagggtGAAtaaatggtagaGAAaccaTTCgggctaaccaggTTCaccagagactggtttaccaaccaGAAgtgaGAAatacatgttacttaGAAccatcagacacaaccagttttatccaaggcccagcacatcttaTTCtgccaaaaagtgagGAAacgctcaaaGAAtcatgggagcttatcaaatTTCacaggcgccagattGAAggtgctcccattggccaaatctgggacaatctGAAcatcaaaataaatgatagtaatgggttataactcattGAAtaaaatacaaatccatatgtccatgctgatataaatagggGAAGAAaGAAaagctctTTCttatagtaaaataccaaataaaaagtagaGAAaaaaatgaGAAaaacatcaatggggttaaaactagttGAAttttttatgagaggcaggatatttgcttagtctcaaagtacaaaatggcttattaatTTCagagagGAAaataactttatagtggataaacttggcaaccttagctgagtgatcaagtttgtcaccaataataaGAAaaagcaacatcatgcactTTCtgatatgatgcaaggaGAAggccacaatatTTCtgaggtattgcctgagtctaatcttGAAacatgcagctcattggtcccatttatgctttttgtTTCcTTCtctatcctgatctcatatctcTTCatttttataaaaaatgcataaaaatatagtcctatctccaaaatattttataaaataaactcttttaggagagattttaaaaGAAtttgtgtcagatcTTCtgtcctGAAaaactaaaaagtccctgcaGAAgagttgtaaatcaaagtcacaGAAacacaggactggatggtaacttaattagctgTTCcatTTCtgttggttagcagtgtcTTCaatctGAAagcgtactcacgttaccccacacatactagaggaccctTTCcaggagaggTTCaTTCcccctgtacctgctgggtggctcattaccctccagcccagccagcTTCtatgtcagtgccatagcatgcaagagagccctttGAAcctcactgcaggactgtaggacttggggaGAAaaagcacatctatatcctgcctggtgTTCatatccaagtctgctattacagggagatttgggttggtttgttgtattttgtTTCaaaatatgccaacttTTCaggttgTTCtataGAAaattgtaTTCagcaaaatttgggGAAgatagcGAAatggttgatgaGAAGAAaaaGAAcctttTTCttattttatatGAAaaacccagtgtgactggacctcttagcaaaagtctagtaatcatgattttggggtggtcttattaaccaaaatcctctttaGAAagatgGAAtatatgcttatatctccaggattttgctttatagGAAaaaaggcacagcTTCtcattaagaGAAaagacattattgatactttacattgtataatacctagtggccttgtTTCcaaggTTCactctcaaattgttGAAagtGAAtctagtaaataaatctagataactcacttacatttttaagtaacttttgtctttTTCtccatgccccctTTCttgacctcactTTCaaaagataatcttgtggtgtgGAAgatccaggtggcagtttagcaccaggaggctgctgcaggGAAaccccaggccctggtagcccTTCtctggggtgagcccatcTTCacctttgctggccatgtggatgagggccgagactcagctagtcccttgtgTTCcaGAActgctaacctgcTTCtctggctgcTTCtgctttaagagctcaaaggtagcagtggagtgctggagccaagtggtactggcccacaaGAAccagtgtgtacatctcctcccaactgcccaacctgtgacatcatattggttgcttgGAAttggccagggtGAAagtacttatgctctaGAAattgttaaatgctgcaagttagggctttgctttgccttTTCctgagagctgattattaaacatttactggcacaccgctgcttaggcaagacacgtttgctgagaTTCaggttagctaatgagGAAatGAAaaataatagcaatgctgagagttgtaaaatgttatctgtgataaaggggctatctGAAatagcctcttaacaggtctctctgtctccaatattgctctcctaccctaccacaaatttatctatacacagggctgcaGAAtgatcctaataaaatgcaactgaggctgggcacggtggctcatgcctgtaatcctagcactttgggaggctgagtgaggcaggtggatcacctgaggtcaggagTTCgagaccaacctggccaacgtggcaaaaccccgtctctactaaaaatacaaaaattaaccgggtgtgatggcatgtgcctgtagtcccagctactcaggaggcagaggacaggaGAAtcatctGAAcccgggagtcagaggttgcagtgagccgagatcgtgccactgcactccagcctgagtgacagagcgagactccatctcaaaaaacaaaGAAaaaatgcaactgatacctagtTTCtcccatacttaaaataaaatttggcagctacatcttaGAAcagtctaagcacaaagacatgagttgtctaGAAGAAttttTTCactgtgcccagagatgataagactgttggtgattattgacacaatatctcacaagatGAAtgtgtaaaaaaaagtGAAtttaataataaataacgggGAAtaTTCcttTTCtgtgactgactggtcacagtccttaTTCaagGAAtatctgtTTCctctattactcattattataGAAaaagtggcatgatGAAtgtggtagctagcctccaagatggacccagtgatcttgcTTCctgataTTCataccctgtgtattTTCTTCtcccattgagcaggctgtgttaccagtaggattttgcaGAAattatgatgtgtgacTTCtgaggttataggtcataaaagacatagcTTCtgttttgTTCtgtcttggatcacttactctggggtaagccggccacaatgttgtgaggacacatacagctgtaggattaacacagGAAgagctgaggccTTCtgccaacaatctgtaccaatttgccacccatgtaagagagccattttgGAAgtGAAtccccctaaccccaaccaaacctttagatgactgcaaccctggccagtgtcttGAAtgcaaccagagtggGAAactGAAaaccagcatcactcagctaagccctaccaccTTCttgacccacacaaattggatGAAacaatgcttattgttgttttaagctgctaaattttgggggggttgttttgcagcagtagataactaatgcaaagagcagttgcaggggatcaatcctgaggagtatttgtgctataagtattgtgagctTTCcttgctccatatcaGAAaagctctTTCtcatgatctcagaTTCccttgGAAggtgctgactatcgctataatgtggtGAAacaacttaatctGAAggtagggaGAAgGAAgatctgagcttagattgggagcagagTTCTTCctaattttggttaggtatGAAtagctgctgttgttaccaaagcaataaattGAAcactggtcatacatgaggtttgGAAtaGAAgataaacacatttgcaatcaaataaatctgatacacatagtagttatgttgtcatatggttagcaggagcaaataaaccaatcctataaaaaatacatcatctgttagtttttatccaactagccaattgtatctagggccaacccttttaaGAAgatacaactgatagtTTCtcaggtggGAAagGAAatagctgaTTCcaagacattaggcagttGAActGAAgtgatgcctccctctagtagcagtaGAAcactacagcttaTTCctcccactatagcactgcagggtaaccagagttaacaataattttattgtatattTTCaaatagtGAAgagaggattttGAAtcTTCccaacccaaaGAAatgataaatgaggtgatgtgtatgctaattg |
| ***CLDN15* (ENST00000401528.5)** |
| ***CLDN15* 5’ UTR (5 kb upstream of the first exon)**  catgtcagTTCccccaggactgtcttgatctggGAAagtgacttgaggtcacccaactaatcagttaatggctGAAttgggactaGAAgctcctgttttgtgagacctaggcccatgcccTTCcccccaacaccaccaccgtgttgcttagcagctattgaGAAtcttttgtggcatgGAAgaggtacagaggactgtcctacaaaattgtgagcagatagtgctttttgttttttgtttttgtttgagacggagtcttgctctgtcccccaggctggagtacagtggcgctgtctcagctcactgcaaccaccacctcccggTTCaagcaaTTCtcctgcatcaacatcctgagtagctgggattacaggtgcccactaccacgcccagctaatttttgtattttttgtagagatagggtTTCaccatgttggtcaggctggtctctaactcctgacctcaggtgatctgcccacctcggcctcccacagtgttgggattacaggtgtgagccacctcgtccagccagatagtgctttttggatggGAAccctatTTCctattggttttaggtgggtcatcatttgtcagaggccacaatacttaaccacttagtTTCtctgtagctccttgtgTTCaaataatcatttataagcacacagtcagtgtacagccatccctctgtagccatgggggattggTTCtagGAAccccacccccacagataccaatagccacccaagtcccgtatataaaatcgcatggtatttacatgtaacctgtgcaccTTCtcccatatactttaaatcatctgtagattacttataataccaaatacaatgtaaatgctgtgtaaatagtatactgtattgtttaggGAAtaatggcaaGAAaaaaaggtctgTTCatgTTCaatacagatgcaactatcttttttttttTTCtgagtatttttgatccatggTTCgttGAAtctatGAAtacaGAAtgctgactgtgttagcaaaggatgcctattGAAaatctataaaaatggccaggcacagtggctcacacctgtaatcccagcaccttgggaggccagggcaggcggatcacgaggtcaggggTTCgagaccagcctaatcaacatggtGAAaccctgtctctactaaaaatacaaaaattagctgggcctggtggcgcacacctttaatcccagctacgcagtaggctgaggcaggaGAAtcacttGAActcgggagacagaggttgcagtgagccaagatcacgccattgcactccagcctgggcaacacagtgagactccgtctcaaaaaaaaaaaaaaaaaaactataaaaataagactctTTCttaaaagatacaaataattttaccctatgtttgtctacTTCggcaaTTCagattTTCaggtttaTTCtacagcagggtgtaggtgtaGAAGAAacagcctctctaagtgttgccagtctagttgaGAAaacagtacagacagGAAgatttataaacacctgcaGAAttatttagcaacaaaTTCtagtgcatacagtacagttatcctctggGAAgtctctGAAttaaGAAagtcaggtggggccaggcactgtggctcatgcctgtaatctcagcactttgggaggccaaggccagaggatggcttGAAgtcagGAAtttgagaccacctggcctgtctgtgtaacaaagatgtttaaattagccaggcatggtggtgtgtgcttatagtcccagctacttgggaggctgaggcaggaggatcactgaccccaggagtttgaggctgcagtgagccatggacatgctactgtgctccagactggagtgacagagcgagaccctgtctccgttttttgttttttttTTCccgagactgagtgtgcaatggtgcaatcttggctcaccgcaacctctgcctaccgcaTTCaagcaaTTCtgctgccccagcctcccGAAcagctgggattacaggcatgtgccaacacgcccggctaattttgtatttttagtagagacggggtTTCtccatgttggtcaggctggtctcaaactcccgacctcaggtgatccgcccggctcagcTTCccaaagtgctgggattatagtcatgagctacggctcacggcccaccctgtctctttaaaaaaaaaaaaaaaaaaaaaaaaaagGAAgGAAgacaaagtcaggtggggtcTTCgggGAAggctgattGAAggtgagttttgagctgatttttttaaagggggagggagtcctaacttttttttttttaaacaataattgtacagcaggcagtttagttaaaaccacaacggcagcactttgggaggccagggcaggcagatcacttgaggtcaggagtttgagaccagcctggccaacatggtGAAactccatctctactGAAaatacaaaaattagccaggcgtggtggcgggcacctgtaaTTCcagctgctcggGAAaccgaggcaggaGAAtcgcttGAAcccaggaggcGAAggttgcagtgagccgagatcacgccattgcaccgcagcctgggcgacgagtGAAactccgtctGAAaaaaaataccacagcagatgccaaaactgagtcacgctgcctttggcTTCtccagggccccgatTTCctcatTTCagttggatggaccagttgactgtaatactgataaaaggcatgTTCtgtcactggggtgttggcacagctggggTTCaccctgtcccagagctctggctagggtgctgggagtgactggagctGAAagggaggcttaggactgtgaccctcccaggatcacagaggcaTTCagagagtgGAAggggcctctgggacacagtattagGAAagtattagGAAaGAAgcatTTCtcaccctcTTCtctgcccctccccagagctgctgcccaaagggagcaaggagGAAcagcgggattacgtcTTCtacctggccgtgggGAActaccggctcaaggtgaggtggggaccccgTTCccTTCaccctcctctggggctgcaggaccTTCttactgtgtcctccagggtggagccctggacagcggGAAggctGAAggggtgcagaggtgcagagcggtgtcagatgaggactccgaggGAAgatcagagtggcgagggggtggggagGAAggtgacgtcaagagactgggtgcaggcccaagccttgcgctgttatctcccgcagagttggggttgtttgtggactctactccgtggggtggggctggagggagggagtgggtggaggtcgagggaggagcccccagggctGAAGAAggGAAggcccTTCacgtgggcagggggagtggtggatactgaggtgtagccggcccagtggctcacgcctgtaatcccggctactcaggaggctgaggcgggaggactgcttgagcctgggagtttgaggttgcagtgggctgtgattgcgacgctgccgtccagcgtgggcaacagagcaagaccccgtctcttattaaaaaacaaacaaacaaaaaaacactgcggtgtgtatgacgcgtgcgcgtTTCcccgagcgcagGAAtacgaGAAggccttaaagtacgtccgcgggttgctgcagacagagccccaGAAcaaccaggccaagGAActggagcggctcattgacaaggccatGAAGAAaggtgacagcctcggcccctttgTTCagccccgctcTTCcccacgcctgggcactttggtctctctgtaaatGAAcccctccaggcagcccctcagccccaccccTTCcagTTCttagagccccttgccctgcgactccaggtaggggtgtgcctctgttgaggggcagggctggcgGAAggggacggtcagggtTTCcgcaggggccgggctcctgacactgtccctTTCcTTCcccagatggactcgtgggcatggccatcgtgggaggcatggccctgggtgtggcgggactggccggactcatcggacttgctgtgtccaagtccaaatcctGAAggagacgcgggagcccacggaGAAcgctccaggagggcctgtccatcctcgctgtcctTTCcctgTTCtccccctgccccccgtctctatcctctgtggccTTCagctaatTTCtgctcccctgagaTTCgtccTTCagccccatcatgtgctttgggatgagtgtaaataaaacggggctgtggcttggGAAcccccgtgtctgcgttgaggggaggggtgggTTCtGAAcgctgggcacagggggcggagacgtcactcctggtcctctcctgcccctgccctccaggttggcacggggTTCtttgtccaccccgcagcccagagcccacacccTTCcTTCctccctTTCctGAAgccatgtccttgagagtgccgggcagctcggagacatggggcttTTCcatggtcccagagtcctgggaGAAggcagtgacctgagcatgggcgggcaGAAcTTCggggccaggccgggcTTCaggcctgcaTTCtcctctgacccactcagctacTTCtgtggagttTTCcacagcaggcccccttggcTTCctcctccccaccctcccctccctttgtcTTCctgtggcctccccTTCctcctacctcctcctctgggggagggggagcccagtagggGAAgGAAtcctgcatctggcatggagtaggcgggggtcccgggagaggtcagagcccagggagccccagctctcgcctgtgggctgGAAggtcaggactcagccccacctagccagcTTCgccccctcgtgccaggctcggcatgggcctgttttgcTTCaccgcatacctctcgTTCcacagcccaggccagcacaggagtagggaggagacacctgaggggaggccggaGAAgcccagcagggccgtggggccagggaggtgcgcagccagcgctgatggcccccagggacagagccctaggGAAccggagcaggagatgagagtgtctgctctgggacaggtgct  ***CLDN15*_Intron 1** gtaagtctgccccccccaccctcgtggggcggggagcccggggcagcccagaggctggggggagggggtggacttttggcccgtTTCggttaTTCcctccatctcgtcaacag  ***CLDN15*_Intron 2**  gtatggggtggggagcgctccagggagagagggcggcgggGAAgctgcagagaccccaggggagggcgggtacTTCcgtgaggatgcagggagccctGAAttaagctctgcgggtctgagTTCccgcagccaggagtgctgggggcggcccatgcctaGAActggagtGAAgaccttgccacacgtggccccctgcgctagtgtggcgcttttagctgttacaggtgcaggcaccaGAAtcagaccccctTTCccagccctgtgctgtgggcaaatgatGAAaccagcTTCatctcccacctgtagggttagggtgagagtcccagTTCacaggtgactgaGAAagtgcaGAAtgttagcgtgatgttaacacacataggcactcagtacggttgagcatgttttgggggtgggattgctggggtgggcagggggaggaggccccatcttggaTTCttagaggttgatcaacTTCcaggctccacagactccccagcctcactgtcggggggcactggtctccttgtccggctgatgtctataaagggcccctgtGAAgggaggcgtcttgcaagttgcaggttgagcgtccgctgtaaggaggcggtgtgtgtgcaggtgtgtggggcTTCcaggacagtgtctTTCtggggtcttagagggctggagccaacagctctttgggcccagggcagTTCtTTCtgtggctgcggcaccTTCccgctccctgctccccgctaagatgaggccgccccattgtTTCtccggggcagtctcccTTCcgtctgccctatgccagagactgagcgctggcgaccgtGAActgtgtgtggtgccgctgcacgccctcctgggtccTTCagggccagtccactcaccaggcaccgtgtggcaggGAAggagccgagggcgacactggctgtGAAgcggggcttgagagctcacccccggggatgttggagctgctctgagcagttagggggcctgggtgggtctcctgtgcccccactactcccagcccctcctgaggcagcggcagaggcTTCctgttTTCatccatctctctaggactgactgtatgcagggccggcgggccccccccccaaaaaaaaccctataaaagctgagtacaacttgggccaGAAccccagagTTCtgagtgtccaGAAgggacactggaggcagcccctacacccacTTCccagacacatcatgctgtgaggagggggctctgctgtgagcctgcacacctgagaggggcacccctggcaactgcatGAAagatggtgccagagtccccagggcacaggggtagagggtgaccaggTTCcgggccttgggctaggtgcTTCtgcctacatttTTCcacagtgggGAAgtaggggGAAacttttacaGAAgcaaggtgcagcaccccaccctGAAtcacacaggcaggagaggggagccggcaTTCagactccacggctggggtggtcctgggagagggacctgactgcgtctcccaaccgtgcaccccagcccctggccacgcagcccatgtgcccctgggctcTTCcataatctctccattgactgctagagccacctggggactcagactcgtgtcagccccagagggagtggctgggagGAAGAAagtgctcccagaGAActttgtccctcctgcctaccccccgactctgcaccctgcatctcctggcagggacccagcctTTCcccTTCagcaccaacagttatgccccacccggGAAaggggtgcaaggtccttgGAAtgcttggcaactatcaaagacagaGAAgggaggaGAAggggGAAgcaagagggagcccgcagcctccagctctgaGAAaaggGAAactgaggcactGAAagactgagctagactgacctggatcggtcctgggcccaggaTTCcacctaggtcaGAAactccaccgggtgtggtggtccacacctgtaacctgagctactcaggaggctgaggcaggaggatcgcctgcatccaggagTTCaatcaaggctacagtgatgagctgtagtggcgccactgtcctctggcctgggcgacaaagcaagaccctgtctctaaaactgccctaggccctctgctgtacagcaccgctgccccctacctgttactccagGAAGAAaccaaggtcaaaatgtccagcactgggctaggacagtGAAggacttggagtgGAAtcagacgtgggGAAggcgacagcgatgcttagctgtggtTTCtgtatacccagcaacgtgagagcaacctgatagggcagttgTTCtcagccgggcgactttgcacaatgattgtcacagcttgtggggagggggttgctactggcaccccgtgggtagaggtcagggaggcTTCtGAAcatcccacagtacacaggacggcccccaGAAtagagttgcccagctcaggtgtcaagagtgcccaggaGAAagcctgtaatccaggcacaagcaaagcgtgccaggtgcatgggaggagtggggagcagggtgggaggggcccagatgcctaaggaggGAAgggtgactgcaactgggtaggctggaggagcccagggGAAggagaggatgtggggactgttaggtacaagagagcaaGAAggtgaggggggcctggcacagtggctcatgcctgtaatcccagcacTTCaggaggccgaggcaagcagatcatttggggtcaggagTTCgagaccagcctggacaacatggtGAAaccctgtctctactaaaaacaGAAaaattagccgggcgtggtggtgcgtgtctgtaatcccagctactggggaggctgaggcaggaGAAtcacttGAAcctgggatggtgaggggctgttgggctggctccgtcgcagaggggagatggGAAaggctgacaactgtgcccacccccag  ***CLDN15*_3’ UTR (5 kb downstream of the last exon)** gggcttGAAggtgtcaGAAgcggccggccaagcaaggggtgaggggtggtgccaggggtcggTTCaggtctgggaccccagactgccaccctgtccaccactttggggctgtgttgtacagcaaaataGAAgaggggttgcaagtgatTTCgccagtggcacataattgctggtaagagtcaccaaggagcagatcccgGAAgtcccggctgcagGAAactgGAAatagcagagatggcccctggccagcctgagaccccaagctgctcctTTCactctccatggccagtctagagcagagctgcgtgccctgggcctgagcatggatggttatcaggagtgataagccttttGAAaTTCtgtttaacattaacttggGAAtggtgcagagactTTCtccgggGAAggtacagtaccTTCcaGAAgagcctctggaggtctgtcggtgggtatgtgtgcacctcggtggtgtccggctgagacacggTTCtgggttgtgggtgggtttgctgcaggcccagtgggtgctaagcacagagctgcacccgcttaagatgagacagtgaggccaggtgtggtggctcatgcctgtaatcccagcactttgggaggctgaggcgggggagggggggtggcgaggaggatcacttaaggtcaggagtttaagaccaccctggccaacatggtGAAaccccTTCtctactaaaaaaaaaaaatacaaaaattagctgggcatggtggtgcgttactgtagtcccagctactcaggaggccaaggtgggagggtcgtttgaccccaggagttggaggctgcagtgagctgtGAAccactgcactccagcctgggcgacagagggagatcctgtctcaaaaaaaaaaagagaGAAaacaaaaggacaatgaggccgctagGAAGAAgcctggtatccagcaaagtgggcaatggagccccatcccagcactatcctctgcagtgcGAAcaagtggccagtcccttggggagccctggaTTCtgaccacagcccctcccctTTCtccTTCatctcaTTCgccctcaccaaccctcccctgGAAtggagagatctgcattggcaTTCcacctgtgcctctcccctgcgcTTCaGAAgtacatgtatgTTCctaccatgtgggctccagacacatcGAATTCatcactTTCcgcagagctgcagtccccatcacccctgtcaccagcatggctgacatctctgtctgcagatggacaacctctccctcccctctgctccaccacTTCctctcctccgTTCattgccaaatcctccagactctccctctatggtgtttTTCacacccatctacTTCtctctcTTCtccactgctggtcctggggcaggccctcatactctcctgcccccccacaactacagggTTCccccaactgggcgtctcaagcacggtcTTCctaatgcactcctggaGAAccaccgaccgccctgccttggcagctcctggccccgccaGAAGAAagccccagacagttatGAActtgacatgctgcctgggcctggcccacctctccatccccattgctccctTTCctccggataaaccgtgctccaccatcacaccaggctgcTTCctgggccctggacacaccTTCaccttggggctgggatccttgggatccttttggctgggctccagccaGAAacactggtgcttttttttttttttttttttttttttttgagacagagtcttgctctgtcacccaggctggagtgcagtggtgcgatgttggctcactgcaacctcggcctcccaggTTCaagagaTTCtcctgcctcagcctcccaagtagctgggactacaggcgtgcgccaccatgcccagctaatttttttgtatttttagtagagacagggtTTCaccatgttggccaggctggtctcaaactcctgacctcaggtaatccgcctgcctcagcctcccaaagtgctgGAAttacaagtgtgagccgctgcgcctggccagatgccctggtgccttttgagctgctccctacataaagggtcctgtggccaacaagtctgtgaccaactgcaatcctctccTTCcacaccgtcTTCtgtcttttacaggccaggctcagtaGAAgtgtcttttacaggccaggctcagtggatcatggccataatcccagcactttgggaggccaaggcaggaGAAtcccttaagcccagGAATTCaagaccaacctgggcaacgtagcgagaccccatctctactaaaaaaataaaaattagggccaggagcagtggctcacacctgtaatcccagcactttgggagactaaggtgggcggatcacctgaggtcaggagTTCaagagcagcctggccaacatggcaaaaccccaactctactaaaaatacaaaaattagccaggagtggtggcacacacctataatcccagctactcaggaggctgaggcagGAAaattgcttGAActtggGAAatggatattgcagtgagctgagattgtgccactgcactccagcctgggcgacagagcgagactccgtcttGAAaaacaaacaaaaagtgtcttacaatcctGAAaggccatgagagtgctccctatagtGAAgGAAgacaaaagtcaattacaactgctgacacctccaggcacTTCcaaaagtcTTCagtgtgccacccccacatgtctacctccttTTCccaacaccagcctgcctcTTCtcacTTCTTCccccTTCagGAAaaacaacctgtgcccagttttacaactcggcatttaagtgtgtgccttaatatggtcaccaggttgagttttgatTTCTTCccgggtggacatgtgactacaaagggttatggagacatttgccctttTTCaGAAcacatactgagctgcTTCcctgctaGAAgctggagtgggcctgtgccctcaaaGAAcccacagcctaggagcaaacacaaatgtaaaccactggccgggcgcagaggctcacacctgtaatcccagcactttgggaggccggggttggcggatcacctgaggtcaggagTTCgagaccagcctggccaacataatGAAactccatctctaataaaaatacaaaattattagccaggcgaggtggcgggtgcctgttatcccagctacttaggaggctgcagcaggaGAAtcgcttGAAcaaccagggaggcagaggttgcagtgagctgagatcgtgccactgcactccagcttgggtgacagagtgagaccctgtctcaaaaaaattaaaaGAAaaaataaaaaacatccaaaaactagcggggcatggtggtgggcacctgtaaTTCtaactactcaggaggctgaggcacgaGAAtcacttGAAcctgggaggtggaggttgcagtgagccaagatcatgccactgcactccagcctgagcgacagagtgacactctgtctcaaaaaaaGAAatgtaaatgcaaactgctggctacctgacaattgggccaggtgctgggtttagtccccagggagctggctgtGAAaaactcaacaacatgctttaggtcttggcagtatcatcaagcgctctcatttgctgcgtactcactaGAAgtcaggtctcgggctaggtgtattGAATTCttgcaatagctgcagcatggctccaacagcatcagagaggtGAAgtgacctgtccgagctacaccactagttagtactcagaccaggatTTCtccaagactGAAgacacaattgccctgcTTCccaGAATTCttggctctcctaactcacatcaattaaaatcaagatcttttttttgttttgttttgagacagggtcttgctctgttgcccaggttggactgcagtaaagtgattgctgcccaccacagcctcaacctcccaggcccaagccatccTTCcctgagtagctgggactaaaggtgtgcgccaccatacctggctaatttttaattttttttttttttttttttttgagacggagtcttgatctgtcgcacaggctggagtgcagtggcgcaatctcggctcactgcaagctccgcctcccgggTTCacgccaTTCtcctgcctcagcctcccgagtagctgggactacaggtgcccgccaccaggccggctaattttttgtatttttagtagagacggagtTTCaccgtgttagccaggatggtctcgatctcctgacctcgtgatctgcctgcctcggcctcccaaagtgctgagattacaggcatgagtcaccgtgcccggccaatttttaaattttttgtagagatggggtctccctgtgttgcccaagctggtctcaaactactaggatcaagagaccctcctgcctcagcctcccaaagcgttgggattacaggcctgagccacagcacccagcctcattGAAgtttttaggagtaaccacccgtgtccacacTTCcaaaccctggcaccaacctcTTCctcctctgtgctcccagGAAaggggggtgctgcccttatcacatgacactaggtttaccTTCtaGAAccttagagcagagtatacatTTCccaaagaccaggtgtctcccaaggtagGAAaaaatctccctagcatGAAgGAAgctggcaaaattgtTTCaggctgtccttgggGAAttatggttTTCtTTCttttTTCtttttttttttgagactgagtcttgctctgtcacccaggctggagtgcaatggcgtgatctcagctcactgcaacctctgcctcctgggTTCcagcaaTTCtcctgcctcagcctcccaaatagctgggattacaggcgtgtgccaccacacctagctgatttttgtaattttagtatagactgggtTTCaccatgttggccaggctggtctcGAActcctgacctcaagtgatccgcctgcTTCgggctcccaaagtgttgggattacaggcgtgagccacctgcctggccGAAttatggtttTTCaatgggggtcca |
| ***TJP1* (ENST00000400011.6)** |
| ***TJP1* 5’ UTR (5 kb upstream of the first exon)** ttttggcagGAAggGAAgggagtTTCtaTTCcttaatgtctgttTTCtctatggattatgagatgagatgttttgcctgggctggggagggagtcttaaagGAAtcatagGAAagtgtagatcagagtggaGAAgctgtGAAGAAtggaggacaaaccgctaagggatgagccagaggcctgtatgccagcctgcctggaTTCcagtcatggatttatggagcTTCcgacctgcctggttgtgcaatatttgggaccctGAAaaaaGAAaactccaccttagccccagtcagaggtaagggtagccccataggagGAATTCtTTCgagGAAtaactTTCtgtatactGAAttGAAtttaaaaagacttgttttttagtgtttatgtcTTCtttttaaaaaaaaaaaaatGAAatggtgttggtaatagctTTCtttttgtatTTCtaaactgtggtaatatgtacataatataaaatttgccattttaattatctttacatatacagtttataatgtttgttttaaaatgtttttactttataagattaaaaagttggtactcctatattgcacctctaagatcgttttaaaatttttactggtctatGAAatttaGAAacattatgagccttaaaaGAAtctTTCccccatttaacattgttacatgactttattggcagagGAAaaTTCtgtaacaatttgtaGAAtctttaatggtatggcataggagtgGAAtcaggattacttttggttgttataacatcttgataaagtgcTTCtccTTCtaatattttgtatataTTCtcTTCtctgacattatcgagattgtctaatgcTTCactaccgtaaataaatacTTCctctttgtcaagcgtaaactcacagTTCatataaaaataacataaatatgctTTCctaaggatcttttttgcaattaaaaccatacatatacatatatacaaattaggtgtggatatagttgTTCatttggtcaaggctgttttattaagtagTTCtatctaaaatgtgtaaagattttgcacacataatgcttttTTCaTTCtgagccgcagacaccaaaaatcccacagGAAgttgtcttttaagtgttgtggttttttagtTTCctgTTCagttgcagagatgatcaGAAataagataaatctgtacaatcGAAagagatttgtggttaaacattatagtaactctaaatgaGAAtccttaattaacttgtatTTCTTCccatattgatctagaggagctatgatgacatgcgtgtgtaagcataaaagtcaGAAaatacttttttggtgTTCcatggataaactctttaattgttaaatgggtgaTTCtTTCagattaTTCtttatactTTCcacttgttgtcaaagcacctacctaaaaaataaactgcagGAAtaaagctttgtttttaattgttggcaacagttttacctttattttacagatgataaaagccTTCtcttgtttatttaaaGAAcagactttatGAAgtttaatttagatattaaaaataccactGAAaaattttaaagtgatactattagtcactGAAatactaaatataatttttaaaGAAtaatattTTCaagatGAAatatttttgtttgatTTCattttttaaaccatggcaaagataaaactgttaaatatataactatttttaattagtgacaaatagttgcaatctagcatttaaattacacattgggcaacttgtaaaagtgacaaataagGAAaagtGAAaaatgtcagtgcatttgtagatagtgtctctagatttaggtgctattaaagacaccacacctagTTCtaggcagcttatgcaattgcacatcTTCttaaatggtaaagataatgtGAAaggtctTTCatcaccaccttttaaaattaaaaaatacTTCccaaacctttttaaatggtgtttgcatatGAAGAAatttaatcacaattttttttttttaatggggtctcactgtTTCccaggctggtctcaaactcctggcctcaaaGAAtcctctggtcttggcctcccaaagtgctgggatgatgatgatgatgatgatgatgatgatgatgatttgagacggagtTTCgctcttgttgcccaggctggagtgcaatggcatgatctcagctcattgcaacctccgcctctcgggTTCaagcgaTTCtcctgcctcagcctcctGAAtagctggcattactggcgtctgtcaccatgcccggctactTTCctatttttagtagagatggggtTTCaccatgtcggccaggctggtTTCGAActcctgacctcaagtgatctgcctgccttggcctctcaagtgctgggattacaggcttgagccactgcgcacggccccaagtgctgggattataaacgtgagcaactacgcctggccaccattttttttttttttttttttttttttttttttttttgagacagagtcttgctggttgcccaggctggagtacagtggcacgatctggactcactgcaacctccgcctcctgggTTCaagtgaTTCTTCtgcctcagcctcccgagtagctgggactacaggcacgcgccacaccaggctaatttttgtatttttaataaagacggagtTTCaccatattggccaggctggtctcaaactcctgacctcgtgatccgctcgcctcggcctcccaaagtgctgggattacaggcgtgagccaccacgcctggcccacaaTTCttaaaaGAAtaaataatactttgtccaaaagcaaaacaattacaacatgactttaaaacaaaaatgacTTCaaccatttttatGAAGAAcaaaaagGAAtaagggtttggtttgcaGAAggctcttTTCtggTTCagacagctttgggttgcaagatgtgtggcacTTCGAAgccttaattgatacttatgtatcacctacacatacTTCtttgttaaatttgTTCttTTCatacagttGAATTCtaaagaGAAaaccattgtctaaagcctgatgtttatgatTTCTTCcatTTCTTCtgtgccggttgtggcactataccagatactttaaagatgttatcattgatgttactaaggatTTCtggcattttttGAAgacctttacatttacaagtgtagattagtgagtctaggattggtTTCttaccatgTTCcttggtgtttagcaaagccgtcaacattgtggGAAgtttgtgtagtggctgattaaactggaccatggactgcacgtgtttacacccttTTCtgcccaactttgagttggagggacagcattgGAAtgcagggcagcttgacccgTTCggtcaacaaGAAtTTCagcaatcttgatggccaaaaataaacttTTCaagaTTCctgtctagatgggcggGAAagagactttggatatatcttgaggtctaatgtggggtgtgggccgagatgtcacaggtctctagggtgtTTCtcaggctgctgcctccgggtgcccatgacctcatcaccagtTTCagccttggcagtcggcgccggtGAAcgagagcaacgcTTCtgaccctgccggagctcctcggagatGAAagccatgacgcgccttgcaGAAaatgcaTTCcgccTTCcgtggGAAcaacgccgaggcacgcggtgacagccgtgaccatgctgtttgcccagtGAAgGAAacaactgtcgggtatcggctctgccggcctTTCcagccgcactcatgcatggggctcaccccatgatgtgcgtggcttgtcgaggagcaagtggacaagtctcttaagGAAagctttggtgcacaggcgctTTCtccttgggggcGAATTCtgccagaccttggataaaaacaaacagGAAgactcgcacggcagcgGAAactgtcTTCcaagttacttgggttacccggcttTTCcTTCcgcgcttggggtcgggaccccggccgctcgtcccgccccctcccccgccgcggccccgccccctccccgcctcgcctcgcctcgcctcgtccagccccgcccccgccgggccgggcatgctcagtgggccgggccggcaggtttgcgtggccgctgagttgccggcgccggctgagccagcggacgccgcgTTCcttggcggccgccggTTCccggGAAgttacgtggcGAAgccggcTTCcgaggagacgccgggaggccacgggtgctgctgacgggcgggcgaccgggcgaggccgacgtggccgggctgcGAAagctgcgggaggccgagtgggtggccgcgctcggagggaggtgccggtcgggcgcgccccgtggaGAAgacccgggcggggcgggcgcTTCccggacttttgtccgagttGAATTCcctccccctgggccgggcccTTCcggccgcccccgcccgtgccccgctcgctctcgggagatgtttatttgggctgtggcgtgaggagcgggcgggccagcgccgcggagtTTCgggtccgaggagcctcgcgcggcgctggagagagacaagatgtccgccagagctgcggccgccaaggtgagcgcctccgcggccgccagggccagaccgggccgaccgtcgccgcccgcccaccggcatctggcccgcgtcccgccctccctcgctggcggctgtctgggccccggggcggcggggtgggcagggctggcgcggggccgcgggccgcgggccgcgggccgcggggagcccctcgggcgggggcggcgcgggccgcactgggggcggccggggagggggctgcgggcgcccggccgccgtactgggcaggtgcatagctgccggcgcctgtgcctggctgcggctcgctgagggcggggacacgcaacaggtccctcgcggaGAAactcggctccagtgagggTTCgggggctgGAAgccggctctcagcgggtcggggcttggggtgccacctcctgctggccgggagctgctgtctttggaggagtggttggtccccggcGAAaccct  ***TJP1*_Intron 1**  gtaactttGAAaataacctctttaacatttaataatttaacttGAAttaaactTTCacaagtaatacaaagtaTTCctacGAAtggacaataagatgagcacttaaaaattagtaaaggccggtgagTTCagccGAAaaaagtaacgtttTTCctgttacttTTCctatgtgctctGAAatattattgcattTTCccattgctttGAAactaacttgtgtattacattaaaaagccaaagTTCctGAAaaacagctaggatgctcctcccattttgtatattaattttTTCatcataaaatagtacttgttatTTCaaacaaagGAAtacaGAAatgtgaggagtaaaaaatctcccctttaaaGAAtatcaaTTCattacTTCaaatagtatcgagatacttgggcaggtagtggttTTCtgtgtgtatttttgtttGAAatgcttacccgtaacatctgggagcagGAAtactatgcctctGAAacttttGAActctcacaGAAgcttgtttgctggctgggaGAAgctagcggttacacttattaatGAAtagattgttttaagggatatagtaTTCTTCgtttaaGAAaaatttaacttgcaccttttgaccttacattgagGAAGAAcaatctaaaggtaaaacttgatccagagatttaggataaaaggcaacattttgctttggttttaaaataagctcccattatttgtcttTTCtggggctTTCtTTCctag  ***TJP1*_Intron 2** gtaggaggagtacTTCtcctGAAttatagtattgagatttgtatggcatattGAAaagcatcactcgttaaatattacgttgtagggacttttttgcattgtaaacatttgcgagtaaagggcaagttGAAagcttttttttaagatgctgtattatggagccacTTCaGAAttttatatagagcaaaacaggccataaatGAAtaagtGAAatGAAataataTTCagtgacctgtTTCcgtaagtcacatttaatttttGAAggtgattaaaatgcttactaagagccaggctggactcagtgctttacatatgatgcgtttgttTTCcccaccctcctTTCagtttgGAAaaaaaaaaaaaaaaaggcaacattaatttgGAAgctcgctgttactgttttagcatgatagtttggGAAactcttgtctaataagccagtgcttttgaTTCggttTTCtttataatggttGAAtagtttgtcatcctatGAAtatgctgctgtttagattatctttgctTTCttgatagacatttgtTTCcaggattttgTTCttgGAAtagtagcctttttatatctgtacacatatattactgtatatcagaTTCttaaaaatgGAAttaccgaggcaaaggctatgtgcagtttaaaTTCtgatatatactactaaatgtTTCtctaaaaagactacctaGAAataaatataacaaatattggtgagctactgGAAtcttaatGAAggacataaagtgagatctGAAtaaacgagatGAAacaatgtattTTCtggatggagacagtcactgttgtaGAAtttactaattTTCcccaagtcagtgtcTTCtagtgttgtattgtaGAAaacttgacatattgataaGAAaacctacatgataaaatgatagcgacaaaattttgGAAaaaGAAggccgtatcagGAAacttgttaGAAaTTCacagtcatttaaagcagcccgtcagTTCagGAAccagggaggtccaGAAacatctgtgcatttatgggcttttagtaattatgatcatcagtattattgcatgtcaggtattgTTCtaagtgctcaatttatttaaTTCtcacagtaaccctatGAAgtGAActattatctctactttttggtGAAGAAattGAAacacagagatacgataacttttaagTTCatttagccagcaaggtgGAAaactacGAAtatgtggtaaaaaataatattgtagactattggaGAAagtggcttgTTCaatgtatggtgctaggactattggttatccattttttaaaaatgttTTCtgcctcataggctaaaaaaaatcaaagGAAatctaaatgacaaagagcaataaaaaTTCtaaaataGAAGAAaatgtaGAAtactacttaaaaatatgtatgtacctagattGAAaaGAAtcctccgtgcatgatactaaaGAAgtaataaaggcgggtgggtcacttgaggtcaggagTTCgaggccagcctggccaacatggtGAAaccccgtctctactaaaaatacaaaaattagccgggcatggtggcgcgtgcctataatcccagctacttggGAAgctaagGAAggaGAAttgcttGAAcctgGAAggcggaggttgcattgagctgagatcggggccactgcactacagcctgggcgacaaagtgagcTTCcgtctcaaaaacaaaaagtaatGAAgGAAaaaacgtggccgggcgcggtgtaatcccagcactttgtgaggccaggaggggcggatcacgacgtcaggagTTCaagaccagcctgaccaacatggtGAAaccccgtctctactaaaaatacaaaaaaaaaaaaaaaaaaatagccgggcctggtggcgagcacctgtaatcccccctaatggGAAggctgaggcaggaGAAacgcttGAAcccgggagtcagaggttgcattgagccaagatcgcaccactgcactccatcctgggcgacagagcgagacgctgtctcaaataaataaataaagGAAaaaaacgttttttatatgtgtgataaacgtctgcgTTCgcagtcaGAAttttgtttactggtgtatttgatttaagattattaGAAagttGAAaaacaaGAAttagggtggTTCtctgcgtcaccaaaGAAttgtttaaatgtgtaaGAAagtggtatGAAtcattgttaaGAAacgatTTCattggttGAAtcacaGAAaagtggctGAAgGAAgtTTCagtggcTTCtgtgttTTCaagctgttTTCctttggagGAAgcaataaaatttaatttaagattgtgctattgtatatgcttattagattttgctGAAtacttaaatgtttttgttaagtctcggGAAtagtagtattgcccgctgtggctatctGAAattataatgtaagtatGAAaatgatttatgtcattttttactgttacactgatttacatgatacccagGAAttgatttattgtatcaagtggagcacagtgggcttttttttttttaattaagTTCtgggatccatgtgcaGAAtgtgcacatttgttacataggtatacgtgtgccatggtggtttgctgcacctatGAAcccatcatctaagttttaagccccacatgcattaggtatttTTCctaaatgctTTCcctccctTTCcccccaccaccaacccgggtaacaggccccaggtcctggtgtgtgatgTTCccctccctgtgtccatgtgTTCtcattgTTCaactcccacttatgagtgaGAAcatgtggtgtttggttTTCtgTTCtggtgttagtttgctgaggatgatagcTTCcagcTTCgtccgtgtccctgcaaaggacatGAActcaTTCttttttatggctgcatagtataccatggtgtatatatgtaccacattTTCtttatccaatctgtcattggtgggcatttgggttggTTCcatgtctttgctattgtaaatagtgctgcagtaaacatatgtgtgcatgtgtctttataggaGAAtgatttataTTCctttgagtatatacccagtaatgggattactgggtcaaatggtatTTCtggTTCtaggtccttgagGAAttgccatactatcTTCcacaatggttGAActaatttacaTTCccaccaacagtgtaaaagcgTTCctatTTCtccacagcctcggcagcatctgttgtTTCctgactTTCtaataatcaccaTTCtgacgggtgtaagatggtatctcattgtggttttgatttgcatTTCtctaatgatcggtgatgatgagcattttTTCatatgtttgttggccgcataaatacctccttttttttggcagcataaatgtcTTCttttgaGAAgtgtctgTTCataTTCtttgcccagtttttgatacggatatttgttTTCttgtaaattagtTTCacTTCcatgtggaTTCtggatattagacctttgtcagatcagtagatggcaaaagtttTTCtcccaTTCtgtaggttgcctgTTCactctgatagtTTCttttgctgtgcaGAAgctctttagtttaattagatcccatttgtcaattttagcttttgttgcaattgcttttggtgttTTCatcatGAAgtctttgcccatgcctatgtcctGAAtggtattgcctaggttTTCttttagggtttttatggttttgggttttacattGAAgtctttaatccatcttgagttaatttgtgtatacagtgggcttTTCttgatatcttaacgtttttGAAaccttaagagctgcaGAAaatggccaagctaatGAAcaaaaattaatgacaagagtcagtcattacatatagggattgtTTCttgagtgagtgatgcagtgttttaattatgatagttactgatcaatgcatacttgcTTCatactgttttaaaagtGAAaaactcactgtagtttgtgaGAAgtcactGAAgtttggctgccaacttttaatgatcctcactgtagcaataatttttgtgtgtgtttggcattttagaccatcatggttaaagctttaatttatatagttggctgataatcctaactacattttacaactcttacatttgtTTCaggacagcTTCataagatagttatgtTTCaaattgtggtgTTCTTCccccccaggttatatattggcacaaattacattatattattagGAAagacaccaatttgccagactcaagcatgtaatccctgcactttgggaggctgaggcgggcatatcacctgagggcgggacTTCaagaccagcacaaccaacacggccGAAaccccgtctctactaaaaatacaaaaattagccgggtgtggtagcacatgcctaaaatcccagctacttgggaggctgaggcaggaGAAtcatttGAAcccagGAAgcagagtttgcagttgagccaaggttgcagtgagtcgagatagcaccactgcactcctgcctgggcaacagagtgagagtctgtctcaaaaaaaaaaaagGAAagacaccaataaataatgaggtgGAAaccaatctgtggcTTCgagggctgtaacgcagtatatgtagtactgcTTCccgtttgatttGAAgGAAaaaaagtacagtagTTCcactccctttTTCccctccaatcctgGAAacaaggtctggctctgacttttacttgctctttaatctcttTTCtgtttagagaccaaatgtaaactctgtacagTTCtaaGAAtaaaactgtgtataaatatgatagatcaccatgctgctaagGAAaatgtctttaaaaataatagttttggtgGAAaatttGAAgacgtaatcctgttacttgacatacccctgatatgataaaaaTTCaccttactGAAagaccatgagtccagctgtaaaTTCatgtgtagtgccacccatctgtgactgctcagtcattgctcatTTCaggcctgcctGAAagcaTTCacgtgctcagcattgtgtatgactgagcattatagtagctGAAgacaagctagtgGAAtgagccagtcatTTCtaattagcaaagttttgatatgttaaataaaaagatagtttatgtctaGAAtgatcctcctgGAAtatttgactgGAActtgctttgcacctctGAAttttGAAagcaaatcttGAAaaacctttttactttaacaacttttagtcttgtaGAAaGAAatagacattaggatagtttacagattttaaagttaatcctataaaattggtgtgttgcagtatTTCttgcataagccagcaataaaaataagacttttgctctctctgacctagagtgcatctttatttatttattgcagaGAAgagtggcTTCaggttgctcctttatgggctagccaggtgctttatTTCcaTTCctcttaaacagataaaTTCttaataatgagcagtatgtgggGAAtacgtgggtagggagttttatcttatatcctaagcatggatGAAaataaatggactctGAAtgattgcctttaccacaaGAAcacagctttagatcatgaGAAcaccaatgttaccaaactttgtactgtaaaacaccatttGAAatgtgataacaaaaccctgtcatatatgtagtgctcaatactGAAtctcaggtggccctcactttgggatagatcaagagataaggttttattagtttgtgttgcggatatatTTCagttgatatactGAAaatactggGAAgtgagttaactggtGAAtgatctcaaTTCtagtttatcatgtccgGAAaggaggtaaaacaGAAgctgatattactTTCtgtggaTTCtaattagGAAcataagattagcgtatacctggtttaattaattttttttgacttaccGAAatattttTTCctttttaaaaaaatgtaaaTTCccaaagactGAAaagTTCatttTTCTTCttggttatcttagGAAttTTCacgcttttgGAAttgtcattatcttggagattgtacaacagtTTCtggtaactGAAtatcaccagttaaacttaatataagattagctcacttaatttagtatgtaattttaaaaTTCaggaggataccagtaatgactaaTTCtttttgttagcttaacacatattaaatgagtacattttgtttgggatcccgatcctaGAAatttacagttTTCaactgctatacgctgtaatgatcactttgtGAAttgtgtaactcattgttGAAGAAacaaaggatttgcattgtttactcttaaaaatacgagcaaacttatggtcatcctgtaataacagagcaTTCttgtgtgctTTCTTCgttggaggaggtgacaaagtcattTTCtaaacctgggcttgctgtttagcagagtTTCtgtatttTTCcttgttaatgtttttaaatgtttGAAttGAAtctggatacattgatgtaagtgttTTCtgcatatgtttggtatcaatatctcaataggagggagacattaaGAAttataaagtaattggcttatatgcttaacagctccttGAAtatactgacaaggcaaatGAAcactatactagctgttactgttacatttagggTTCaaGAAgctgtGAAatatgtaacaatttggaTTCctaaagcgGAAttttacaTTCagcagatatttgttggttacctcgtgtgtaccaatcactgttgtaggtgctggagatgcagcagtGAAcacatctctgtactgatagggcattttaatggtgcgggtagagagcagcaaataaaaTTCtgtccataatgataagtgctaGAAtccctaacatgccttTTCtgggtcatatcctcccTTCTTCaaagattgcatgtatcttgtcTTCtaggtTTCtcattTTCtagtttTTCtttatagttaccacctaattatgcaTTCtGAAagcactagttttgactctctGAActttgcgcatacaGAAtcTTCtagtgtGAATTCtgttgtatatggcTTCttttgctcagcattatgtttgtaagattatgttgtggaGAActgtagctcattagttTTCaTTCagTTCttgtacTTCatggtaTTCcattgtatgaggactttattggtTTCgggaGAAaatagtTTCagttagacTTCtgtggctttttggaGAAaaatattgtatatgtctgtctgaggggcaaGAAttagatatgcattatgaggcagtcattggagtaccaacagctgagcaaaaggtagtaagcTTCttttggtgTTCctatgattatcttGAAaaatcaagttggtcttagtttggaggatattttatgactaagatgtttgtcatactaaagtatgttttagtagtaagtattGAAgtatgttttagtagtaagatatccacttacctgactgatcagaccaccaaaagccacaatcagggGAAatacatGAAtctggcaaaaatgagtctgcctTTCaaaatataattgtgGAAgtttTTCctggtaatgtcatataaaggtactaagGAAggtttatttactgattttttttTTCtaacagtggcagttattggagtattaatacttactggagtgttaatacaGAAgtacactattttgatgctgtatcacctataaaaatcaagtcagtagccatggcaagtaggcttacattttagttataagtgtgaGAAatatgtgtcaaaacaaacagtagccccttatattgcatgctgactctgtgacagatactgagctaagtgttttatgtgcattatcccatttaatcTTCacaaTTCtgttagatactacgattatctctgtttttacaggtGAAggatctgaggttaaatttacttgccataggtaatcatacatctacttacatgTTCtataagtcatacaTTCacatccttttatgTTCgtgtgttgtaagagagcaccagggaGAAgagtatgggatggtgttaggtaagtcttagaccaGAAaagcagcatGAAtcatGAAagccaGAAgagagggcactgctccatcagagcacaagccagtttgtctaaagGAAgcagctcttgctcagccccagctgattgttTTCttgGAAGAAgacaggccagatctcccagtttTTCaagaggttgcaaatccggattTTCatgtaagttaccctgacttaagtcttgctagttgacttagttttttatatctttttattgtGAAatatattgccatgtgtacaaaagtacataaagcaaatgtacattaatggattattataaagtGAAcacccgtgtaaacaccaTTCacctgtcagcaggccagatcctgcacagagaggtcaagtaaaataaggactGAAaagattatcaccgagcaactcttaagttttacttatatgtgagtttagcctctgGAAtctGAAtcctgagtctgccactaaatggctgttTTCtcatctgtacacaaaatgcaaatgtaaaaatgcaaacaagacccatctcagagtgattgtgggctcagcacatactaagctattatgttagctgtgtcTTCtaaaaacattttaagtGAAgcctattttaatatcagagaggaggcatatagGAAttttatttatttacttgtttatttatggacaaaggttggcctgtagattatttaagacaTTCaTTCaTTCaTTCaTTCaTTCaTTCattttggacagagatcagcctgtaggttagtagttTTCagcccagcaagatgttttgtggtTTCaGAAggaggaTTCatttatttatTTCtggacaaagatcaatcagcctgtaggttagtagtTTCagcccagcaagatgttttatggtTTCagagGAAggTTCcttgggTTCctgagatacttttGAAaaaattattacaatggcattagataagagatacagtccttagtctgatggattaggcaatagagttGAAagactaTTCcaaatccagggtacctggctcaatttggatgtctacctagtatggtGAAtggttgattagcaagTTCtaTTCagctttgatttgctgagcacctcaggtctTTCtacttgttttatccgtactTTCaaccatttacaTTCtccctcccaccacccccccacccccacccaagtaatcctccctctggaTTCctgtaatagcctcctaagtatcatacccccctcTTCcccttacaatctgtttTTCtGAAataatcttTTCaaaatccaggtttgattagtTTCtctccgctctcccccgccccgctacacacacatgcGAAcactcatcctaaaaTTCctaggctgcttaactacctttagagtaaaagatcaaaatccttaatatggtcagtaagctcctagtggattatttggcccctgcctacccctctagccacatcgtatctttTTCctccttgtTTCttgTTCtcaaactgtgctTTCctctttttggTTCctagcaagtgtacTTCctgtcaactTTCtctttgagTTCctcatggcactgtttTTCcTTCtTTCatagtacttatttaactttgtgactatatatttagccatggagtttTTCgatcatttggTTCtcctgctagactgtaagctccttgaGAAcaaaggtcatactagttgtgtccagttaTTCgtgtagtttaacacagtgtctagtacatagGAAactctcaaaacattatttGAAtGAAtgagagatgcactTTCctacactaggctcttgcacgtgctgttttTTCtacccgGAAaccttgccacctccatctTTCatgcacctcatagctgtctcTTCtcctcaaggactttgcccagagcccaGAAactaggtcaaaactctgtgctttaaactctgtgtacccctttatagcacttatgataacctgtacttagTTCtctgctattatttgattagtatctgttaccccTTCtagacaagagttgccttTTCaatgctgttaactcacttactggtaatgttagaccaccttaaaTTCagcttagcagcaGAAgGAAggtactaatccatctccaggactagttgcatttTTCttactaTTCtGAAaggggagggGAAgcagttgctgGAAtagccagctcatgGAAatactacttaatatcctggcatcatggtagctgcaTTCttggtttgggtctTTCctaaatcagtaattaatTTCtagtcccctcaagttaccTTCttttaagtgGAAaattTTCaaacctataGAAaagtggatgtgggGAAtataatGAATTCccacattactgtcacccagtatggttaacaacttacagccagtctcatTTCatctatacTTCaTTCattgtgtcTTCtcTTCcccattattttGAAgcaaatgccagatatcttTTCatctgttagtacatagtGAAcatcccaaatctGAAatgcTTCaaaatccagtactttttgagcactgacataatgctcaaaGAAaaTTCtctttggagcatTTCagatTTCagatttttggattttggatggTTCaccctGAAatcgGAAacacgtggtttGAAgcatTTCagaGAAgagataGAAtgGAAtactcaacatgtgtctGAAtatatatctctaaaagatgtcttttttaaaagtactataactacattatcattagcacacttttaaaataaactccaaatcaagtacttaagtgtttggttttttttttttaagacggagtTTCgctcctggttgcccaggctggagtgcaatggcatgaccttggctcactgtaacgtctgcctccctggTTCaagggaTTCtcctgcctcagcctcctgagtagctgggattgcaggcatctgccaccatgcctggctaattttttgtatttttagtagagacggggtTTCaccgtgttggccagcctggtctcGAActcctgacTTCaggcgatccacccccgttggcctcccaagtgctgagattacaggtgtgagccaccgcgcccagccatgtttagtgttTTCtaattgtgttaaatgtcaactTTCcttgatgaggtcctcttactgtgattgtttaacatTTCtcttaggcatatgaggtcccccatTTCtgtctctctctTTCccctcTTCccttgcagctttattggttgaGAAacaggattatttgtaGAAattttaacagtatGAAttttggtatttgcTTCcttgtggtgattacaatggtggttggcccactgtatTTCctgtaaattggttaactgtagctagagcaaTTCtgtctaatgtagtagccacaccaaaactttaaggtaaaattaattTTCatgttttatttaacacacatatccaaaatattaccattttagcatgcaatgatataaaattataggtgagatattttaaattattTTCcataTTCagtctctGAAtactgttgcatgtatattttacatatcatgtatattttgcacatagcatatcataatacatatcacatatTTCgtatcatatatatattttacatataccatatctcagtttggactacccacattttaagtgctgagtagccccatgtggctggtggctactgtactggatagcacagatctagagccttgatcagaTTCagatttgatttttttgtttgtttttacagTTCtacTTCataagtggcaTTCggtcccagggttttTTCtgttgtttttggatgatgtctgcagccattgatgatcagtgtgtagatcccttaaTTCactaggtgttacaaaatgatgataTTCaatttttattaTTCctttTTCattggttagctggagtgcTTCtaaaaaaaGAAactctccTTCatctactattttgatcgcccactggtactgtttgtatagGAAagacagagtaaatatcttaTTCtTTCcctatacgtaccagtTTCtcaaaataatgagTTCtgccagcacttatcattttttaaaatcactgtgggcatatggatggatgGAAgcatatttgatttattttaatccattgcagtcaaattacttacagTTCtgtcaggttactttattttGAAtttttaaataacataatacattaggcaaatcataggtgtacacagattgatatattTTCaaagtGAAtatgcctatttgcccacaactcagatcaagGAAGAAcatttactttacgtcccGAAgctcccccacctccatcccTTCgagttacTTCatacacaaaggtaaccctgtcccaacTTCtaacagtgtagatcacttTTCcctgtTTCttgtttatgtttttgttttaagacagtctcactgtgtcacccaggctggagtgcagtggtgcaatctcggctcactgcaaactccacctcctgggTTCaagtgaTTCtcgtgcctcagcctcccaagtagctgggattacaggcatgtgcccccatgcctggctaatttttgtatttttagtagagacggggtTTCaccaggttggccaggctgatcttGAAcatctggcctcaaatgatctgcctgcctcagcctccaaaagtgctgggattacaggtgtgagccaccatgcctggccacttttgcccattttttgtTTCgtaaTTCagatgtgatgtttaccagttTTCaaggattatGAAaTTCatgtgTTCattgtaggatatataaaaacttaaaatatacaactctGAAaccaagttttttTTCtgagtacgtaaatattttTTCcaaaattggtatctTTCtgcatttTTCtataccattataacttttttaaactttttattttaaaatattagatttataGAAaagttaaactcTTCtttagtgtggttacaTTCtccatctgtaatatagttatgTTCatttgttactgtctttaTTCtgtttttgatTTCctcccctcccctcatgtctttttggttttaatagtttaattttttgatctgtGAAgggtaatgtcatGAAacaatactgtggtctaaGAAagagagttatgcaaaaagatatactcagaggactgtcactctctcTTCatctctgctatgccaTTCccTTCctcctcctgtctacctGAAtaaattgtgagagatctttgtatctatcTTCaacctttaGAAgatgctatacctaaaaatagttttatttttgataacttGAAagcactctagattttaagtTTCcaaGAAtatttgtaactgtccctactgagattTTCtcaatatttaatgtacacataatggtaacttttaagctgatgTTCtTTCactcttactttatagctaatgtagcagcttatctgtGAActtaatgtttttagcagaccTTCagctttgatcaaccaGAAaattacaggTTCactgtgtctgtctTTCcgactaatatTTCctaatcatatccatctcatcatttgtTTCctaaactgttaaccaagggatggacacagtgatacatttgtaaatttaaccagtgttGAAtacagtggggtgttggatgctaaatttactaaatTTCtattgagacttttatcctatggttgctgccTTCttattttgatcattattttaaTTCttatgtTTCctGAAtttttatcTTCtaTTCatGAAgtttgttGAAtccctGAAtatcgttaggacccTTCTTCattttattttagagtgatcttttttactacctcctggacatTTCtattttggGAAtttaaatgacggcccatcagatcagtccaaatatacatattttgccttaccttttaacatgtagcactaTTCcctttgtacTTCatttgtgttgcagtgatcacaatttGAAattatgtaattGAAacTTCattGAAgggcaggggtcatctgttttaTTCactGAAacctaccttgtccTTCtccttaagTTCctggctcaacctcatctccatagatgatcttgctTTCaatttggatgGAAaaaagccaaaaagaccaaaacaGAAacactttggagTTCaGAAgGAAtgaGAAgccgTTCagtgtagcagctttgctcacaGAAgGAActtggagggTTCacagagtgTTCttaatacctttactattggttTTCctaatgggGAActgcatgtcatcaaGAAagggtgTTCcttGAAgatttgtgGAAattttattattttatgatgtcagataaatgaggtgtattgatctaaaaaattgagggatcttaagctttggtgtaTTCtctggacagtaagtagtgttttgtattagctccattggttTTCatgtatcctcattgtctgcTTCTTCtcaaaTTCctttggttgtccacagggttatcttagtccttgggtaattacatcttTTCcttttgacTTCagcactgctacttggttgactactcaagagccattttGAActccacccacacTTCtcactaactTTCtaactggtTTCcctTTCtcaaatcattTTCccattacagccTTCaggatGAATTCttatatcatttgatcgtcGAAaaTTCtttagtgtcTTCcatttgagtttaaagtccttggcattatattataaagcctgttgtgatctggtacccttgcaccTTCtctccttttactctctccttgTTCcataaattgtacaacttagtcatatcaaagccagcgtcatgtgtagcatcacacaatcttgctgacatgctgtTTCTTCtGAAacatctTTCtccacttttTTCctggacatccTTCaagcttgatGAActcaaaacgttgctgtctcaggGAAcagatctctgatcTTCacagcagacacagtccttttgcatcTTCcataacagctcttatctatgtggtgcaaaagttatttattttggtgcaaaaagctcatatcttttgatgccattttaatggcaaaagctgcagttatttttgcaccaacctaaataccatttagtgTTCtcagctgcTTCtttgagcccctagggagggagagggacctttttttggcctccccaacacttaccatgtctgatacatgtttgttGAAtGAAtGAAtGAAtGAAtgatacatggattggttttGAAattacagccataaggttagtaaaatttaactggctgtgtatttagataatgttaaggagTTCTTCatttggagtatactttatatcTTCtcttttggatttgTTCTTCtcccagttataggctcatcctgtcttTTCagttattgtgccctcctatctTTCcccaTTCagtaatcagtcctGAAggacagtGAAactgcctaGAActgggatctGAAgttttggcagGAAgcccTTCagagttggtgatctgagtagtgtctttgccagctggagcttaGAAcaaaGAAactaaacaaactcttggcagattgtGAAatacagctttttgcTTCTTCaGAAattgtgaTTCtagtcagttgatgctgacttaGAAatgtgcgcaGAActcaGAAtccagcaaccatGAAttgcagtcacttttgatgcctgcagtgcTTCactaattattGAAtttgtagcctgTTCattgGAAttatagtctgataGAAttttggagctaGAAaagtcttTTCatttgagataatTTCacccatTTCcctaattgtgtaggtgagGAAaattaagatcacgtTTCtagttgatgatacctTTCggctctggtctccttatgcctggtctagTTCatTTCctgtttaccagcatctgtctgagttactaggGAAtggtagtgctaGAAtcatgcttagaggagtaatacattTTCcccttggtctcctacaagcctaaatgtcttGAAcctgagactggtaatttaccaGAAgctttttgtgcaagttatgcagttatcccttgtgcatgtttGAAatgaGAAaatgacaagcaagtttacaataaggatattaagttttaaaaataatttgataagtaatagatGAAtgtaattaaaagtacaaagggctcccagtGAAaagtgagactcctcctacctggttTTCctTTCtccactccaGAAgccatgcgtTTCttaggtaTTCTTCcaaagGAAcctccacaccccttgcttTTCtaatgcatcaaaaTTCtcagtcattagtGAAcattttTTCtttatgGAAtgctcattaaaatagtggttaataaatttactggtactaaaaatgtctcatatcaattttagctttgatTTCtgagtgatttactaGAAggatTTCttagtttTTCctcagcattttattatgatcactTTCaaacacgtaacaagattGAAagcagtctgccatggttacctGAAtcccaaccactaagaTTCtaccattatcattttactgtatttgcattattacatatttacgtgtttgTTCatccTTCcaTTCctcaTTCaGAActcctcatcagtactgcctggttatataaacaaccatggataGAAtttgctacttttgccacaGAAgcaaatGAAaggctagaGAAtggagagagatgacatggtccatcatttaaactagGAAtatatttTTCtgttttttgttttttttttttaaagataaggagtggtaaaacatagtaactcacttatattatggattaagttggtttagtggctggtcaagagcctaaccacTTCttttgatgtTTCaTTCtcaattactTTCTTCtacataggtcTTCcTTCagataaatcaagctataattgatgtttaacagaGAAgttttttgagatgtggacttTTCcagtcctccaaaGAAaaactatattgattttgatgtgGAAtggtgagagcaattatgttttagtattttagtcTTCtcccttgtacaaagcaagtTTCtcttattgcactcctcctggagactgtacattaagaccttaatgtatagattgcttactaacaggaGAAtaaacattttttataattaacaataaGAAcattaatcctcttaagcattatttgattTTCctaaaagccaagtgGAAaaacagatacagatgcTTCtcagctcacttaGAAcaatgtcctgattaaactcatcgtaagttGAAaatactttacgtcaaaaatgcatttaatacacccaacctacccagtatcataagcttagcccagcctaccttaaccatgctcaGAAcacttacattagtatatagttgggcaaaattatctaacacaaaagcttacTTCacaataaagtgttGAAtatctcatataatTTCttGAAtactgtagtgGAAatGAAaaacaatggttgtatgggtaccatcgtaaagtcaGAAaatcaagttgacccatcctaagtcagggactgtctgtaTTCGAAatgtcaaatagGAAtgagattttgtGAAtatagtttaaaatacattgccagtttgTTCtgatgggGAAtggaGAAagGAAagcagGAAgataaaacacctgataGAAaaatgtcacccacagtccctttactttGAAggtgataaacaatgttaacattttgtttatcctttgagcctttaTTCcctactaatgtttaaacaaaattaggataaccatgtacacgctgttttacaacctgctccccccttaatgttaaataaattTTCccatttgattaacatcTTCttacaaaatgGAAaactgtgTTCattaagaccttagagaggatgatGAActgtTTCataccatagagtatgttatcaaagagagttaagtgcatgttatcacatcacttgccatttggactgatagGAAaaagattaaaaaggcagtaaaacaatgtcactggttaatGAAatagatcaaGAAaagatGAActgagagctatgcaagcagTTCtcaTTCttaatatcagctgagattggacaaactggcaactcttgcagatacttttatcatgtgtatgttagtgggactgttgatgtttagctgatttactcatactattgttgcTTCtcattgatgGAAGAAtttttttttttagtgcattatcccggtcaatgtttgtttaaaaaaaaaaaaacagctttgtTTCcagtggaggtctcattaaagggaggttttggtgcacTTCattgGAAgattGAAacaaatgctggtgaggttggcagTTCttatctatgggagtGAAcagagagatcctTTCtctctcctctcttaTTCatctggcaggataatctagttgctttGAAtttaggGAAgcaggctTTCtttatagggacttactTTCtaaaatggcattaatcTTCaattaaagtcgctgtggGAAaggagaGAAtgggcccatggcaccttggtaggcataTTCtcatgatgctaatgacagcagtaagaGAAaacagacctcacttatGAAataggcatttatagtaatgggtgactagtgatctgagagtgtcatgcttagccTTCtccctttttactttaccttttgataactctGAAGAAtcttttttaaatttacttgtaaatacattttaggagtcccagtTTCataaaatggtaacattaagagagactGAAatattttaagtctccaagagcaattaaccccatttaaaaGAAatttattttGAATTCcttactgctaaTTCcttagatcaaccttgtccaacccgtggTTCacaggtcgcatgcggcccaacacaaaTTCgtaaattTTCttacaacattatgagatttttttttttgtaattttttttttaagctcatcagctTTCattagtgttagtgtattttatgtgtggcccaagacaaTTCTTCTTCtTTCaagtgtggcccagaGAAgccaaaagattggacacccctgccttagataataatttattTTCggccgggcgcggtggctcacgcctgtaatcccagcactttgggaggccgaggcgggcggatcaggaggtcaggagatcgagaccatcccggctaaaacggtGAAaccccgtctgtactaaaaatacaaaaaattagccgggcgtagtggcgggcgcctgtagtcccagctacttgggaggctgaggcaggaGAAtggcgtGAAcccgggaggcggagcttgcagtgagccgagatcccgccactgcactccagcctgggcgacagagcgagactccgtctcaaaaaaaaaaaaaaaataataatttattTTCctctctattgagaggctcatgacaggaggtaaaactGAAgtacgctataatttgggcaggacctgtattggagctcttactgcttttattttatgatgaTTCttgggggagtttgggagattgTTCttttttgtttttgtTTCttgtgtgtatctacgtgtgtgatacatatactaGAAaagctgTTCtttTTCttttggttataaatGAAgcttgaGAAgtaaTTCtgtagcaaaactGAAactaggtagagtagtcatgtttgtgacgttaaagcagccatttgccaaacctattaatggttttatctgttTTCctgtgacatgtatatttataattactgtgTTCagaggcatttaactTTCccTTCgtttgtaaaacataagagatgcttttaaatgtttacgtgccattagatattttaagataataccgctccaacttttaTTCttTTCtaagtataaagttgcaGAAttagcttGAAtactgatTTCagcttgcatGAAatGAAtatagagcagctgtccaaattaagttaaaaacaaagGAAgTTCctgtgtgtcgtTTCctgtctcttaaaagcaaacaaaaaactTTCcataattattaaggtcctTTCagagtgtctctaatattaactgtGAAttttggtatTTCagtgtgctGAAttgttggatgTTCttgctcaccttattaaggacattTTCttaaaaggtaaatgatttgtttTTCttTTCctttatcag  ***TJP1*_3’ UTR (5 kb downstream of the last exon)**  gtgctggcttggtctgtttgcccactgttTTCtagtTTCatgcagctttataatcctgttttaaaatcctgcacacaaatccctatcacccagcgtcacctaccacctcgtcgtctggtgttgcatgcaGAAtTTCtccccttggccagcatgtacagatgggtgggcagtgctcatctGAAgggctcagactGAAgtggggcaGAAggacctggagacagagtgggaGAAggcagcaggccgacTTCcccctgtgggtaaacacacacccctgcgtggaGAAacacccctgcatggggacacacgtgcgtttgtgtgtgtgcgtgtaaccatttgtatatggttttaTTCcccagataaatagcacgggcattgtttaatgtcacccacatttggggGAAGAAaatgggtttagtgagcataaataTTCcattgtggacatcctacttacttaactgccacccctgttgacatttggattattgctatagtgtTTCagtaaacacctttgtggactGAAtccatctatgtggattttatttttTTCatctttgttaaggtataatttacatagagtaagttactgtttttaaaatgtatagTTCaaattttgacaaacacatactgtttatttaccacggttaatacatacaacagtTTCtttatcTTCtctaatcatttgtacccctttatactcaacccTTCTTCcagcccctggcaggatgatctcTTCtgtTTCtatagttgcctttttgacagtgtcatataaatgGAAtcatacaatatgttgcctttgagtctggcttatTTCacttagcatgatgcatttgagaTTCatgtatgatgttgcaggTTCttttttattgccaggtggtaTTCcattgtgtggctgtaccagagcttgtttatccaTTCcccagttaagGAAcTTCtgggttgtTTCcaggtttgggagattattagtaaactgctataaacatttgcgtacaggtttttgtgtaaacgttacTTCatTTCgcttgacTTCagtttgatGAAgtctagtttaatcagtttgTTCtTTCatagattatgcttttgtcatatcTTCactaagatgTTCtatcctaagaGAAatcTTCacttagcccaaagtgtaaagacTTCtcaagtTTCaGAAttttttgttttatatttatgtctgtgatccactttgagttaactttatgtgtagtatgatataggtgttgagttTTCtgttttaaatgtgtccttattgtaaacacagacacacatatataaggcctaaataagacagttTTCatttagataaattaggGAAacaatttattgtGAAagggacacaactcagtaagtctgttttttgtccccctaccttttagtatgtggcatGAAcacctggtcaggtgtagatacctagagctccTTCctaaggacatTTCtgttgcctaaccatcTTCccacagcactgagcatggtgcagtgagTTCaTTCagatggccaagctttagggtcaGAATTCtgcttttgcccTTCccctatactaacgttacaatcaacaGAAggcttagtGAAcatccactaatgctgcgtgagtcagTTCctcctcttgacctctgagcaagtacttaatgcagtacacaaggcactgagcagatggagatgactcaggtgaggagacacagacgttgacactgggtggcTTCcgtctagacagggagcagttggctagGAAgggatgtggcagtaaatgcagGAAggccacctagcaggcagatggggagtaagttTTCggttTTCttgactcgcagtttGAAaaccatttactggatggGAAgccaggctctgttTTCcgtccaatttgcaGAAagcaaaccagagagctgtgtacacagatgcatccgggcacacgttactgcggctgcccagcagccctacatgtagagagtgacctggcactctgtTTCcaggctgcttagccgggtTTCcccagatggagtgtggGAAgacacattgttgatgtgctgatttttacaGAAccacattgttgtctcctgatcaatttgatcacagcaaaaactcagtggcatttttgactcttacctactcgtgctTTCctttgGAActacttgacagtGAAggagctgttgcccaagatgcagGAAtaccatttggtttaggggaggggttttttgtttgtttttggcagggGAATTCactagGAAggtatatattaaacttgGAAgatTTCtagTTCaaaactaaaatgcataaaactttaaatgtttgttTTCcatTTCcattttgtagataattagggctTTCtttaccagtgaccatGAAggtgccccctagtgcttgtTTCtcccagctgatgggtgTTCaTTCaggttagccaGAAgttgacacaaacaaggtgctcaGAAgagaggtctgtaaccaactacgacaagccTTCcacaTTCtgacactctcccTTCcctccctttgcaccataggctgTTCtgaggGAAttgtgtccatagttgtctagGAAaccacgtgccaataaagacaaaaattgagttttgttgcaccaagttTTCtgcctactggcgcaatgccaggcaggGAAgatgtgtaaggttTTCacttggtgcatgagaGAAgtgcacctcgtgagacgcactagagcccccatgtgGAAcgccctcctctggcactcacccgcgGAAaaatagGAAgaggtgggtgcacaggtggtgattttagcgtgttttaccttggcttgattgtcaccgtaaagttttgaggtagtttgatctatttgtccacatagcacctcctggtcttaagtggatttGAAaacatgcaggtataaatcatTTCatacccccttaaaaacagaGAAgGAAaGAAaatgtgGAAactgacatgtggtcagtatgctgctgctgtttagtgttgaggggcgtgccaagtaTTCaccctagcaactcTTCatTTCttgtcagtgggggtgtggcaGAAaccggctgGAAcgacggcagtggggTTCctagagaggactttgaggacatggggtTTCccagagcacTTCtgaGAAtgagtgtcTTCcatgttggataaggacagcttGAAgatgagcagGAAccaagtgGAAagcatGAAggttgagacccagggagcagccccagtcttgatacctaccaagcagcggttgccgttgtagtggGAAgctgagggctTTCaGAAacatttGAAaccgagtgctttaaagcaaaattgagtTTCtttgcagcctgctcTTCagtagaggacacatgcaccaaacagtcccagggcTTCattggtttttttTTCctggagacggagtctcgctctgtcacccaggctggagggcagtggggcaatctcggctcactgccccaccacgctcggctaatttttgtatttttagtagagatggtTTCaccatgttggccaggctggtctcaaactcctggcctcaagtgatccgcccaccttggcctcccaaagtgcctggattaattacaggcatgagccactgtgtccggcccattggtTTCTTCttgataggcccagggaggagggtgGAAgcttggtcagtaggatccagtgctgctgcccctgggggacaggatttggTTCttatgtttttGAAtatttaaactcagttGAAatgctcccaccaagccttgaggagttgctccccagttttagcaaccattaatgtgccacatttatgacagcacaaactgcaaattTTCcaagGAAgcatcttggacctgagtcctcttGAAacacttttgtggcctTTCcaaggaggaccacggGAAacacagggcaggcaccaggagGAAgcaGAAagtggatctgcgtccaccgtcttgacgacttagtgggatagaggcaaaaaGAAccctgtgcagggagggcaggactaaaccGAAggccctcacctcagtcaggGAAccaggagagcacagcctccagcccatcacagcccagcaacttaagtgtccaccacccaggcagggGAAaggttagtttttatttttttttagcatattTTCacgctcagttgcaacaacacagcatttgcgaGAAtaatacagaGAAcTTCccTTCtgcccttgacctagactccctgagtgttgccaattTTCcacctgtgcagtgcactctaggtaggtaggtatgtagtcagctggggctacggtaacaaaataccacagactgagtggcttaaactgagtggcttagatcaggtccaagatgcTTCcttgGAAaatttgcagtttgtgctgtcatttatttattTTCtcatcgttttggaggctaGAAggtgccagcatggtcagtTTCtattaagggTTCtcTTCctgacttgtaaacagccaccatctctgtgtcctcagtgggctgtccttgatacatgcacatggagagagcagacacgccctctggtgtctcatgaggatcctaatcctgtcagatcagtgcccacccttaggacctcaTTCaacctcagttacTTCcttagagaccccatgtccaaatgcatccacagtgggggttacGAAttttaagggatataaacatTTCtgcccataacaataTTCTTCtGAAtcaTTCgagcataaggttGAAgacatgatgcatattacctaaGAAcaagagtcTTCtcttatataactacactacacttaacctcaGAAagcattacaaGAAttgactttggcTTCatctattTTCTTCtattggctctctgtttTTCattgcgtggagtttgcgctagtctttattatTTCtTTCcTTCtacttgctttgggTTCagTTCtttTTCtaatttttGAAggagGAAgctgggtttagtgatTTCagatatttttaaaataggcattttaaagctataaaTTCcctctagatactgtttagctgtacTTCataaatgttgatatttttacattTTCatttagTTCaaaatattTTCtatt |
| ***TJP2* (ENST00000377245.9)** |
| ***TJP2*_5’ UTR (5 kb upstream of the first exon)** aaaggagagttacgaTTCcagtctactttacagtgtacacgttaactaaattgtttggtGAAcctgtaaacttTTCtctttttttttttttttgggggggggatggagtTTCactcttgtcgcccaggctggagtgcagtggcgtgatctcggcttactGAAacccccacctcccaagTTCaagtgaTTCtcctgcctcagtctcccaagtagctgggactacaggcgcctgctaccatgccagctaatttttttgtatttttagtagagacggggtTTCaccatgttagtcagggtggtctcGAActcctgatctcagttgatccatccgcctcggcctcccaaagttttgggattacaggcgtgagccaccgtacccggctGAActtgtaaacttTTCagtgttagttTTCtttgctcccagttgatgatactttataaccatattgcagatatatacatatatatatatacacacacacagatatgtTTCttaagatctccacaTTCttgaGAAaatatgactaaaaggcatttagtttatctcattaaggtcTTCtgaccaacttatTTCttattttttgagacagtgtctctctctgtcacctaggctgcagtatagtggtgcagtcatagcttactgcagcctcaaaTTCctgggctcaagcaatcctctcccctcagtcctcgagtagctgggactacaggcacatgctaccatgcccaggtaattttattttattattagtttttgtagagacaGAAtctcactatgttgtccaggctggactcaaatccctaggctcaagcaatcctcctgtctagcctcccaaagtgctgggattacaagcatgagcccctgcacctagctgaccaacctatTTCagagaccttatatattgtaatatacactGAAaagttGAAaaaaattgtccattgcacaaacaataactaaatttaaaaaGAAaaatatatgcacaagcctgtcttTTCatGAAgtcatttttttgcatactTTCcagtttttattttttttataattttatcactgtcatcatcatacattagatgtaatttgtgtttttttgttgataattatagcattatcaTTCtccttgttgtatagacTTCcttattacaattttaaatgatggtctgctatgccaattatgttgatttactgtaatttaatagcccagcccaccatcattggacattggtggtTTCcatcaTTCattattagatacaggcatatagtGAAgtgctgcctcatacctagtcatccactgtaatctaacctccagtcgtatctTTCcattGAAaagctctggccaagataagcagtggtcctccacttaatGAAcatcatcagTTCttaccttaattgacctcctcGAAaTTCcatgttgttggcctctccctctTTCctGAAgtttgTTCtTTCcttggctttttggacactctcctccaGAAgcgttTTCtTTCctTTCtggcagctcctctttggtctctgTTCtggcaactcaagtctgccccctaaatattggcaTTCccagagatgagtcctgtccttagtcctctcttggcttaatctacatcacgtctctGAAtgtgggattgtcccctcccaagacTTCaacttttatgatacattgatgatacccaaatcactcccaaatctctctcctcaaatactcatcagtgcctagcacataggagacacttgatacatatttgttaaattactGAAcatcTTCacctggatgacccatagcacttGAAaatcagtctgtaactaaactcatcatctTTCTTCaagctcctcccaagatctgctcccactcctgtgTTCtgttttgataaaagccaccgtcatctcctttaagtaagccaGAAacctgagagtcagcgtaggctcacTTCccgctcgcgtctccccatatctagtcaggcagtcatccagcctgctgatctacTTCcttagtTTCtctaGAAtctggctcccttttgtctaacactgcctaacTTCatagccacgtcatctctcacctgcaTTCccacaaaaatgcatttttggccggtcttgtcaTTCttactaacaTTCtcctcccccaccaacccctgtcccccaccaactgtcaaatccatctttatacttgcctgggcaacTTCaccTTCtggcacctcaagagtaagcctgctgtctcTTCcTTCcttacTTCtctctgtgttatttaTTCcccttacctgGAAggcttttTTCcTTCtttgtgtcttaaaaactcaTTCcgacctctcctttaGAAtgtataagcaaccccatctctgGAAagccTTCcctaagtctctcctgctagaccactctgagcctgccagGAAtctcagtgcttacacagtttatcattacttgttgattgtctactgcaatagactgagtTTCctaaggcaggatcctgagtattttatcaaggggttgacagaggtgTTCagtaagtacctgttgagtaGAAGAAtttttttttttttttttttttttttgagacgGAAtctcgctctgtcaccaggctggagtgcagtggtgcaatctcgtctcactgtaacctccgcctcttgggTTCaagcgaTTCtcctgcctcagcctcctGAAcagctgggactacaggcgtgcgccaccacgcccagctaatttttgttatttttagtagagacggggtctcaccatgttggccaggatggtcttgatTTCttgacctcgcgatctgtccgcctcgacctcccaaagtgctgggattacaggcatgagccaccgcgccctgcggagtaGAAgTTCtttggataaacacaTTCtaggagtggggtcaaaatgtgcaaacccacacgtGAAGAAgatgactaataaattgtTTCtccaaagagttgtattgatttacactgccaccagcaaaaaagctaatatatcaactGAAccgtgtTTCtaacaaccatttactGAAtgcttatcatctgtcaggtgttgtgccaGAAGAAtggagtttgtctataaatatTTCatcatTTCgagtagtgcagtccaacaGAActTTCtgcattgatgGAAataTTCtatatttgcactgtccaatagggtagctattagccacatgtggcTTCtgagcccttGAAatgagacaagtgtgactGAAGAActGAAtttgtcatTTCctctaattttagttaatttacatgtaaatgtaaacacttactggtggcaagtggtcatcttattGAAcagtgccgctctagagaGAAaaaagGAAatcagtcagtagatgtaactgccacaTTCtggattattttttaaatgttagtggctctaagcccacatcattaaggtgtggccacctTTCaagtgctatcatttttattagtcagcaccacctaatttggGAAatattttatttttttTTCcttttttttgattaaaacaatttttaaaaaatagagatggggtctcactatgttgcccagactggtctcaaactcgtgggctcaagtgaTTCtcctgccttgccTTCccaaagtgctgggattacaggcatgagccaccgtgcctggccagatttggGAAacattttataaataggtgcttagatcaggagagcaatgccttacatcagGAAtacaaccaaaatgtctaagtccggagactcgaggcaaagtggGAAacgttactcacagtgctttttatccagaggcgtggacttagttactccaaaggagtccaGAAgcataacacTTCtggagaggcatctactggacttgtttTTCcacGAAacTTCtagacttgttttgatcaggcagGAAgtGAAgcaacttTTCagggagagagcccagcatctgcatctcctctgggacagaccacaccttttaccTTCtacttgtgagcctaggacgagttttgctTTCcaattaaggGAAgagcagggcaggcaaaactaTTCtcttacaagatggttttttaagatTTCaaacccaaggttatatttttaatcggtacctgtttggaTTCtccaaattaatttatatgctcttaaaataaagacacttaacgtcactgcacaaTTCtttgcacatggaggacTTCGAAagGAAtaagcacactGAAttgcattagatGAAtcctgtcagataagcacttgtgacctactggcaaccttttataagctgatgatgggagaggagtTTCaatctctttattggagttgttgGAAatgtgccaaagagataacctgGAAtatattgtcctGAAaagtcctgttttaatatttaatacGAAcatcttataaccctcgGAATTCgggtaggcagttgttgggctgaGAAatgtggggtacaaggccgtgtatgtgttggttagccgggcaggcctctagacaggacctaaaactttgaggttTTCccagTTCgactcTTCgtaggaGAActGAAgctgcctgtgtgaggccctgcGAAcctcgccaaaaagtcTTCctacaggatgcccccgcgacctcaccgggGAAacaccgcggacagtcgggccagcagcgcccggagctcactccaggtctccaaacttgcagcacTTCccagagcgcgcgcgctcggagcgggacctgcttgctccagtgcacgccgcggagcGAAccatcgggctggcgggggtggaggggtgcaggcgtgggggctgcgggccgctcgggccttgggcTTCtcctgccgccgccgccgcctcccgcccccggccaggagtcgcgcgtgacgcggTTCgccgcaggagcctcGAAggcgcggcgccggcgagcccTTCcccggcaggcgcgtgggtggtagcggccaatttgacagtTTCccgggccgggcggccagcgcggaggcgccacgctcgggtcgggggcgggctgacgccgccgccgccgcgggaggagggacaaaggggtgggtccccgcgggtcggcaccccggcggttgggctgcgggtc  ***TJP2*_Intron 1**  gtaagtgcctccttgtgccgcgcggttgggaggagggtcgtgagcgtgagcgtgggagcgctgggggctctgctcgcgtgctgctctGAAgttgTTCcccgatgcgccgtagGAAgctgggaTTCtcccatccggacgtgggacgcaggggaggggtaggtTTCaccgtccgggctgatgactcgtggcctccggggctcctgGAAcctttactctGAAgtcacttgggctggagattaaggcgcgagtaggggacactgtcttGAAcccggtgtcctggatTTCtgctggcttggaggcatctcgtgtctgttgtgtcgcttgcatgtctttgtgttTTCcTTCtctatTTCaaaccacactcggtattgatgcaaagttacaggcttgtgtacgtgagaGAAactGAAaactcatcacagagcaGAAtcccttTTCcctTTCaatcagctgcgtctgtaataGAAgtaaaagttgtttttttttttttttttTTCcagtaggGAAtggggatggaggtttgGAAggacccacaaaagaggagtgtgatGAAtgctttgtaTTCtagtactagcctacctgtcaccagTTCtttttatgtcTTCatttgtcaagtTTCtgcttttacacggtagtttaagttataaataatcagggatTTCtaccacagggttttaacaTTCttGAAatgtggacaaacttgggagtgagtgctcccgcgcttagtaatgGAAtacagcctcccacctctatTTCagcTTCagTTCttaggctaggctgcttTTCtggcagctgtctggcctgagtaatgatggttgtggtTTCcctGAAgTTCccagcggagagtcatcccatgataGAAactgccttgcgcTTCtcactcacgctctcagcccgggGAAtcccagcggggaggagggagggaggtcgttTTCTTCagctccccaggtggtctgtgctgggtgtgctgacggtccttttggGAAaacaggtccacctttgccagcgtaaTTCaGAAagagatgtaattTTCtgagagcacacacctgggcaggagatcgcttGAAgtgaGAAaTTCgtagtggtaaatgtatttgtagcaaagtaccactgcatatcctgcttgagaggatGAAatttgattatttagtaagttTTCatGAAcaactccacTTCagtgtggggattacgcagatgagcaagagcgtggctctgtcacacagtcccgttggGAAagcctgcaactcattaaGAAtccgatgagtgccatttaGAAatgtggacagggtactggagcTTCctcattgtggggagatggcaGAAgcggaggtgctttactaaagcaccggtggcttttgaggtgtTTCttgtgtttttTTCaGAActacTTCtgcagtggggccactttatTTCttTTCagagctacttatgcagattgatGAAgggGAActTTCccctctGAAatgctgctgtaactttTTCagtaccatccagatattataatactGAAtgagcccctactcctgccagtcactgtgggggatacaaagatGAAcaagaTTCaatTTCtgcatttaggggtctagtggacctGAAactgttagactttactctgcaatctgtgtcgggaTTCctgagactgagggaggGAAtgtatgatgccgatgcacactctgcaatacaGAAatttagcaaagagtgggattggtcctatatgcaTTCccTTCaggggaTTCttgctggGAAtaaagggcatttGAAcagttacctcacccgagcaagccactataGAAaatggggccagtaatgcgcccacctcaccgggctgagtgaggtctGAAtGAAatgacagagagtacaaggcatgtggtggatgctacgtaaatggtggtagctagtaatagGAAcaaaacgggtgctGAAaactcttaagtgttgggtcctgggttgataagggaggtgagatctaaagatacaagtaggaggtgccaggtaggtgtccagGAAaaaactGAAagagttgttgTTCacactctgttaaaaggccttGAAggccataacGAAgctttgggtgggggGAAgtcaGAAatccctcatgtattaaatgacttgactTTCcctacaaaaGAAgcGAAccagtTTCccagtaaacatcccaccccataactatttttgaggcaTTCataggcttaagaccaatGAAtttttacatcacccccggggGAAttgcatttgcaatgacTTCtgcctacatttaacttttgatctagTTCtttgtacactgacaaaagTTCacagccaaccatTTCtcTTCaggacaagtgtgaGAActTTCtgcatactgctGAAggcaagggagattttgagtttagcaaggttTTCtctccttaaGAAggttatctttttacgagacacaagttattGAAactaggcggggccactttagtcctgtgtacaataatatgtgtagtaatataaatagtaatttaaaaagtcatgcagacttggttggggagaggcatGAAgtttgggtgtggtgggggcggggtGAAgcggtaGAAgggTTCgcaatcactgctGAAactTTCaacataTTCtaccaacaatatgagccaccaggGAATTCcttgcctcatctctaGAAcGAAtcccattaagcagttgtggctacagatGAAtttatctaggttgagagctgaTTCccaataacatgcctcattgatttgactctaagcagtaatcaggggcccaacagttgttggaccaaccagccttgattTTCcagaGAAggtgatttggtatatggtagtgtctgagggtgagattgccccagatgggagtggaggGAAgcaGAAcagttTTCtctggtgttttactgagtttttgtcttgatcctaaatccaagggttagggtcaGAAacacagactcagaggttagatttaacacccatttagtgggttaaacacatcaatggttatgtGAActttgttaaggtttaaaaagctatttagggcctgtTTCtccctcatagGAAtttttgcaatcagcctttttagtagatgcccatataGAAagtGAAtttgggagggtctaaggtggtattactattaaactttttGAAaaggctgtgtgatagcaatgGAAaaaaaaaTTCaggcatgGAAatccaatatgcagtattgcagtagctagttttaattttatttttgtttttttggttttttttttggagatggagtcttgctctgttgcccaggctGAAgtgtggtggtgcggtcttggctcactgcaacctctgcctcccgggTTCaagcggTTCtcttgcTTCagccTTCaaagtagctgggattacaGAAgtgagccaccgtgcccggccactagttttaattttggatggtacctagGAAagcagttgagctagtatgctgcatacattgtagtgtgagaggcagagTTCcgagttggagagtgtctgcTTCcactttTTCcatttgattagGAAaagcacTTCttaGAActgggtgactgacagctagagccccaGAAgacctgcacaagctggggcaatggctacccataacagtgacggctccaTTCtgttttttaaagggattggtgtctctatcatccagctggccattaaacaaccaaagcTTCatcatcctagataacctgtgagctctcagaggagacagagtaaggactatcatacccccaaaatacctaatcatttaataaagcattTTCtctaccttttatgctatcccaaaacagtgctTTCcaaagtctggtatacagacagacttacaggtggtgcagagagatacataTTCtatttTTCaattatgatgcatGAAtattatatgttagGAAtaaaaacaGAAaaccagctaccacatccatttatTTCatggctagattgcttaGAAtgaggcaaaaataaatattaagtaaagcagtgagtttgctttaaGAAaaattatcaactctggGAAtgagtaatatgaggctaaggcaaaaatggtaaagctagcccttgacatctgaggtgtgcataatactgcttaaaatatgagtgtggtgcttacttatagagagcctggtataccttgaccacgccTTCaaGAAagtcaaacacaGAAtggcccaGAAtagccagGAAagttGAAtttgatggactTTCtgtcttaagcgtgtgcatcctgctcttatccactcagatctcttagatgtggttgcagacagtgtatttgtagagctagctgtttgttttaacgtctggcccaaTTCtcaTTCctTTCtctgcTTCcacaagccaTTCtgcagtttttaaaggtctgttggtatgatatttttgatatgatagagtgataggggagGAAgtattttgacactgatGAAggacatcactgtattatcaagtgactcttgctcatgaTTCattgccGAAGAAtgtggacagccaaacagcctcacctgtaaatgcatttaatTTCtcaaagtctTTCaGAAGAAtgccaactggatgagtttttttTTCttattttttttttaatatGAAgcttgtattggtctgtaaaataggctttaacaaggattTTCgtagGAAcagGAAgccttgaggtaGAAaacaagtatccctgctttgacctagagagatgcatgctcatggcaggcGAAtGAAtcaaggaGAAgaGAAaatactgtTTCagtagactctctagtgatgtgtgtgTTCacaccatttatgaGAAatcctaagctTTCaattagtaacttgtactttagtcatttaaaatcagcacagatgtttgtttagtgcctctgccagatggttaaagttaaatcaggttttttaacctGAAaaacgtaatgtagctTTCcttaaccatttacacaccattgtatattgcagaccatagtcagatacattttgttataatGAAtgtttattttaccttttagctcaattaGAAtatcagtggttatggtTTCtattttgttTTCctacagttactgtatgggggtgcagtaacagcggtggtggtGAAagtaagttacttttTTCtttTTCtTTCtttttttttttttttgagacggagtctccctctgtcgcccaggctgGAAtgcagtggtgcgatctcggctcactgcaagctccacctcccgggTTCacgccaTTCagcctccccagtagctgggactacaggcgcccgccaccacgcccggctaattttttgtatttttagtagagacggggtTTCaccatgttagccaggatggtcttgatctcctgacctcgtgatccacccgcctcggcctcccaaagtgctgggattagaggtgtgagccaccgcgcccggctGAAagtGAAtctcttaatggatcTTCcatGAAtcaaatatttgtgttttagtaagttaggctgaccaaaagatTTCGAAtcatacctttaatcatggcaaggatcttatgtcacaTTCttgagtacacatgTTCactaaattttacctatatgtgtatgtGAAcatggacTTCaGAAttaattagtgtggtaattattatTTCtattgtagactttaatcagccatattaaatatatgcattatgTTCacagaGAAaacatggtatcatctttgGAAactttacagtatgctctatattatagataccctatataactatggtactaaacacacgagttagtgacaagtcaggcatttggaggGAAtgtttgtaacccattgtgggagagagttattaGAAtagttaTTCttactggtaggtaacagttatggctgtaatGAAtaGAAtgtgactaaaatccagttatgcctttgccatcgtattaaaagctttggtggagacgtgaGAAcattgcagatactttggattttgtcacttgagttGAAggtaaacagaGAAaacagtactTTCtagtcatcagtagGAAttaactcTTCcaggacatgtagcTTCaaggaccTTCtaggTTCTTCtgaggggGAAataatgTTCcacttggggccatttttTTCcatttgttgagagtcatttgttTTCagagtattaaagGAAgtgTTCcgagTTCtaggttgtgtTTCtttgatgcctactaaagctgagcacacctgagaccctgggtggagctctacaatggGAAtaggagtaagtaatgtaggcTTCgagagacaatcaTTCtgctctcagtccttgGAAgactgTTCctaggtatatatggcaaagcagatGAAattatttgaggGAAttaattagcaaaactaaacatTTCtgtttttGAAagGAAtGAAtcatttgGAAgGAAcTTCctggtgagctcagttttagcagcctggtTTCtgcgttacccTTCtgcagtctggctGAAtcaattgtgTTCttgcctgtgattggtgaggatgcacatgctagtatttaTTCtagcccttatcacTTCccatcagccctgagcccctccctggtctgagtcatcttggtaaccccaGAAgctaacagagcaccaacacatccactaaacgttggactgacttgatttggggcaggcttgttgtataagctagtagGAAtaagagactaattgaGAAattaggacagtggaTTCctgacatttatatagtgagtagatgatcgtttgttaGAAttgcGAAtTTCatccagaGAAttttgtccaaaaaaaattataGAAtTTCatctagaccatttttgtaatggcatgGAAgcagcattagctgcaccactgattagctgggtggcttttgctgtcttgtgactctagtttgcaatTTCttttttttttttttttttttttttttgaggtggagtctagccctatcgctgaggctggagtgcaatggcccgatctcagctcactgcaacgtctgcctcctaggTTCaagcgaTTCtcctgcctcagcctcccaagtagctgggattacaggcacacgccaccacaccctgctaattttttgtatctttggtagagacggggcTTCaccatgttggccaggctggtctcGAActcctgagctcgtgatccgcctgccttggcctcccGAAgtgctgggattataggcatgagccacctcgcctggcctagTTCtcagtTTCtttatcaGAAaatgagtagttacagttgatcatctcaaagtcttttgtgcttaagaTTCtGAAggttttGAAgtttgagtataaagcctagaGAAcaTTCtaaattatTTCcattggTTCTTCtcatcttaaaatgtttttgtgctccttaaactacgtttgtgtttgcttgcttgctTTCttttttttttagagatccagtggattatctctGAAaagcattttttaaaaagacagagtccaagagtggctggtagttgttttgctcataaagtgatgGAAagatctattaTTCagttgcagcttaaattatgtaccagcaccacgtgtgctggaTTCtcTTCcttactttgcagcttaagccagcaactgcaaccatcttgcagagcTTCttgaGAAtccTTCTTCctcTTCctcTTCctctccacccccatggacTTCtccccgggcctcattgcctgtgacatacgcttgGAAggaggggcaggcctgcagctgggagccctTTCtgatttgTTCTTCacaaatgtgttGAAgatgagcctactggtcactgctgcaaaaaaccacattgTTCactggtgtaaaatcactgctgtcatctgagtTTCagcataacTTCaggcatgttggagtttTTCaacgtcacctcaggcagagcactttgccTTCcttgggctggtctgtttagtgtctgggtgagttttaagagaggagagacattgtggcatcttagacactcactGAAattTTCcaGAAatggagctgGAAacatttgtgcgtTTCcTTCtggtgttattgttaaagcctaaaaacaagggcaagtggcaacacattGAAatGAAGAAgccccaagtttgcaacacattGAAagGAAgaggccccaagTTCgttttgagcctaaattGAAttttaatcaaaggtctgagaGAAatatttagtctacttatactatttaagtgcacagtttggcagatgactttgggcagtGAAggatctgGAAgaggcagGAAaggtagactcaatctgccagacttagagctaaaaaaatttgcctggtatgtTTCcatGAAGAAaattTTCctgtaGAAgcagtggcacgacctctgttacaagtctgcttattgataatGAAgctttTTCtgtgttatcaatTTCgttGAAtttatatgtatgtatatacatatacacacacacaaatctcaagtagagagcttgatGAAtTTCtttttttttttttttttgagatggagtTTCactcttgttgcccaggctGAAgtgcagtggtgccatctcggctcaccacaacctgtgccctccaggTTCaagcaaTTCtccggcctcagcctcccgagtagttgggactacaggtgcgcaccaccatgcccggctaattttgtattttttttttttttagtagagacaaggtTTCTTCatgttggtcaggctgatctcGAActcctgatctcgggtgatccacctgcctcagcctcccaaagtgctgGAAttacaggcgtgagccattgtgcctggccaggGAAttttatttattgtggtaaagtacacataacattaagtgtaccattttaaccattttaaagtataaaaTTCaatggcatttaatacactcacagtattttgtaaacaccatcactctctagTTCcagaggtaggtGAAtttttacatGAAtgtaTTCacctgcatgattaccacctagattaaaaactaGAAttTTCcatcactctgGAAggTTCtctcttgccacttTTCcaatgattaccacacctacctaaaagaGAAcTTCtcTTCtgacTTCtctcaccgtgGAAcacttttgcttGAActtgggagaggacccatatataaataaaatcaacattttatgtctgtcatctTTCactcaacctaacctctgtGAAaTTCatccgtatttgttgcgtgagaTTCagtagTTCttttttgccactgagtcgtaTTCcatttatatgcatctgtcacatttggcttatctcTTCtgttgataggtatttgggttattTTCagtgtttttgctattatGAAtaaagctgccatGAAaagcacctacacattgccttgtggacatgtacacccatTTCtcttgtgtGAATTCctaggaccataggttagggattttttaaaaaacttattttagattaaaaaaaatattttttgagacagtctcactctgtcacccaggctggagtgcagtggcatgatctcagctcattgcgacctgtgcctcccaggTTCaagtgaTTCtcctgcTTCagcctcccaagtagctgggattacaggcatgcgccactacacctagctaatttttgtatttttagtagagacaggtTTCtcTTCaactcctgagTTCaagcagtctgcccaccttggcctcccaaaatgctgggattataaatgcgagccaccacgcccagccaggttacagatgtttaactttgaTTCtttacctctGAAgtcaaataaacctgggtgtGAATTCttttgtcctgccacttgcttgcttgactttgagcaatttacctttTTCtcatgTTCagttTTCtcagttgtaaaataggcctaccctatcagtcatggTTCagtcaaGAAgacatgtcaTTCtagctatTTCaaggaGAAggggaTTCaatacagagtattagttgccTTCaggacaaaGAAaaggcgagatgcgcaaaaaatgGAAgcccaccgctggctTTCtgatttgccgcaaaaaaagttaGAAagctgctgccacccagaGAAgactcaGAAggtcttgcttgaccTTCcaccTTCtcagcatctccagccttgaggctggtGAAagggcaggGAAtctggacatgctgtaaagctcatgtctgagGAAtccaacttttgcctgctactgctgctgtctgtagGAAGAAGAAaaacaaatggcTTCtgctTTCcTTCatccTTCcaaatctcaaacacgtgtacctcgctggtaGAAccctgttggcaagagagatgggggatctctggtaGAAacaGAAactcagtgccagcaaaGAAGAAatggcatagatgccacctggggttgctttgaggtgctcagtaaatggcagtggttataatttattattTTCatcaTTCctcaaGAActgctgctgcagTTCagactgatTTCtctctctctctcTTCttttttttttttttttttgagacgggcttgctctgtcacccaggctggagtgcagtggtgccgtcacagctcactgcagcctcgacTTCcctggctcaagtgatcctctcacctcagcctccgattagctgtaaccacgggcatgcgccaccatacctggctaatttttaaatattttgtagagatgaggtctgtctatgttgcccaggtgggccttGAActcctgggctcaggcgatcctcctacctcggcctcccGAAgtgctaggattataggcgtgagtcaccacgcttgggcagcagactgatTTCttttttttttttttttgagacagagtcttgctgtgttgcccaggctgGAAtgcagtggtgcaatctcagcttactgcgacctctgcctcccggaTTCaagcaaTTCtcctgcctcagcctcccgagtagctgggattacaggtgggtgccaccacacccggctaatttttgtatttttagtagaggcagggtTTCaccatgttggccaggctggtctcGAActcctgacctcaggtgatctgcctgccttggcctcccaaagtgctgggattataggcgtgagccaccgtgtccgacccagactgatTTCtgtaacggatgTTCaTTCttTTCactttatagtcacatggatgccactgtaagagctccgtgtcacataaggtgttgtttTTCtgGAAgggccTTCcagaTTCtgtatctattgctctgtgaTTCtaggtacttTTCtaaaggtataagcttgaGAAattagttTTCaagtctgtaagttaatgtagagctGAAaggtgacttaaTTCtaagagttgctgctttaatgtataattttatgGAAcctaatgagtgttTTCtttttaaagtcaTTCcactttattattaataaaataaaaggtaatatataaatctggctgatagatggggttacttttTTCcctgtctttagcTTCctttgtgTTCtgataaatgagatgGAAgatttttatcatattttgtacctcaaatctctcgcggaggGAAcactgtagtctaccaaaacagctggcaGAAgtgtctgtcctattataagtttattttatGAAacatgagctataatgtagtcctttttttttttttTTCtttttttggagacggagtctcggttgcgcctggctaattttttgtattttttagtagagatggggtTTCaccgtgttagccaggatggcctcgatctcctgacctcatgatccgcctgccttggcctcccaaagtgctgggattacaggtgtgagccgccgcgcccggccaatgtagtggttttaagacatttaaaaatcttGAAccaatgttggcaacagcctgtctgtaatgatgtcacatgtgGAAaGAAccgagtttTTCttaactttaGAAtgtGAAttttTTCctccTTCaaatactttTTCctataacccagacctattttactTTCcagggaccctactattactgccctaaggtaatttgctaGAAatggagtggtcaagatgtattgattaacgttttaggcatacTTCtgtagtactacTTCcGAAggctccttgcagaGAAatttGAAgtgataagGAAggcaaataTTCGAAatgGAAagataagtataaccagtgactatTTCagccactggtttagcagTTCaagtgagctggctgtttgtaaagcttgcttagtaaatGAAtgtgctaGAAatatattgacaGAAtttggttttatctctttTTCtccagacttttagtcggccagggctaacaatggatgtccaaacagtttacagtggctgatacacaatgcacaaagggcTTCactgtttggagactgGAAaatagagccagaggggagcTTCttatgggcattgacctaaTTCactccttttggcaaaaatgcttggttttaaatcaatgtatgtatTTCtgacccaaatatctataaatatTTCagctagtagtgttacagctggagGAAgggacccgtggtgttgcTTCgtgtattttgatttgacaataTTCtttaGAAataaattgtcaagtGAActaatattacacaaaagttaggGAAagttaagagaggcagctGAAaTTCcatcagtTTCctccttagtccctgGAAcctccccacaatcagGAAtttgttagtcacctggtttttaaagataaatTTCtctgtgggcgtTTCtgctgaGAAatgtgggGAAacaggactgggctaaaaataaaattagTTCaggcattgctaaacTTCtaagtatcatgtttgtgatgtacttTTCcagGAAacataaattggcaatatttttgctcctttttactaccacaGAAaGAAtatttgcagtcagcatgcacggcctGAAaGAAtctctctcgcctattaataGAAcaGAAgGAAcatacacatgtctatgtggggGAAaaacatctcatttggcttagtagtaagctccaaataacaattgcattgttgTTCtGAAttgtctttgagtgtgattggatgattaaatataaatgttgacctgattggattatatcacagcaatGAActgtcctGAAaaaacagctgtaacataccactccagactgttaccagacttagtcggctggctttatatttgtgttgtcaaaactgggatgctgtcatTTCtgtTTCtctgtctctTTCgtctgTTCcaatttgagatatactgactatgttagtgtagtctgctacatggatagtcctttatctTTCagcaacataagcaacataagctttttgactgagacgagcgtaatcaaaagGAAtgctggGAAacagtGAActgtaaaaaatgtcctcaattagatacaggcTTCagtgcatgggtttatcctTTCaGAAgatcggtgtgttTTCagtgagggtctaaacccactggcctggaTTCcacctcttgttggatgggtggtcagttttaacattagggggtgttgattgtgagctcttgagagcagggctggTTCtttgcaTTCctaggtaggcatgatgtagagtagttgctcagtaaatagggGAAggtGAAagGAAgaGAAgggaGAAggtgagtctcaaaagactgGAAtgtgataagcacacgtttatcacactgggggtgtaTTCtctcccttTTCttagagtaTTCcttgcctcctGAAaaaagcactttgcagGAAatatggagatcTTCtGAAcctgtggctttgtgttattggactgaGAAgtccaaaatgtgcattagcggagcttataGAActgctctgaGAAtgtgTTCttgagTTCtacaaatattatggggtgcgttgggggctggctcactccagctcagTTCccatgtgttaagtgGAAcTTCtgatctgttGAAtgcctaaccatgacggagcccaggagacaaagtaaggtgtggtttgttttgctgtaagaccacgtttggttagcttTTCtctagcaaagctgggGAAggagatcacagggctgatgtggtggtaggcccttggtgtagatctcactgacttTTCccagtcaactaggagtaaatgttaaccctattttataggtggGAAacggaggctcaGAAacattaagtaaattgcccagtgtcacacggcttgtgtgacagggaggggatttgagccaaggtccatttgatGAAtttGAAacctgtagaTTCtgtcaaTTCcaccagtctctgctttttaGAAtcacagtggcaaGAAaataggaGAAgctcaaggccctgtgttgattattaggagtttaaacatcatcgctGAAagtgcataGAAatatttgtaTTCctagaGAAtcttaagtaaaagtaacatatcaatgttgtcaagagatgtcTTCaTTCatcagagacctctgtgtgTTCattactgtatgagaccccaaagactctaagttgcctctaagacacagccttacagggaggtattaagtgattatgtatacagctagctaaaaccacaactttattataGAAatattaaGAAtatgtcttatcatgtgagagtaTTCtTTCTTCaagtccatagcctccagttaGAAatggctgtgttgtcccatcaaccccTTCactcaaaaactatcattaatatcagGAAaaacttatggttacaaactcctgggttataGAAttgGAAgGAAaaacttagatatgctgtgtttgggagcttgccttgaggattgGAATTCcattgcaagttaccttggggtcaaagtacatcccctaaaacatcaaatacctccaagaggttttaggctatcagacagccacactttaatgtggagtagactagtcagctTTCTTCtactatctaactgacaccatacacattgttaaagagataattttgattggGAAtGAAgataggGAAttttGAAgccaGAAcaTTCtggtgatggagcagatactgccgagatgttggcctcgggacagcctggaggaggaggacactgataaacagagcaaccagcccaaggggtgGAAagacctagGAAgtctccacatacaaattttgcTTCtTTCctgtTTCtccactGAAccctggGAAgggtgcttatTTCtctcctcactgtctTTCcccaggacccaGAAttgataggttagttgagtTTCtgtgggcagtctaaggtagtgttactatttaaaatgttagtaacattTTCagaTTCactgagaTTCttgctgagtgtggtggcatatgcctgtggtcccaactacttggagactgaggcaggcaggaggatcgcttgagcccaggagttggaggctacagtgagTTCtgattgtcctgtGAAtagGAAtagccactgcagtccagcctgagcaccacagtggagactccactttttttttttttttaagtttaaaaaagttaaaaaaaaaaaaagacaaaGAAaaagGAAagttgatttttggTTCtaactcagttgtgacaaacggtggatatatggTTCtgaccacaTTCtaaattggacttgatggtgggcaaggGAAacTTCcaatgcctatcccctcactttaaaaaaacTTCtcctgggccaggcacggtggctcatgcctgtaatcccagcactttgggaggccgaggtggacggatcatgaggtcaggagatcgagatcatcctggctaacacggtGAAaccccgtctgtactaaaaatacaaaaaGAAattagccgggtatggtggtggacgcctgtagtcccagctacttgggaggctgaggcaggaGAAtggcatGAAcctgggaggtggagtttgcagtgagccaagatggcgccactgcactccagcctgggcaacagagcgagactctgtctcaaaaaacaaacaaacaaacaaacTTCtcctgtagcagtttgctttgagttgtgggtgccacctccctcactgtctttTTCtgagggtacataaataaattgcatcctagcttgagatcaggagcaatggtttttagTTCtctgtgtccaacacaaTTCTTCtacacagtgtcttgcccaatacatgtgttggtaatactgacaaacatagttaatcTTCTTCtttGAAgattatctGAAgcaagattttgaccttaggtcagcTTCccctGAAgtatgtgattgGAAactGAAcgagTTCagattttagtttaggtaaaaatataaccagaGAAgttgctaccaagcatagttgactttgagctgacctaacttttggattatgtactcactaaGAActtacctcttattaattaaacaaatacaagGAAataaataacccaataaaaacactcaaaacaagtttatccccaaatagtgtacttgatgctaaggacagacctaaagtttgttgaggataGAAatGAAaaatgaggcaaaaGAAaccagGAAttgagatgtgtgtgtgcctgtaccagcacccgtgtgagccccttgcaggtaacgacccatggtccctgcacatcagtcTTCtgttactaagttttgcataaattgagtctggtggttggcaagctacatatagccagctgcgtgtttgttgttttTTCtgcctgctagctaaGAActgTTCttgcattttaaatggttatgtaagtatctatatgatctccgtgattttgcctcttgtagtggcaagcctaaaatatttattgtcaggccctttgaGAAaatgtTTCctggctcctgcctcagacctcctgGAAgcagggtgttggcttgatttgtctcctgTTCtgcaatctggGAAtagtagatcaggatgctgacttgtggGAAgatgctggctgcatgagatgcccctgtctgcagtcactcatagcttgGAAaagcaaaaagGAAaaagggttggatctgagcactgacagacaggtttgcTTCtggtTTCaatgtagtaaggtgacctgataacaagggccacttgaGAAgttgcaaatgcagatTTCctccgtgttaggtggtgaccaataaaagcatattttgtttttaggatgtaGAAaggctataaaactgcaatgtctttgtatcatcaaacatTTCtgtgtTTCtgatgtTTCagatgTTCctatTTCtctggattgcaggatTTCaactttgcaacatgtactgttgtgtTTCtgagttgttgactatTTCagTTCcaaacagTTCgttatTTCctgtcagagGAAacTTCagagtaacaataaagtgctggtgtggcTTCaaaggcttggaGAAatGAAaagcagtggaGAAattttgtgaggTTCctttggcttaGAAggTTCaGAAaggctttgagcGAAtGAAtgtatgtatcagtcactgttactccaggtccgttagctgtTTCcagacctTTCtgtgtcctaaaaacaaacatcattttgagggtggggactgtgtcctatgttgacaggtctttgttTTCaaagtctgatgtacaatagagactaaacgTTCaatgactGAAtgcagTTCatttttaaaatttttttttttttgagacagagtctcactctgttgcccaggctggagtgcagtggcatgatctcagctcattgcaacctccacctcccaggTTCaagcgattTTCctgcctcagtctcctgactacctgggattataggcacgtaccaccacgcctggctaatttttgtatatttagtagagacagggtttttgccttgttagccaggctggtctcGAActcctaacctcaggtgatccacctgcctctgtctcccaaagtgctgggattacaggcgtgagccaccatgcccagccgtatgttttaaatcttatattaagcttataccactgttgattgccgttgctacagtggggcttatattaatattaGAAacacccctcatttgagtggtactttacagtttatagcaacctctacataaagctgggcatggtggcacatgcctgtaaTTCcagctgcttgggagactgaggcaggaggattgctccagcccaGAAgtttaagaccagcatgggcaacatagcaaGAActtgtatcaaaaaaaGAAaaaaaaaactTTCatgttaaaTTCtcacttgatcctcaccaaGAAccctgtgaggtgagtgttaTTCactggatgcttagaGAAcctagagatcatacagctggctagtgcgtaccagGAActtGAActacttataaactggagcagtcggaGAAagcttggtgtgtttgacattgtctgtctcttggtctccaggccccaagagGAAgcaaaagcctaaTTCatcttggcaaatgctgtaGAAgTTCttgcttggattTTCtTTCgctagacagtttttggtggtggtttgttgctctccTTCagataaacatTTCttgatagtgcagtTTCTTCcTTCaatatgTTCttaTTCtggtgtgtacatccgtTTCtcaGAAtgtttagtaaacatTTCtgttgcataccctaatgccagtTTCttacctactcctcagtagcgtgctgcagcagtggctttgggacctggtgtagacattatgggttgctttaTTCaTTCctcacagtgacaaggggatcttgattggtgctaagtccttggatggTTCccttggcctcagtgactctgagagagccatcactgcaggtgtgatcagtgccagggtcTTCctTTCTTCcTTCtctctcactttttttttttttttttttgagatgtggtctcactctgtcacccaggctagtgtccagtggcacagtctcagctcacttgagcctcctgggctcaagctgtcctctcacctcaatctcctgagtagctggcactacaggcgtgcgccaccacacctggctaatttttgtatttttagtagagacagggtTTCaccatgttgcccaggctggtcttGAActcttgagctcagacgaTTCacccaccttggcctcccaaagtgctgggattacaggcatgagccaccgtgcctggcttttattgtaatttttaaagtctatTTCtaatTTCatctaaattatgttttGAAattTTCaaTTCgtaggGAAccggcagtctctccctttttatTTCtgcatgactgcaccacctagtggataatacatctgttaccaggcagtggcctttgatgatTTCctatagctacaaaTTCtacaaaaagGAAaattgggtgcctttttatgctttGAAatcacatataatTTCtgccagtaatttttgtTTCtaggcaagtacggactttttgagaggagcttaataacgtttaaTTCaGAAttaaaaaaaaaaacgtcagcccatacctGAAgtTTCccgtgtcaccaatggactaaggatgcagactgtcagtTTCactcagtttatgtgataatggcatacaagataGAAtaatgaggtctttTTCtaagtttaGAAacaaggctatTTCaaacacctGAActttttttttttttttataacttgcttGAAtTTCatctgctgtgttttattaagtcaaaaGAAttagGAAggcttgttgatttttgcatggctttggctccacatttgatacagGAAacctcacctTTCccctccctgtctcttgtctttgctgccccctgcctggatggagtatgTTCcagtatgatggagcaattgtTTCtttttgtctgttgatTTCcattaggtgcagtgggcattggtcattgtgctaagtataccatgcaaGAAcacaTTCagttattacgtttatggtattttaaatatgtaacatacttatgcagttGAAaccataaattgtaagatttttagtcttgtccatagattaacggtttaaaataaaattattgtgtctcttactgacagctggttaaatcagtattTTCaaagtatcaatttactaagttttgtcatgtgcctctataagGAATTCacagtcTTCctctgcgctcccacagcctttttatttTTCcTTCccagGAAaaattttagagacaccaaaaggaTTCttaaagtataaaaatGAAtTTCactgtttttGAAaataagtactatttttTTCttaatacctggcaTTCtgctcatggttgcagcactgtaaattgctGAAagttataggtcattacacatGAAgaggcttaaaaatgccgtttgatctatagttaactacaaaatatggagggggagctatatgcacacagtgtctgccatagcattagttatactagcaaaaaaaagGAAatggcttaaatgactgctagggatatggttaattatatagtaaatattgctatttacaaatttaaattaactaaaattaaacattgcaTTCTTCagttgcagtagccacatTTCacatgTTCagtagccacatgtggctagtggttactgtgttggacagcacagctataGAAcatcgtcaccatggtaGAAacTTCtgttggagagtactgctagacaggtgcTTCtcaaacattattTTCcccgtcacctccctgatgggaggtgcTTCtcaaacgttaatgtgcatattaatcacataggggctctagttaGAAtgcatatttttatcagtaggtccaagttagggcttgagaTTCtgtatttttaacaggctcccaggggttggctacgtaatacccaaagggtggggTTCttgcctttaTTCtccaaTTCtaatagGAAaggcatgtgtttGAAgctcaggactccgtatgtaggctcactggctttatgggattaaccaggctcattgctaggtggtactaatgtgtccctcttTTCtgtggGAAccatcatctgatggtgccccaggtctgggaggGAAcgattgaccttgtgtgtTTCctatggggccaaccttgatggtgacatttagctgagcagctctaataattgttttTTCaacctgcctttttgacaaagccgtttatgattatctttatcttggtgcctattatgggggtggtggtttgtatgtatgtttgtttttttaaagtGAAgctatgttttagtgttatataagtttagtcaggtttaaaattatagggtacacagggctgtgcctgtgttgccaaattagcaccTTCcttttggcttattaatggcTTCtttgtaattacaaataaGAAtgatctgtttatagcttagcttagtgtatcaGAActagGAAcatagaggtcttaaatgtaactatcagctgatttttttttttttggtctcatatcttatGAAaattggactcatgcaaatttgcaTTCaaattgtgaccaggcacctgttagctgtggggccttgccatgggcaagTTCcttgagatctcaggccTTCtatttttgtatgtGAAaaaagcaggagtagggttagggtatagggtttttggaggattaataggcaatagccataaaatacttagcacggtgtctggcatattgttaatgtccaatcagtgatgatgtgtTTCaggcccccccttTTCcatggtgTTCacaaaggcgtaggcattagggagcttgtgcatatataccaaagctgactgTTCaaacagccaGAAataaaatccgactcgactccctTTCtgcaccctcTTCttaccaactcactgcaGAAgacttattgggctgtgttgactggacccaggccagtggtgtgGAAatagctatcatgcttgtGAAgatgTTCtgagttgtcagagtgattGAAtccatgattggtctggtgcccaagcaccaacaatcatgtttttgtttgtgtgtttgttttttaagagacagggcctcactctgtcacccaggctagagtgcagtggtgccatcatagctcactgcagtctcaaactcctgggTTCaagcagtgctctctccacagcctcccaatgtgctgggattacaggcatgagacactgagcccagctgggatTTCttttggtgtGAAggccagtgatcagtcactagtaggttttgGAAggcacaataggtgtatagtggagaggcaaggTTCTTCagtgGAAaggatataagctTTCtagtcacaccagcctgagttaGAAtctgtcctctgccactttttacttgtatgacctcaatcaacttactttacTTCtgacTTCagtgtcctTTCtgtaaaatgagtctattaataatgatattgataatagctaaaattgagcatgtactatgtgccaggcattaccttgatTTCtctacatgcactgtctgattaaaTTCtcacaacaaccttagagaggtgggtcttgttactgctactgcctctgctttacagatggagGAAactgaggcaaggaGAAattaagtaactcgcccaaggtaaagggtggagcatggatttGAAtccacactTTCtgactacagactctacgatgtgatcttttttttaaaaacagctttattgagatataaTTCatataccatacaataaaagtgtgcaaccagtggcttttagtactgtataTTCacagagttgtgtggccatcaccacaaTTCattttaGAAcattTTCTTCaccccaaaagGAAactgcatacccattagcagtcactcctcatTTCccccaaactcccatagtcttaggGAAccagtaatctTTCagcctctgtggatttgcccctccttgacatTTCacataaatttgtcattttatgtcacataatttgtcattttatgtctgatTTCttttgcttgttttatatatatatgtgtgtgtgtgtgtgtgtacacacacacacacacacacacacacacacatgttTTCtggaggcagagtctcactctgtcgcccaggttggagtgcagtggcacaatctgggTTCactgcaacctccgcctccccagTTCaagtgattatcctgcTTCaccctccccagcagctgggattacaagtgccaccacacctggctaattttttgtgtttttagtagagatggggttttaccaggctggtcttGAActcctGAActcaagtgatccacctgcctcgacctcccacagtgctgagattacaggcgtgagccatcgtgcttggccctgcttatattatttttaaggatcatccatgttgtagcatgtgtagatactttggtccaccttattgttgagtaacagTTCattgtatggatataccacatTTCgtttatccagtcatcagttgatggacatttgggctgtTTCcacttgtttgctattttaaataatactgttatGAAcatttgtatacaagttgttgtgtacacatgtattTTCaattTTCttgagTTCtgtgatgtacttgttggttttgtttTTCtttttTTCtaccatgtgTTCgtaatgctcTTCtgagagGAAGAAaacagactTTCttacttgggagtggtcagcttatttgctgctacaatattaatgatgGAAaacccttatcaagagtgcttatcctgtgatgggtactgagcaatgctctccaGAAgcattactttaTTCagTTCtcaGAATTCtaatagggtagggatcttgacctcattttgcagataggcaaccaagtcttaccaagtGAActgTTCcagatcacagagcttggagagtgtgtagggggactgagcttagacccaggtgtgtgtgacggcctccaGAActcctgccactgtgttatgtggacaccattgtctacctcccactgGAAggtttaccagcctggattttacagagtgctTTCatggggtttgcctggtTTCaccctgtGAAatgggtgatgatgccatcgtttgatGAAgcagcagtgGAAttgagtgagcgcccaagctcatgggtGAAttgaggcgcagtcttggatggaggtctgtttttggagtttaaatttattgctgtttgcatTTCaacacaactgctttGAAagctacaggttgcctcaggcaccctgtcatcctTTCccaaTTCttttttttttttttttttgagactgagtTTCactctgtcacccaggctggagtgcagtgacactatctcagctcagtgcagcctctgcatcccaggTTCaagcgaTTCtcttgcctcagcctccagagtagctgggactacaggcatgtgccactacacccagcGAAtttttttattTTCagtagagatggggtTTCaccatgttggccaggctggtcttgatctcctgacctcaggtcatctgcccgccttggcctcccaaggtgctgggattacaggcgtgagccaccatgcccggccacctTTCtcaaTTCtttattatctatttaaatgcacgctaaaaaatttaccttaggtaaataaaggaGAAtgtggttaaaaaacacaactcagtaaacctggcactcTTCagtagtaggtctgggatctagttgtgttgaTTCtggtgacctatcacaaacccctctctaagcttattgctttttgctgttaaacttggtcataagcaagctgtgtccattgtGAAgtgtgtgctgtgagcacatcgcatacactagatttaatcatccactgccggcactcagtgcattgtGAAgggagggtaaggagctggtGAAatcacatgtGAAactGAAatacttgtgGAAtaaacgatGAAagggttttgtttgtaaaacaaacgctacaGAAagtattgttggatgcggtggcaactagtttattgctaccaagaggggtttaagtttGAAtagaccacaggtaagtgccctGAAgccaaaaggacttggtttataatgttaaaaattaTTCgtttttgGAAGAAcaagagttGAActcattTTCtgccTTCtctggGAAaaagcatTTCctgttttgttattagacagctgtTTCaaagttttgtccagtaagcaGAAttttgactaaaTTCTTCacaatagttaaaaccaatTTCctgggggcaggtgggGAActataaaagtcctcttTTCatctggaggtaggtttacctaccagcctaggccagctagctgtTTCtttacactatccctTTCtaaggatttgagTTCgtttgtaaaatgacatagcagacacaacataataccagagggggctGAAcgtggtggctcacatctgtaatcccagcactttggGAAgccaaggcagGAAgagtctcaggagTTCgagaccagcctgggcaacatagcgagaccttgtctctactaaaattaaaaaaaattagccaggtgtggtggcatgtgcctttaagtcccagctactccggggctgaggcaggaggaccgcttgagcccaggaggttgaggctgcagtgagccctgattgtgccactgcactccagcctgggcgacaGAAcaagatcctgtctcagacatacacacacacacacgcatgcacgtacgcacgcaccagaggGAAacatcatgcaGAAaaccattggcccatttattggatggtgtcctcccGAAgtTTCacatctgctgtttggagctactgaGAAcctggctgatTTCatttagtgctccctctgtcttactttgtctTTCcTTCctTTCtTTCctTTCtccctcctTTCcTTCtTTCcagctatccagtaacatgtattaaggactcagcatgtgGAAagcacgcatgacattgTTCatggagacgagggtctggagtcaggcagcagctcggatcccatctctgacagtggtgggctgtgtacactctctggcctcagcTTCcctatttgtaaaatggggatggtggtgctaactcacagggttgtgggcaTTCaggGAAaaattgtatatttagtactgggtgtggtgccctgccaaatgttaatgacTTCcTTCctctccagttTTCacacttaccttgtctcccatTTCtgaggtGAAtatgcattttaaggctcccaagccatagattaagctgttttgacatgtctgtccctgctgaGAAcgtGAAatcctgtgtgtgttattaaacagtttactgcccggccgcactgagtactcgcaatgtcttGAAcacccttgcaggttacccctcctggctctGAAcgggctgTTCcctctatgcaGAAtgcccacccacctgaccagttagtcatccTTCagcatggagctgtgggagagggagcctccctgtggttgggacatacctccctccaccccaggcctctcatcctcagttaacaacccgcctgtgccatcaattacactgttttgtggttttttacacctttttTTCtagacacaggatctcactgtattgccaaggctggagtgtggtggcatgatcatagctcactgtagcctccaactcctgtgctcaagtgatcctcctgcctcagtgtcctgagcagctaggactataggcacatgccactgcacctggctagattttaacatttTTCtttttgtagagataggatcttgctttgttgcctagcccagtcttGAActcctgggctcaactgatgctcccacTTCagcctcttaaagtgctgggattataggcttgagccaccatgggggctggcctcgcTTCacattttatgtagttgctataaccccaccactcaccctgctccagcccatgtcaccttTTCtctTTCctaaatgtactaagcttgtTTCccaccccagggcctttgcatttgctgTTCccgcagcaatctggagagctgTTCagattTTCctaggattaaTTCTTCccagtctgccatcccatggcacttattactacctgagatgatgatatatattgactcttgtctgctTTCtctgctaaaatcctccctgtgagGAAacgagtttggcacatatcccctggcagcatctgacatggcataagtactcagggagtatatgtcGAAtGAAtgagcctcttggtgggctcttgtacttgcactGAAttgtaattgcttgtttacacatcccacctccccactgggctgtgtgacTTCctttgcatcataacttggggtgatttgtctTTCtgtccacagggccaggtgcatggttgcttttgggtctgtaagtatgGAAgctagGAAtctttgaGAAggcacatccttaaacccataagctcTTCtcatggaggtagctgtccaccacaaaatgtccTTCtgatgccattggtaGAAcaagtactggttggagcaggctgcagttgTTCttacgTTCtgatggccatcctctactgtcccctcccccttTTCtttgtcaggtctttgccctgtgctgTTCtttggaccctGAAcaaacccTTCtccccaccccgctaatcctgtcctTTCcTTCagacctcatctcggtgtccctggctcatgGAAaccctccctggccaacctgccttgatcagatgctggctcctgcaaagcTTCctgagggcagtgctttatttgttttgctcTTCatttgTTCctcagagcacattgcacttggtcgacatttgttGAAtGAAtgagtaaaGAAttagtgatggcatatttacatacccatgTTCatggcagcgtttTTCactatagccaagaggtGAAagtaacccaagtgtctgttgacagatgagtggataaacaaaatatgtcatatacatacaatgGAAtattaTTCagccttaGAAagggagGAAcTTCtgacatacgccgccaccacgtagacGAAccttgaggacattatgctatgtGAAgtaagccaatcacagGAAgacaaatcttgtgtgagtccacttatatgatgtgtctaatgtagtcaaaTTCataGAAtcacaaaacaGAAtggtggttgccaggagctgaggggagaggGAAatggggagttgtttgataggcatgagtTTCagttttgcaagatGAAgagTTCttgagTTCagtataTTCtGAAgtgtGAAtatactactgtcttagtcaaTTCctactcctgtaactggGAAgtttataaaaaacaGAAatttatttgcccacagttttggctggGAAgttGAAgatcaaggcaacagcagatttggcatctggtgagggcagctctctccTTCcaagatggtgtcttgttgctgtatccttacatgacaGAAgaggtgGAAagcaagggggtttagctcagtccctccagcccttttgtaagagcacaaatcccaTTCatgaggttggagccctcttaatcacctcctaaaagctcctccccttaacacttactgtattgggtcttaggTTCcagcacatgagttttggtgggacacatacaTTCaaaccttagcaggtgctacactgtataccctaGAAaaatgagaggttaggagtgccgaccctcacacagtcaaaaatccatgtataacttttgactcccccaaaacttaactaatagcttgctgtttaccaGAAaacttaccataacataaacagtcaatGAAcgcattttatatgctatatgtattatatactgttttgtcacagtaaagtaagctaggGAAgaGAAaatacatttattactcactaagtgGAAgtggatagtcctaaaggacTTCatcctgattgtcTTCatgttgagtaggctgagGAAgatgacggtttggtcctgctgtgcccaggcggcagaggcgGAAgaggtggaggaggtGAAggggaggcaggggaggcaggcacactctgtataacttttattGAAaaaaacccatggataagtggaccagggcagTTCaaaccttgctgTTCaagggccaactgtacttaGAAatggttaagataataaattttaggttatataTTCTTCagcacaattaaaaaaaaGAAttactgctgctaGAAactatgacctctgGAAatcactgtaGAAGAAtctgGAATTCttaaaTTCtaggacacctctgacTTCtgggtagtatgcatGAActtaaggGAAaGAAgtatttatTTCctcatgaggataagctcaggtgttTTCcttaggactgtcagagtGAAggagtgggctggcctGAAccctccccgagttgcctgggTTCtcaggctcctctgcacctggagctagtgagccagcccatgggctGAAcctggctggtgtgtacagagcctgcacatgtctgcatagcctaggttgaggtttggacacttgcttagaTTCcaaggtcctcctttgtacccatttatttatataataaataatttattattatttttttgagacggagTTCactcttgttgcctaggctggagtgcaatggtgggatcttggctcactgcaggaGAAgtgaTTCtcctgcctcagtctcctgggtagctggggttacaggcacccgccaacatgcccggattttttgtgtgtgtttttagtagagacgaggtTTCaacatgttggccaggctggtcttGAActcctggcctcaggtgatcctcccacctcagcctcccaaagtgctgggattataggcatgagccacggtgcccagcctggattttttttttttttttttttttttgagacagggtctcgctctatggcccaggctgGAAtgcagtggcccgatctcaggtcactgcaacctccgcctcctgggctcaggtgatcctcctacctcagcctcccaagtatctgggactacaggtgtgcacaaccatgcccagctaatttTTCtatttttagtagagatggggtTTCaccatgttggccaggctggtcttGAAcTTCtggctcaagcgatctgcccatctcagcctcccaaagtgctaggattacaggcgtgagtgactgcgcctggcaaagtgtaggatttGAAacataaacttgtaTTCatttttgatctcatttaactgtcataacatGAAGAActcaactcttaggGAAggacacacatTTCccagtgcctgtccagcaccagcacgagtgctttgtagagattgcctcatTTCttgccgacattgcccTTCtGAAgtgtagatggttttatcgccactgtataaaGAAgggtatagtgtatccaacgccccacagctaagtgacaggacaaggatctcactccagatcttgtgactttaaaaatcactTTCttggctctgcTTCtccttgtTTCctagatggtggccatctcatggtccagatttatatacatctcagcctcaaatcaaatggcacactttacatttgcacgctgtttggccaagcccattggcatataacatacactatggtagcaaGAAttggccagaggctccccaagaccagacTTCtGAATTCctaaccccagctcccaagccccaggcatcccataaaatctttgctagatgTTCaGAAacctaaaatttgcTTCtccacccctcccTTCtccccaagtggtaaagacaaaGAAcagacggcagggctctttgttaTTCttttatctgctaagtttacaaaaaggttagacagtgGAActttgctgagcaagcaTTCtgtccctgagttgtctctgagcagcaccaaatgagtcttatcTTCcccttgttatctccaaggacgtaaaacattTTCttTTCatgctttttaatTTCtgtgtaacattagccaTTCagttgacctGAAacattgctagtttatTTCtagctggcgtgtgtgtgtgtgtatcagagggtctatactgtataaatacTTCtctaaataTTCttTTCcataatgttgctctaaagtgtctcatttactcaaTTCtgtcaacttagatgctGAAtcattttatgtgcttaaaaGAAatatctactcggatgctctagTTCcctggcaagggagagagagggagaGAAaGAAagctgttGAAgtggtgcaaatccaaactgctTTCttttttgctcataagtagtgcttTTCaaagcgtttggcatccctgccatatggatgtgtcactgtgtgagatgacaacaggatttgctgGAAatgtgggccactattgtatccgccttacgtaaccacatggagtgatgGAAatgggtgagcagcccgGAAtgtaggcggtggcctggcccatgcatctccagagaccatGAAgactgctcaagccctacataggatgtggatccaggctgttaaaaagttgaggagatgGAAaggccgtgtgagtccctctgcaagctctccccttgttTTCcccaacctTTCTTCatggGAAggggaggGAAgcaaaaccaTTCtcacagtgagtcctttatcctcagattgatttgtgcTTCtggcatctTTCagcaacaaattgtgagattgtaggttgtagtcactggtgggtttttaaaaatacttgtattaattttagcagatctatgtGAAactgatagcctcttactggatctgtggatcaaaggctgctgttTTCcatgtgctagGAAtctgtaatggttttgcTTCctgggtagagcttaacatactggcttttgtgtttgTTCatgtttgaGAAtTTCttgttttgggataaaactttttgctTTCttacactgcaGAAatctataTTCttattatgtctaggcTTCtaacttaGAAGAAaaagtgtttTTCacatgTTCttttTTCctcttacagtttGAAcatgtgtgagtgtttactttgatggcttgagacttttatGAAgcatgtcccttacgtggacagcttgcgattaatctttggttttagctttgacctccatcactgggcagcagcagtctgtggatacaGAAtctaaaagccgcctgTTCagggggactttgggcagtGAAcacTTCggtatctgttttggggttggcttttaacatgtTTCtacaTTCtttatgtaaaatatataggatttttacctatctggccaagatttTTCaaagGAAaaaagctttataaaaagattaaaaaaaaacacaataagtaactatttgccttagTTCctTTCtcTTCttttgtcacacaaaaaccttactgttaatgcattaagttttttgtttatggtaGAAaagttagctcacttttaactTTCcatgaggtcttaaGAAtgacctgttagatataaagggggttgtcatGAAcactacttacgtGAAaagtaaaagtgataagGAAagatgatGAAagcacattGAAgatcctcctaatggataGAAtgttgacacttggattaaaaaggtgacttggcgttTTCtgcatacacctccccccacaactccccagacctaacctgcgtccccactctgcccaccgcacctgccttgctttaccctctttgcccctTTCaaattactggtaccatccTTCaactcgccctccTTCcagttGAAattattatcccctcTTCactttgcaaaaTTCtaactgcattGAAaaatttgctgttaaatatactcgtacctcttgggtattagatccTTCaccaggagtcagtaTTCattttggtgTTCacccactcatctccctattttttatttatttatttatttatttaatttttttatTTCtttttgagacggagtcttgctctgttgcccaggctggagtgcagtggcgcgatTTCggctcactgcaagctccacctcccgggTTCgagccaTTCtcctgcctcagcctcccgagtagctgggactacaggtgcctgccaccacacctggctaattttttgtatttttagtagagatggggtttaaccatgttagtcagtatggtcttgatctcctgacctcgtgatccacccgcctcggcctcccaaagtgctgggattacaggcgcgagccatcgcgcccggcctcatctccctattttttaaacttatGAAatgtttggtaccataaaaGAAttaaatatctaaatcatggagcataataattaaacaaatgccatgagcccattacacatatctgcagaccatcaccaagacaaaacacGAAgGAAatttgtgtacctcccaccatactctgcctcctgcccaggtcacctcatcctGAAtgttatgtttTTCatTTCcttgctttTTCtttatatTTCcactgcatgcgtgcgtacacacacacacgcccctaaacaatagattaattttgttggcaattttttactatagtGAAaaacgcataccaaaatttactgtctgtaccatttgtaaatgtacagTTCagtagtgttaagtataTTCaagttgttgtaaaacattgttttttgagctatataaaaatggtatcacactctacaaatccTTCtggggtgtttttttgctcaactttatgtttgagatgcatcccttttgtggtggtctatgtgactggtttgTTCgtcTTCattgcagtaaaacattgtattttaataatgtatcaatttgtccatcctgcccttgagagatactgggatattTTCcaagttttggctgttggGAAcattgctgctgtGAAtactctatacatgtgtccttgtgcatttagtcacacggTTCtcccggtgcctctgcccaggtagagGAAtcactgggtcgtgGAAtgtgcatacctttaaTTCtgatatatgcgtgctctTTCtatgggcctgcctgtaaagctccccctggtgctaagcagctacctgctgtcaTTCagagagaggtcagatattgctagcaagtctgatctgcgcatagccaagcacaaggtggtgggtgggaccagagcccggtggcctagtttGAAtgtcaggaccacagctctcagctaagctgctgcaccctggcagcctgtgcccagggatgTTCctagagctgatgTTCtgagGAAtaaggatgattatgtggggtgctctgtgtTTCtGAAaaattatataaatgaggcagGAAagacactcagtagtttgctctttgggtacacttgtagttagttggtaagctgcccaagggctttggcgattttaTTCactgatgtctctGAAgtgccacaaaccacttgcttggcaGAAggtggatgcttaataaatgtttttgcataaatGAAcaaatgagtgcacagtGAAgagttTTCtgtgagaccTTCccaaaaagtcacagTTCtgtaggctaaatatacatagttatggtgtatttttaatgTTCaacagagctactaaaatttatttttgctctagTTCccTTCctttacaattaagattTTCtctttatcacttggcttagcagagcatataTTCtttGAAtattatgttaagtagctaatagttaattttattacccgttaaccataggtaaGAAGAAgcctcgcaatctgattggtTTCaggcaccaggcaagttaatagcaaggtctgattggtggtggtagtggtaggagattaatcgtctctctgacaaatttaatttaaattacgcttgcaggcatcacaggacacttaaagatggtagcatcccattattGAAgggctgtGAAtTTCaaaagccagaTTCaTTCtacttgagccacatttaaaaaataaaattttgcagGAActcatttatttggctaaatgataaacaaatttaaatccaaaactttGAAgagtttatagttttaaaTTCcaaaataGAAatgtattttaaaagtaatgtactcataaaaataTTCtctataatagcacagctccttgggataTTCaacatttttttggtatgtagcattgcccaatcctggggatgattgtcatagtcccagttagttttttTTCctgataattttaaagtaagttttgtggggaggatgtcatgtaattttttTTCccccttaaTTCatggggctataactatatccaaaGAAgagttggcaaatatatttattttaccacaacTTCtagattaaTTCactttgttGAAatttgacagctttaaggtacaaGAAggcattgaGAAgatgagcttggaGAAgtgggcatgctcataaggtcatTTCtgggtggtagtggcttagttttactaaggccacatctaaaactGAAgttggcaaggagttagtaaggtgTTCtatgGAAatgGAAaagggcaaccaaTTCtgacttactgcaggagTTCagttgagtgtctgctTTCtttTTCttTTCtTTCtttattttttttgagacagagtctcactctggagtgcagtggcgcaatctcagctcattgcaacctccacctcccaggTTCaagtgaTTCtcttgcTTCagcctcccaagtagctgggactacagatatgcgccaccacgcccggctaattttttgtgtttttggtagagatagggtTTCaccatgttggccaggctggtcttGAActcctggccccaagtgatccacctgccttggcctcccaaagtgttgggattataggcgtGAAccatcacgcccgacccggtTTCtgctTTCtgtTTCcatttaaGAAaactttttggccgggcgtagtggctcacacctgtaatcccagcgctttgggaggccgaggtgggcggatcatctaaggttgggagtttgagaccagcctgaccagcatggtGAAaccctgtctctactaaaaatacaaaaattagctgggtgtggtggcgcatgcctgtaatcccaactactcgggaggctgaggcaggaGAAtcgctaGAAcccaggaggcggaggttgcagtgagcccagatcgtgccattgcactccagcctgggctggagcaaaactccatctcaaaaaaaaaaaacaaaaaaactttttaacaaatatgtacaacTTCattaaaatattgGAAaatttattgtgcTTCcacatattgGAAaatgctgttttagacacatgcaGAATTCcagagaggtatggctatagagcacatctaGAAagtcagaccatgagcacctgagtttttagTTCacccacctcacgtatgctatcaaaGAAgcagagagccacGAAtttgatacaaactTTCtttaaGAAaatttgtaggctgggcacggtgtctcacacctgtaatcccggcattttgggaggctaaggcaggcggaccatttgaggtcaggagtttgagatgagcctggccaacatggtGAAaccgtgtctctaccaaaaatacaaaaaaatgagctgggtgtggtggcgcacaactgtaatcccagctactcgggaggctgaggtatgcGAAtcacttGAAcccaggaggcagaggttgcagtGAAcctagatcacacccctgcaTTCcagcctgggtgacagagtgagaccccgtccccccccccccccaaaaaaaaaaaaagtagcagcagtcagtgcTTCagttatattaattGAAtgatGAAtGAAtagtgtatccatctctagatgtattaaaaaGAAaaaactttgagtgcctactctgtgtgccagacactgttgtagcagataatgggacaaagatgagtaggatacaaaaatTTCacctGAAaaTTCacttttattttttaaaattGAAGAAcagtcgtcacagtGAAcagactttaagtaattggggagttttacataatttagataataatcttttgGAAtgtttacatatTTCcttgtgttgatgggccTTCatttaatctaaatcttgcacttttgggctgcttgcgtccctggcaccccaggccccaTTCaccacccagtcccaggattgctggtagcacagatctcTTCTTCtggcctttTTCaggtattttgagTTCtttttttttttttttttttTTCttattaaatatacagatggggtctcactatgttgctcaggttggtcgtaaactcctgcactcaactgatcaccccgcctcagcctcctgagtagctgggactatggcatgtgccaccatgcctggctaactttaaaaattttttgtagagatggggtctcactatgttgcacaggctgcccttGAActcctaggctcaagcgatcctctcgcTTCagcctgagtgctgGAAatacagacgtgagccactgcacccagccctcTTCtaatTTCttaaaacataaacacatagtccgggtggcttggtggctcatgcctgtaatcccagcactttgggaggccgaggcaggtggatcacaaggtcaggagtttgagaccagcctgatcaacatggtGAAgccccatctctactaaaatacaaaaattagccgggcgtggtagtgcgcacctgtaatcctagctacccaggagactgagtcaggaGAAtcgcttGAAcctgggaggcggaggttgcagtgagtGAAgatcacgccactgtactccagcctgggcgacaGAAcgagactccgtctcaaacaaaacaaaacaaaacaaaaaaccaacatatacacatattatagatactccatttttgtctgtggctggtatTTCaaatatttaaagtctttgaTTCTTCagtttatgtTTCttttacctctcactcatggtgatgtgcatgtttaaattgtgggctcatTTCcttggatacatactatctgggGAAtctttgagggctgtgtttgagtgtgatctctggagaggaTTCgcatctgcctctgtgtacttggtggtatggccagtgcagagccccagtaggcatgagtgtttggcagggtgtggtcacgtctgtaatatGAAcgcccaaacctgtgtGAAtacaggtctgttgataGAAaTTCtccagggttttatttttagttatTTCcactctacccatagccaaggatgagacagtcactgcatctccatctgtgtgctggtatttttTTCccctaatagatccacccagacctgatctcccaccctgtttggatccccagcctcctctaaatgcctgcTTCagtgctggcttatctctgtggTTCtgagtgTTCttggcatTTCtagctTTCtcacaggcagcctgcatttaatggattattatttttaaaatgataccaccagttgtagttttTTCtggatacctactttgGAAtattgtcagagatgGAAgtcccagtggtctatttatGAAgacaTTCatctTTCatactcagtgatacagatggaGAAaaagccGAAagatcccgcagatcacccaattgggcataccctatgataaggctaaggatatttttagacataaacatgcaGAAaacttgcctacgaggtcacctttgacagGAAgagggggaGAAtgattttaaaaaaaGAAtctatttGAAactagatatttttaaaggggttttatgatgataatgttttgGAAtagtgataataaaaggactcagcacttaacaTTCcaTTCtttttagggtctgtctctcactatgtatTTCacttacctTTCaagtattgtTTCtagtTTCatTTCattGAAggagagtgtgtttGAAaataggtagcataatGAAcagatattaactcttttttggtataGAAagtgtgagctggactttatgtcgaggcattttGAAtgataattttatTTCtagcattGAAaaggttgtggttTTCatcagattggttttgTTCttttaaaacag  ***TJP2*_Intron 2** gtgagTTCtgcacatTTCatataTTCtacagtcatgTTCttgattttacggatcatggatcttatttattTTCacctacacacagttttggctttttttTTCccttgaggtacataatgtattatagatactaactggcaataattgctatgttaacaaagatttgcatgctattgtattttttgattaaggactgtttagtatgGAAcattttagaggttatttagtgtatactcttatctccagctaGAAcaggttatGAAagagaGAAaaGAAaggGAAGAAtGAAaatgacctgtactgtttaaggatgtggagggagctttttacaacTTCtgtaattttaattatgacatattatacataccttataactcttaaaaagtgtagcatccaagagactgtaagtcatttgtgtgaggctgagctggaGAActgcactcgtggTTCtgcatttttttttttttttttttttttttgagacgGAAttgcactcttgtcgcccaggctggagtacggtggtgcaatTTCggctcagcgcaacctccgcctcccgggTTCaagcaattTTCctgcctcagcctcccaagtagctgggattacaggcgcctatcaccacgcccggctaattttgtatttttaatagagacggggtTTCtccatgttggtcaggctggtctcGAActcctgacctcaggtgatccacccaccttggcccgcaaagtgctgggttgcaggcgtgagccaccgtgcccagacggTTCcgcatttttaTTCcgtcaagctgccTTCcggtcatcatcactgtgattTTCctggtttTTCtgcagggtTTCTTCTTCtggctctGAATTCctccaaggtctgacatctcctagagggaccataagTTCctcatagGAAgagttTTCctttgaccctcaGAActgtgagcTTCaagctttgtcTTCtatGAAaattaaagacaTTCccGAAtttaatgtttgttGAAttaatccaggtaGAAaaaaatgtctctgggccagtgcccaggtacccagacTTCcagatcagtaatcacaatTTCctcatttgaggggttttgtctgcttaggtcttatttTTCaactTTCctcgtctTTCtgactctcctctgccctTTCcctgagcatTTCatgcaTTCcttgagttgtggacccaaaattggcagaggtcagagatttttttaggcttggcatagaggatggagctgcctctgtctaaaaccaggtgccggctggtggtgctgccaaaTTCaaagccatgtgattaatagacaacacccccaacctgttttgagtttgtaattgtgtataatcagtcagcatagtgggcctcacgtggGAAactTTCcttgGAAagGAAgattTTCttttagtctcggagtaaatttttatataatcGAAcccagatttaaGAAtatacagtcaaatatactcatattatctggatGAAactgggcttagatacatatatatatttttGAAatggagtctcaTTCagttgcccaggctggagtgtaattgcatgatctcagctcactgcaaccTTCacctcctaggTTCaagtgattTTCctgctccagcctcctaagtagctgggattacaggggtgtgccaccacacctggctaatttttgtatttttagtagagatggggttttgccatgttggccaggctgatctcGAActcctggactcaagcaaTTCacccgccttggcctcccGAAgtgctgggattacaggtgtgagccaccgcacctggcccagacttagctttttaaaacacaaatGAAttaggattatgtattatctatgctcaccattgtgcattgctagactgatcatggatgtaataaatcttatTTCcgatcTTCcatgcatGAAGAAaagggtttagtgctttgcttTTCatgagatctcaacacatattttacgTTCaaattttatctttaaaatccagttacgggccgggcgcagtagctcacgcctgtaatccccacactttgggaggcagagacaggtggatcacaaggtcaggagatcgagaccatcctggccaacatggtGAAaccctccctgtctctctactaaaaatacaaaaattagctgggtgtggtggcacatgcctgtagtcccagctactcaggaggctgaggcaggaGAAtcacttGAAcctaggaggcaggggttgcagtgagccagcaccactgcactccagcctggcaacagagcaagactccatctcaaaaaaaaaaaaaacaaacaaatccatttacTTCtttTTCactgtatgttGAAatGAAactaccaacacatctactttGAAgttaaaggtatactttaaaaatataattttaggcagacataaGAAaGAAcGAAatatgtcctttgcagcaacacGAAtgtacctggaggccattatcctaagcaaattaatgctGAAacaGAAaaccaaatactgcatgTTCtcacttaGAAgtgggagctaaacattgggtacacacagacacaaggacagGAAcagtaaacactggGAATTCcaaaagtacggagggagggagaggagcaaagtttGAAaaactacTTCttgggtactgtgTTCggtacttgagtgacaggatcattaGAAgcccaaacctcagcatcagacaatacacatatataacaaacctgcacatgtactcctGAAtctaaaataaaaataaaattaaaaaggattggctagcactggGAAgagcagtcttTTCccgcccagctaGAAagttgtggttttttttttttgttttttagagacTTCtccatttgcccaatttaaagtgcaaaagctgccttggcttgttGAAgccttgacctcacaggctcaggtgaTTCtcccacctcagcctcccaagtatctgggactatggatgtgcaccaccatgaccagctatttttTTCtatTTCtagtaaagacagggttttgccatgttgtccaggctggtcttgagctgctggactcaagcaatctgcctgcattggcctcccaaagtgctgggattatagacatgagccatgagcctaggcaGAAggtttttttttttttaatatgtcaaaacatcacaatatacaGAAGAAttaaaaatatttacagtacaggccaggcacaatggctgtctgttgtaatcccagtgctttgggaggttgagacaggaggctcatttgagcccaGAAatttgaggctgtagtgagtagtgatcacaccgctgcactccagcctagcaacagatcaaaactctgtctcttaaaaaacaaatacaataagGAAatctGAAaattaaaaaatacagttttagtacaggTTCagtttttactGAAgttgTTCcagctcgggccagcatgatgcaaagaggtacaactggtgcTTCaaacagtacgtccctatttggactGAAgacattgtTTCctatcccggccagcTTCtagtgataactccagtctgcactatcaatcttTTCcagcctctacaggctgtTTCtTTCagataagGAAtTTCcctttataaacctgtcagctgacaatgactatgctttgttttgtTTCtcatttgtTTCtgtaagcccatctgTTCctGAAcagtctctggttattgatttgacctttattttattgagtgcttgtaataaatcctGAAagccacttattGAAggatttttaatatTTCtcctctctgatgtacag  ***TJP2*_3’ UTR (5 kb downstream of the last exon)**  ctttgtggtggatttgtatgctacTTCctatttttaaatgcaacttttatTTCagttactaatcataGAAgtgattactaacctccttgtcGAAcattgGAAaatgGAAacgtGAAaaaGAAaattatacccataatcccatccctcagataattttggtgtattTTCtccaattacgtatttgtggatgtctgtgtgtatgatacttgagggtgtactgtgtttgtagTTCtatTTCctattttTTCtgtttatcaTTCtatcactaacatTTCtcaagtggttacaactgtgcagcaaggtatttaaagcgtggtgtacacatcatcattaacaaccacagagcaatatatcccatggctgcTTCataagtGAAcaaTTCtgttgttggctgTTCggattatTTCcagttgtttgccttttttttttttttttttttttttaaacatGAAatgcacctctttgtatgtaatccagttttgtatatcattgatcagcatTTCagtatttTTCtgcaggcaagTTCtcaGAAgtaGAAttactgagtaaaaGAAtagGAAcatttttaaggcccttgatgtttactgccaaatagcTTCctaGAAaagttgcatcaatttacacccccacatccactcTTCctcTTCctcTTCTTCctcctcctcctcctcTTCtTTCttTTCctTTCctTTCttTTCctTTCtTTCctcctcccTTCccccctccctcccccccTTCcTTCcTTCtTTCcTTCcTTCctccctccctcccTTCtctctctctctTTCtTTCtctctTTCcctttttttttttttttgacagggtcttgctttgctgcccccaggctggtcctgTTCttGAActcctttagcaatcctcccacctcagcTTCccaaagtgctgggattacaggcgtgagccactatgcccaaacaccacttacaTTCttGAAgtactaaaataaatttaGAAtttaagcaagacagattagGAAtTTCggcttgtattaataaaaaGAAcaatGAAtttgGAAaaggaGAAaagagtcttggTTCcattaagggtcttttatgatatGAAttgacacaaaggagttaggcttgtgttttagtggtggcaGAAatttggcttactggtaactgagttatactataaaggtgcccttgcttatgtctcTTCcaatatgggtgctgtcacttataaatcatttaatgtatctcGAAtTTCtGAAcactcctcagggcattgactaaaatttgcataTTCcataaataaaagccaccttttgtGAAcaggaGAAaaatgtactagttaactatatgataagactgtttgtttgtgTTCtttaaatccagGAAtgagccctttgcaaacataggtggggaggcgaggaggctTTCagcaggagctgggttgtcgtctggTTCcagcaattttgtgatgcgggcagtgccactgTTCctcaGAAtgtccagcagtgtgcctgatggacccccagtgccTTCaccacccctttttaTTCatatcctcctttgTTCtggGAAagatttaaagctgcctacagtatacatgGAAcataatGAAaaaaaaaaaatgatgctaagcaaatGAAGAAacagggaGAAgagtatcagtagaggGAAgatagagccatgagtgagagccgtgTTCattTTCtgcgccgtcaggactctgcagctctccaGAAgccagtgtggccccagcggcacctgcatctatgataGAAttatgcctgtgcTTCtagcccagtgagatagatgagcgatgggtcTTCcaGAAtgacatcagatgctactgatgtcactaacatcagtagtaatttgacactaacaaatTTCtttatatGAAgtaaagttggttagGAAcagtccatttaagcaatcgcagctgtcttttaagaGAAaggTTCccagtcaatcTTCtccctccttTTCGAAcctcttTTCagttggggtcattttgccagTTCtagctTTCatatacatatgtGAAgaGAAagagatttattttaatGAAttggcatacagggttgtaggggattggtaagtctaaaatctGAAgggtagaccagcaggctggagacccaggggagagatgcaatccaatcctcctagctggtgtgaggtcagtctttgTTCtgttttaaggccTTCaactgattggatgaggccgacctacatTTCagagggcaatctgctttactcaaagtccaccaatttgagtgttaatctcctaaataaaacaacaacaacaacGAAacttttactGAAacaaccataataatgtttgaccaaatatttgggcatcatggcccggccaagttgatgcataaaaattaaccatcgcagtccctcatcctgtttaccatgtaggccaccctacagccatcctttttgtttagtgttaaagcatgagtGAAtGAAtGAAccaGAAaaagggaGAAttatttacatacatatacacgtatgtataggttttgtacaatatatatactctggatatacatgtattataaattttgtttttgttttattattttttgagacacagtTTCactgacccaggcttaagtgcagtggtacagtcacaactcactgcagcTTCgatctcctggaTTCaagcagtcctctcacctcagcctcccaagtagctgggactacaggtgtgtgccagcatgcccagctaatttttgtattttttttgtagaGAAgaggtTTCaccatgttgcccagactggtcttGAActcctgggctcaagcgatccatccactttggcctcccaaagtgctgGAActacagactacaGAActacagagccaccgcacccggcctatattttgttttaaataaagatgagcaaatttgatGAAtaGAAaagcattaaaaatgggctggcgtggtggcttatgcctgtaatcccagcattttgggatgccaaggcaggtggatcatctaaggttaggggTTCgagaccagcctggccaacatggtgagccaccgtctctactaaaaatacaGAAaattagctgggcgtggtggcgggtgcctgtaatcccagctacttgagaggctGAAgcaggaGAAtcgcttGAActcgagaggcagaggttgcagtgagccgagatggctccattgtactccagcctggacaacaagagcaaaactctgtctcaaaaaaaaaaaaaaaGAAaagccttaaaaatGAAGAAacaaaaatGAAtGAAatttGAAtaatagagctGAAactccaataaaaataaaaatacTTCatatttaaaaataatatttagtGAAataaatgagacaaaaggctGAAtggcaaataatatgtatatatgtttgtgtatatatacagagacagataaaacatatTTCcatcctgctttttattttaaatctcatttatatatacatagataGAAaattggcacattttataTTCcccacactagtgactgcaaaggcacttatagcactttggcatttattttgcgcaataaGAAgtgGAAtgtggtttgattgcTTCtgcactgtcctcggatgccgTTCcaccTTCgagagtccaggttgctctgtctTTCtgagtttgagtctgtctagGAAggataactgggGAAGAAgatcctgactGAAagtatcctggggccaggtgcggtggctcatgcctgtaatcccaccactttgagaggctgaggcaggtggatcacttgaggccaagagTTCgagaccagcctggccaacacggtGAAaccctgtctctaagtaaaatacaaaaattagcagggcgtggtggcaagtacctgtaatcccagctactcaggaggctgaggcaggaGAAtcgcttGAAcgcaggaggcagaggctgcagtaagctgagatcacaccactgcactccaccctgggcaacagagcgagactccgtctcaaaaaaaaaaaacaccaaaaagaggccgggcacggtgactcacgcctgtagtcccagcactttggaggccaaggggagtGAAtcacctgaggtcaggagTTCaagaccagcctggccaacatggcGAAaccccatctctactaaaaatacaaaaattagccaggtgtaatggtgcttgcttgtaatcccagctactcaggagactgaggctggaGAAtcgcttGAAcctgggaggtggaggttgcagtgagccgagatcgcaccattgcactccagtctgggtgaGAAaagcGAAactccatctcGAAaaacaaaaataaaaacaGAAaGAAagtatcccgagagacatgggttgagctttGAAaatgcagctgtcccttggtgtctgtggaggatgtTTCctatgacctcccatggataccaaaatccagcgatgcccaggtctttgatataatggtgtggtaggccgagtacaatggctcatgcatgtaatcccagcattttgGAAggctgaggggGAAggatcgcttgaggccagTTCacctgggcaacatagtgagacaccacctctaaaataaaaaataaattagctcagcatggtgtcgcgtgcctgtggtcccagcTTCtctggagactaaggtgGAAagatggcttgagTTCaagagtgtgaggctgcagtgagctatgatcacacctctgcactccagcctgggtggcaGAAcgagatgctgtctcaaaaaaataaataaaataaaatggtgtggtatttgcatataacctacatacatcctcctgtatactttaaatcatctccagattacttatagtagccaatacagtataaatactgggtaaatcgttgttatcttgtattgtttTTCtatttgtattttttattgtttTTCtttacccccgggGAAtatttttggtctgaggttggttGAAtccgaggatgcaGAAcctgcaggtatagagggcccagtgtccaggagacccacctctgaggTTCaggggagagagGAAtggagaGAAcctggacctgGAAGAAaggagg |
